# The Fox/Forkhead transcription factor family of the hemichordate *Saccoglossus kowalevskii*

**DOI:** 10.1186/2041-9139-5-17

**Published:** 2014-05-07

**Authors:** Jens H Fritzenwanker, John Gerhart, Robert M Freeman, Christopher J Lowe

**Affiliations:** 1 Hopkins Marine Station of Stanford University, 120 Oceanview Boulevard, Pacific Grove, CA 93950, USA; 2 Department of Molecular and Cell Biology, University of California, 142 Life Sciences Addition #3200, Berkeley, CA 94720, USA; 3 Department of Systems Biology, Harvard Medical School, 200 Longwood Avenue, Warren Alpert 536, Boston, MA 02115, USA

**Keywords:** Hemichordate, *Saccoglossus kowalevskii*, Fox cluster, Deuterostome evolution, Gene regulatory networks, Gill slits, Gut patterning, EH-I-like motif, *FoxQ2*, Fox genes, Forkhead, Fork head

## Abstract

**Background:**

The Fox gene family is a large family of transcription factors that arose early in organismal evolution dating back to at least the common ancestor of metazoans and fungi. They are key components of many gene regulatory networks essential for embryonic development. Although much is known about the role of Fox genes during vertebrate development, comprehensive comparative studies outside vertebrates are sparse. We have characterized the Fox transcription factor gene family from the genome of the enteropneust hemichordate *Saccoglossus kowalevskii*, including phylogenetic analysis, genomic organization, and expression analysis during early development. Hemichordates are a sister group to echinoderms, closely related to chordates and are a key group for tracing the evolution of gene regulatory mechanisms likely to have been important in the diversification of the deuterostome phyla.

**Results:**

Of the 22 Fox gene families that were likely present in the last common ancestor of all deuterostomes, *S. kowalevskii* has a single ortholog of each group except FoxH, which we were unable to detect, and FoxQ2, which has three paralogs. A phylogenetic analysis of the FoxQ2 family identified an ancestral duplication in the FoxQ2 lineage at the base of the bilaterians. The expression analyses of all 23 Fox genes of *S. kowalevskii* provide insights into the evolution of components of the regulatory networks for the development of pharyngeal gill slits (*foxC*, *foxL1*, and *foxI*), mesoderm patterning (*foxD*, *foxF*, *foxG*), hindgut development (*foxD*, *foxI*), cilia formation (*foxJ1*), and patterning of the embryonic apical territory (*foxQ2*).

**Conclusions:**

Comparisons of our results with data from echinoderms, chordates, and other bilaterians help to develop hypotheses about the developmental roles of Fox genes that likely characterized ancestral deuterostomes and bilaterians, and more recent clade-specific innovations.

## Background

The development of animal body plans and associated morphological innovations are a result of genetic and cellular mechanisms acting in space and time. Developmental regulation of these mechanisms has many layers of complexity, and involves interacting suites of transcription factors that form core conserved regulatory kernels [1]. Analyzing these transcription factors and their genetic interactions is therefore essential to understand development, and comparative studies between species can help us to understand the evolutionary traits of developmental programs/networks and how they arose during evolution [2-6].

One large transcription factor family with key regulatory roles is the Fox (Forkhead box) transcription factor family. Fox genes encode transcription factors containing a fork head helix-turn-helix DNA binding domain of 100 amino acids [7-12]. The conserved protein sequence encoding the DNA binding domain was described in 1990 by comparative analysis of the *Drosophila melanogaster* ‘Forkhead’ protein [13] with the HNF-3 protein isolated from rats [14,15] by Weigel et al. [16]. In the 20 years since their discovery, a large number of Fox genes have been characterized in a phylogenetically broad range of animals, including choanoflagellates, yeast, and fungi (reviewed in Larroux et al. [17]) and a unified nomenclature of 15 Fox families (alternatively named classes or subclasses) was established in 2000 [18]. Five more families have since been added: FoxAB [19-21], FoxQ [22-24], FoxP [25,26], and the vertebrate specific groups FoxR [27,28] and FoxS [29]. Four of these families have subsequently been further divided: FoxL into FoxL1 and FoxL2, FoxN into FoxN1/4 and FoxN2/3, FoxQ into FoxQ1 and FoxQ2, and FoxJ into FoxJ1 and FoxJ2 [30]. This has led to the identification of a total number of 24 Fox families, making it possible to compare their orthologs in different species to gain insights into the evolution of this large transcription factor family and their roles in metazoan developmental programs.

Fox genes probably arose by serial duplication from a single ancestral Fox gene present in the fungal/metazoan ancestor (stem opisthokont) [17,18]. This evolutionary history is reflected in the clustered arrangement of some of the Fox genes in animal genomes [31-33]. By comparative genomic analysis two Fox gene clusters have been proposed to be present in stem bilaterians; a *foxD*-*foxE* cluster and a *foxL1*-*foxC*-*foxF*-*foxQ1* cluster [31,33]. The latter is of special interest since its conserved linkage may be correlated with its function in mesoderm development [31,32,34]. Much of the literature on Fox genes focuses on medically relevant developmental roles using data from a narrow range of vertebrate model systems including only a few invertebrates like *Drosophila melanogaster*[35-38] and *Caenorhabditis elegans*[39-42]. Recently, a more extensive evolutionary comparative literature has begun to emerge; new data from animals such as elasmobranchs (dogfish) [34], echinoderms [20,43-48], cephalochordates [21,49-55], urochordates [56-60], lophotrochozoans [32,61], cnidarians [19,62-65], and sponges [66] make it now possible to investigate the deeper evolutionary history of this transcription factor family.

In this study, we have characterized the full Fox gene complement of the enteropneust hemichordate *Saccoglossus kowalevskii* to contribute to this discussion. Hemichordates are a deuterostome phylum, sister group to echinoderms, which together form the Ambulacraria [67-70]. Hemichordates share many organizational features with chordates such as a bilateral body plan with a conserved anterior posterior patterning gene regulatory network [71-73]. Their anterior gut is perforated by pharyngeal gill slits, likely homologs to those of chordates [74-79] and they have a nervous system with both diffuse and central organizational elements [71,80-87].

In our study, we identified 23 Fox genes in *S. kowalevskii* and analyzed their phylogenetic relationships, genomic cluster organization, and spatiotemporal expression patterns during early development. The expression analysis of all 23 genes gives insights into the evolution of components of the regulatory networks for pharyngeal gill slits (*foxC*, *foxL1*, and *foxI*), mesoderm patterning (*foxD*, *foxF*, *foxG*), hindgut development (*foxD*, *foxI*), cilia formation (*foxJ1*), and patterning of the apical territory (*foxQ2*).

## Methods

### Embryo collection

Adult *S. kowalevskii* were collected at Waquoit Bay, Massachusetts in September. Oocyte ovulation and fertilization were carried out as described previously [88]. Embryos were staged by the normal tables of Bateson [74,89,90] and Colwin and Colwin [91]. Embryos were cultured at 20°C.

### Identification and cloning of Fox genes

Numerous Fox genes were identified by screening expressed sequence tags (EST) [92], and expression patterns of *foxQ2-1*, *foxG* (*bf1*), *foxA*, and *foxC* in select developmental stages have previously been published [71,79,93]. To identify and clone additional genes, we screened the *S. kowalevskii* genome-trace archive at NCBI and our arrayed EST clone libraries [71] by bidirectional blast. Genes not in our EST libraries were cloned by PCR from cDNA prepared from a variety of developmental stages, using RNAeasy (Qiagen) for RNA extraction and Superscript III (Invitrogen) for cDNA synthesis, and cloned into the pGemT easy vector system (Promega). Primers used to clone partial fragment of FoxJ2/3: 5′-CAATGGACTGGCTGCCACAACTA-3′, 5′-GTGTGAAGAACTGATTGAGTGAATTTGC-3′.

### In situ *hybridization*

*In situ* hybridization was carried out as described in Lowe et al. [88] with the following modifications: Proteinase K treatment was carried out at 10 μg/mL for 5 min at 37°C. Acetic anhydride treatment at 250 μM for 5 min at room temperature (RT) followed by a 500 μM treatment for 5 min at RT.

### Sequence retrieval

Reference sequences for the alignment were assembled from a variety of metazoans; cephalochordates [21,94], sea urchin [20], cnidarians [17,19,62,63,95,96], and sponge [17]. For a list of GenBank accession numbers, see Additional file 1: Table S1, Additional file 2: Table S2 and Additional file 3: Table S1. Sequences were aligned using ClustalW (EMBO-EBI). Trees were constructed in FigTree v1.2.3 [97] and further modified in Adobe Illustrator CS3.

### Additional software

The DNASTAR Lasergene software package was used for sequence management, genome walking, and initial alignment.

### Molecular phylogenetic analyses

All genes in this study were assigned orthology by phylogenetic analysis. Two types of analysis were carried out: (1) Bayesian analysis using MrBayes (v3.0B4) [98,99]; and (2) maximum likelihood analysis using the web-based PhyML server at Lirmm [100,101]. Alignment of the Fox (fork head box) domains was performed using ClustalW2 via the EMBL-EBI homepage [102].

### Phylogenetic analysis of *S. kowalevskii* Fox proteins

Bayesian analysis (MrBayes (v3.0B4), [98,99]) was carried out using the mixed amino acid substitution model applying four independent simultaneous Metropolis-coupled Markov Chains Monte Carlo in two independent simultaneous runs. N chains was set to 16, and the tree was calculated on a 32 CPU cluster. The likelihood model was set to gamma rates = 4. A tree was sampled every 6,000 generations for 53 million generations. The first 25% of the sampled trees were excluded via ‘burnin’ prior to consensus tree calculation. *Saccharomyces cerevisiae* Fox1 was used as an outgroup. The trees converged to a standard deviation of 0.0109. Because of the size of the dataset, the maximum likelihood analysis was performed using the Approximate Likelihood-Ratio Test (aLRT) using the SH-like model [101]. The input alignment is comprised of 200 sequences with 88 characters (see Additional file 4: Table S4).

### Phylogenetic analysis of the FoxQ2 family

The fork head box of FoxQ2 proteins from various phyla was used for this analysis, including sequences from the genome of the mollusc *Lottia gigantea* for which we identified four putative FoxQ2 genes by bidirectional blast and named *foxQ2-1* to *foxQ2-4* (see Additional file 5: Table S5). We only included proteins in our analysis which were short branching, and had an identifiable EH-I-like motif on the N-terminal or C-terminal side of the protein (see below) (Additional file 5: Table S5). The EH-I-like motif is outside the fork head box and was thus not part of the alignment. Bayesian analysis was performed using the Jones amino acid substitution model, applying four independent simultaneous Metropolis-coupled Markov Chains Monte Carlo in two independent simultaneous runs. The likelihood model was set to gamma categories = 4 and gamma rates = invgamma. A tree was sampled every 500 generations for 1 million generations. The first 25% of the sampled trees were excluded via ‘burnin’ prior to consensus tree calculation. *Nematostella vectensis foxO* was used as an outgroup. The trees converged to a standard deviation of 0.023. Maximum likelihood analysis was performed using the aLRT (SH-like model) [101]. The input alignment is comprised of 28 sequences with 136 characters (see Additional file 6: Table S6).

## Results

### Molecular phylogenetic analysis of *S. kowalevskii* Fox proteins

**Figure 1:**
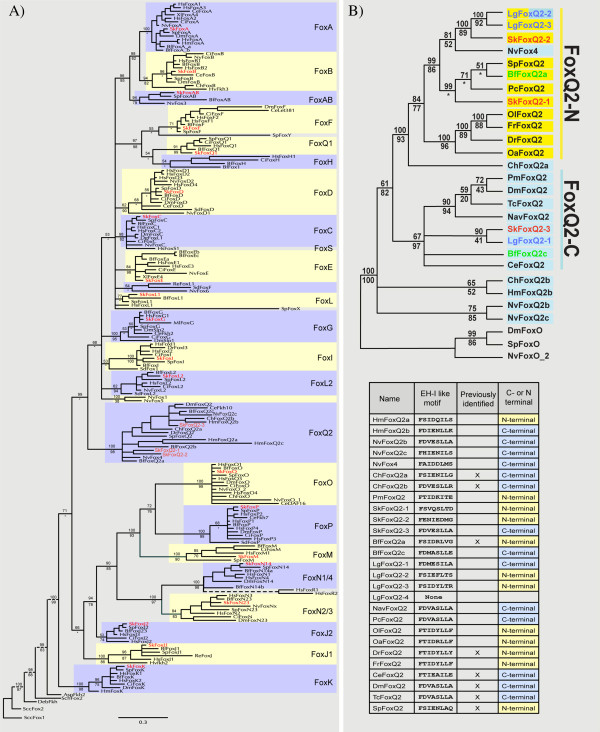
**Phylogenetic analysis. (A)** Phylogenetic analysis of *S. kowalevskii* Fox genes: The *S. kowalevskii* Fox proteins group into their predicted families with high support values. Displayed is the Bayesian tree (standard deviation = 0.0109) with Bayesian posterior probabilities values on top of each branch and maximum likelihood values underneath each branch. Stars indicate a different tree topology result from the maximum likelihood analysis which lead to no support value at that position. Branches with posterior probabilities below 50% are collapsed. For gene accession numbers, gene predictions, and alignment see Additional file 1: Table S1, Additional file 2: Table S2, Additional file 3: Table S3 and Additional file 4: Table S4. **(B)** Phylogenetic analysis of the FoxQ2 family. Phylogenetic analysis of FoxQ2 proteins containing an EH-I-like motif (see Additional file 5: Table S5) result in a tree topology supporting a duplication of the FoxQ2 family at the base of the bilaterians. Displayed is the Bayesian tree (standard deviation = 0.023) with Bayesian posterior probabilities values on top of each branch and maximum likelihood values underneath each branch. Stars indicate different tree topologies which lead to no support value at that position. Branches with posterior probabilities below 50% are condensed. Proteins with a C-terminal EH-I-like motif are highlighted in blue. Proteins with a N-terminal EH-I-like motif are highlighted in yellow. Proteins with a N-terminal and a C-terminal EH-I-like motif are highlighted in yellow and blue. For gene accession numbers, identification of the EH-like motif, and alignment see Additional file 1: Table S1, Additional file 2: Table S2, Additional file 3: Table S3, Additional file 4: Table S4, Additional file 5: Table S5 and Additional file 6: Table S6.

Comparative studies among bilaterian lineages have previously identified 24 Fox families in bilaterians (FoxA to FoxS), including the newly identified FoxAB family [19-21,103]. Two of these families are vertebrate-specific (FoxR and FoxS) [29,104], leaving 22 Fox families that were present ancestrally in bilaterians. The *S. kowalevskii* genome contains a single copy of all bilaterian Fox family members with two notable exceptions; we failed to identify a representative of FoxH, which was likely secondarily lost in hemichordates and echinoderms [20] since it is present in the mollusc *Lottia gigantea*[33]. Further, the FoxQ2 family is represented by three paralogs in *S. kowalevskii* (Figure 1). All *S. kowalevskii* Fox proteins group into their corresponding families with high bootstrap and posterior probability values, most closely related to the sea urchin or cephalochordate orthologs. Two of the sea urchin Fox proteins used in this study are long branching (*SpFoxX* and *SpFoxY*), and in our analysis they could not be assigned to a specific Fox family, supporting the result from Tu et al. [20]. The presence of a member of the FoxE family in *S. kowalevskii*, which could not be identified in the sea urchin genome, suggests a loss of FoxE somewhere in the echinoderm lineage. The FoxAB ortholog identified in *S. kowalevskii* groups reliably together with putative orthologs from sea urchin [20], cephalochordates [21], and cnidarians [19] thus making us confident that we have identified an additional member of this new family absent in vertebrates (see Figure 1a, Additional file 7: Figure S1 and Additional file 8: Table S7).

### FoxQ2 family evolution

Many animals have multiple duplications in the FoxQ2 family that have been considered species-specific duplications. Multiple FoxQ2 genes are present in the cnidarians *Nematostella vectensis* (*NvFox4*, *NvFoxQ2b*, *NvFoxQ2c*) [17,63], *Hydra magnipapillata* (*HmFoxQ2b*, *HmFoxQ2a1*, *HmFoxQ2a2*) [96], *Clytia hemisphaerica* (*CheFoxQ2a*, *CheFoxQ2b*) [19], the cephalochordate *Branchiostoma floridae* (*BfFoxQ2a*, *BfFoxQ2b*, *BfFoxQ2c*) [21], the mollusc *Lottia gigantea* (*foxQ2-1*, *foxQ2-2*, *foxQ2-3*, *foxQ2-4*), and the hemichordate *Saccoglossus kowalevskii* (*foxQ2-1*[93], *foxQ2-2*, *foxQ2-3*). Other animals like the sea urchin *Strongylocentrotus purpuratus*[20], the ascidian *Ciona intestinalis*[59], the fish *Danio rerio*[105], and the fly *Drosophila melanogaster*[37] seem to have only one FoxQ2 gene.

An ancestral subdivision of the FoxQ2 family at the base of the cnidarians has been suggested by Chevalier et al. [19], but a more detailed analysis of the evolutionary history of this family was not possible due to lack of bilaterian sequences. To address this question we included the newly available data from *S. kowalevskii* and other phyla (see Additional file 5: Table S5). In our analysis, we found that bilaterian FoxQ2 proteins clustered into two well supported groups suggesting the duplication of an ancestral FoxQ2 gene occurred before the origin of the bilaterians. However, it is not clear whether this event was in stem bilaterians or earlier, before the split of cnidarians and bilaterians: two cnidarian sequences cluster within one of the bilaterian FoxQ2 groups, whereas the others are largely unresolved or demonstrate weak support for grouping into the second bilaterian FoxQ2 group (Figure 1b). More sequence data from additional groups will be required to resolve this ambiguity.

Since the conserved region of Fox proteins is relatively short and shows little sequence variability, we found further support for our results by mapping an additional character onto the tree: the position of the EH-I-like Groucho binding domain. This domain is found in several Fox families, including the FoxQ2 family [105,106]. The EH-I-like motif is either located at the C-terminus or at the N-terminus of the FoxQ2 protein [105] outside the fork head box. Since its sequence is not included in the alignment for our phylogenetic analysis, its position can be used as an independent character to analyze the evolution of this protein family.

We identified the eight amino acid long EH-I-like Groucho binding motif [106,107] for the *S. kowalevskii* FoxQ2 family by manual sequence alignment and NCBI Protein BLAST (see Figure 1b and Additional file 5: Table S5). We found that one of the two bilaterian FoxQ2 groups contains all bilaterian FoxQ2 proteins that have the EH-I-like motif at the N-terminus of their proteins and the second group contains only bilaterian FoxQ2 proteins that have the EH-I-like motif at the C-terminus. Protein sequences from bilaterian animals with multiple FoxQ2 genes, like *Saccoglossus kowalevskii*, *Branchiostoma floridae*, and *Lottia gigantea*, were divided up into both groups.

Our data therefore show that the FoxQ2 family was already divided into two distinct groups at the base of the bilaterians. Since the data from cnidarians do not fully resolve timing of this duplication (see Discussion) we currently cannot determine whether the split of the FoxQ2 family occurred at the bilaterian base or predates the divergence of cnidarians and bilaterians.

### Clustered Fox genes

Two draft genome assemblies for *S. kowalevskii* are currently available (Baylor College of Medicin/GenBank: ACQM00000000.1 and a HudsonAlpha assembly, HudsonAlpha Institute for Biotechnology, AL (unpublished data)). Using these two drafts, four Fox genes show evidence of clustering (Additional file 9: Figure S2); *foxC*, and *foxL1* are joined on one scaffold and *foxQ2-1* and *foxQ2-3* are closely linked on a separate scaffold (see Additional file 9: Figure S2). In addition, *foxF* clusters with *foxC* and *foxL1* depending on the algorithm used (it is linked in the BCM assembly but not in the HudsonAlpha assembly). Further, we provide evidence of a link of *foxQ1* to the *foxF*, *foxC*, and *foxL1* containing scaffold by manual genome walking using unassembled trace sequences and by bidirectional blast of the scaffold ends (see Additional file 10: Table S8). However, even though no better match was found in the genome, the scaffold ends mostly contain repeats and a final assignment of *foxQ1* and *foxF* requires further characterization. The potential linkage of *foxQ1*, *foxF*, *foxC*, and *foxL1* is of particular interest since this cluster conservation may be linked to their developmental roles in mesoderm development [32,33].

### Expression analysis

**Figure 2:**
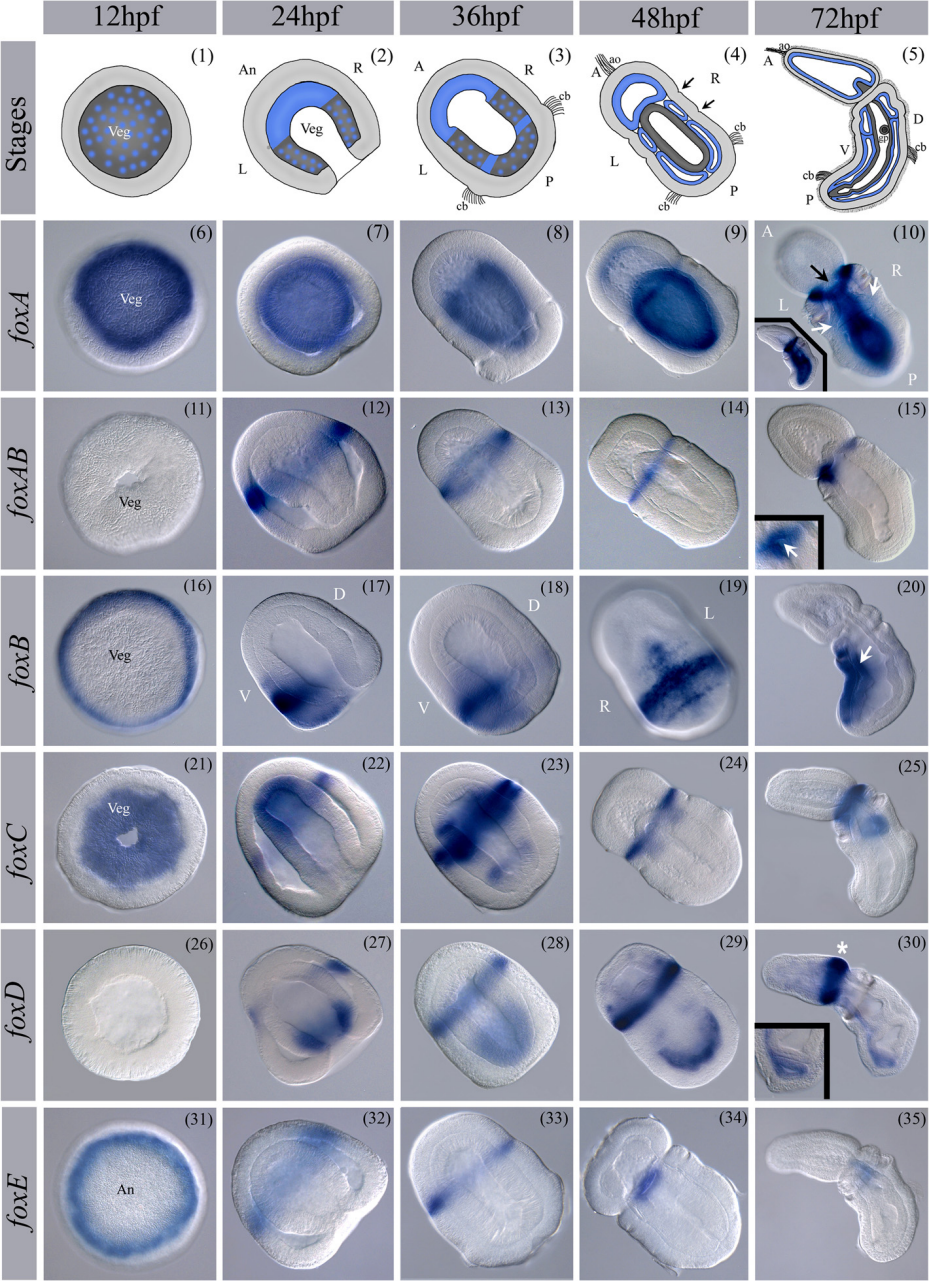
**Expression patterns of *S. kowalevskii foxA****-****E.*** Spatial expression pattern of *S. kowalevskii foxA* - *foxE*. Animals are oriented as indicated in cartoons for the corresponding stage if not otherwise specified. For a detailed description of the expression patterns see text. Panels 1 to 5; ectoderm = light gray, mesoderm = light blue, endoderm = dark gray, black arrows in panel 4 point at the forming furrows at the boundary between the proboscis and collar and collar and trunk respectively. Panels 6, 11, 16, 19, 21, and 31 show surface views. Panels 17/18 are lateral views. (10) Panel 10 shows a dorsal view, white arrows points at the forming gill pores, the black arrow points at the gap of ectodermal *foxA* expression at the dorsal collar, inlay shows lateral view. (15) Inlay in panel 15 shows ventral view on the mouth opening, white arrow points at the mouth opening. (20) White arrow in panel 20 points at the endodermal expression domain of *foxB*. (30) White star in panel 30 indicates the ectodermal expression domain of *foxD* at the base of the proboscis. The inlay shows a closeup of the posterior gut region. An: animal pole; Veg: vegetal pole; L: left; R: right; A: anterior; P: posterior; D: dorsal; V: ventral; ao: apical organ; cb: ciliated band; gp: gill pore. Brightness and contrast of pictures were adjusted when appropriate to match overall appearance of the figure.

*Saccoglossus kowalevskii* is a direct developing enteropneust [74,89-91,108]. Early cleavage is radial forming a hollow blastula (Figure 2 (1)), and gastrulation is by invagination between 16 and 30 hours post fertilization (hpf) at 20°C (Figure 2 (2)). Mesoderm forms by enterocoely from the archenteron following gastrulation at about 36 hpf (Figure 2 (3)). Embryogenesis leads to a tripartite body plan with a prosome/proboscis, a mesosome/collar, and a metasome/trunk (Figure 2 (4)), divided after 48 hpf by an anterior and posterior collar groove (Figure 2 (4) black arrows). The mouth opens on the ventral side, between the collar and proboscis, into the anterior pharynx, which leads to the posterior gut. The first gill slit forms in the posterior pharynx and perforates through the ectoderm in the anterior trunk (Figure 2 (5)), with more added sequentially during development. A more detailed description of hemichordate development can be found in [74,88-91,109,110].

#### foxA

*foxA* expression is first detected at blastula in the vegetal plate, which gives rise to the prospective endomesoderm (Figure 2 (6)). Expression is persistent in the endoderm throughout development (Figure 2 (7-10 and inlay)), but excluded dorsolaterally from the regions that give rise to the gill pores (white arrows in Figure 2 (10)). *foxA* is also expressed in the ectoderm in the anterior collar groove from approximately 48 hpf (Figure 2 (9)). In juveniles, this circumferential expression marks the most anterior collar region but is excluded from the dorsal midline (black arrow in Figure 2 (10)). A partial description of the expression of *foxA* was previously reported [73,93].

#### foxAB

*foxAB* expression was not detected at blastula stages (Figure 2 (11)). At gastrula *foxAB* is expressed in a circumferential ring in the prospective anterior ectoderm (Figure 2 (12)). The ectodermal expression persists into later stages and refines into a thin ring in the anterior collar groove (Figure 2 (12-15)). The developing mouth of the embryo perforates through this ring of expression on the ventral side (Figure 2 (15 inlay, white arrow indicates mouth)).

#### foxB

*foxB* expression is first detected at the blastula stage in a circumferential ring in the most posterior prospective ectoderm surrounding the vegetal plate (Figure 2 (16)). During gastrulation, *foxB* expression localizes asymmetrically to the posterior ventral ectoderm, flanking the ciliated band on both sides (Figure 2 (17-19, Additional file 11: Figure S3)). At 48 hpf *foxB* is expressed in the ventral endoderm in the collar region. It is further expressed ventrally in a broad stripe in the trunk ectoderm, anterior to the ciliated band and in two further narrower stripes posterior to the ciliated band and in the collar (Figure 2 (19)). This expression persists into the juvenile stage (Figure 2 (20)). At this stage, the ectodermal expression domain anterior to the ciliated band is divided into two domains (See Additional file 11: Figure S3).

#### foxC

*foxC* expression is first detected at the blastula stage in the vegetal plate (Figure 2 (21)). During gastrulation this endomesodermal expression restricts to the tip of the archenteron, which is fated to become the anterior mesoderm [111]. Circumferential ectodermal expression is also detected in the anterior of the embryo at the base of the developing proboscis (Figure 2 (22)). Upon completion of gastrulation, *foxC* is further associated with mesoderm formation and is localized to two pairs of lateral endomesoderm (Figure 2 (23)) that become the coelomic pouches of the collar and trunk [111] in a pattern very similar to *foxF* (see below). At this stage, circumferential ectodermal expression is detected in sharply delineated rings marking the anterior and posterior collar groove (Figure 2 (23)). From 48 hpf on, *foxC* expression persists in the anterior collar groove with a gap of expression at the dorsal midline, and endodermal expression is detected in the pharyngeal endoderm and is associated with gill pouch formation (Figure 2 (24/25)) (See also Additional file 11: Figure S3).

#### foxD

*foxD* expression begins during gastrulation in an anterior ectodermal circumferential ring (Figure 2 (26/27)). The circumferential ectodermal expression domain persists throughout development and localizes to the posterior proboscis at later stages (Figure 2 (27-30)). Beginning at 36 hpf*,* additional ectodermal expression is detected in scattered cells in the proboscis and continues into later stages. In the endomesoderm, *foxD* is expressed posteriorly during gastrulation and early axis elongation (Figure 2 (28/29)). Following enterocoely of the mesoderm *foxD* is expressed in the posterior-most endoderm that gives rise to the hindgut (Figure 2 (30 inlay)), and in the ventrolateral mesoderm (Figure 2 (30)).

#### foxE

*foxE* expression is first detected at early blastula in the prospective ectoderm (See Additional file 11: Figure S3). This expression refines to a ring around the animal pole before gastrulation (Figure 2 (31)). During gastrulation this circumferential expression domain persists (Figure 2 (32)) and later refines to a narrow circumferential ring localized at the base of the prosome (Figure 2 (33)). From 48 hpf on, *foxE* expression is detected in the anterior pharynx; the region that later gives rise to the stomochord (Figure 2 (34/35)) (See also Additional file 11: Figure S3).

#### foxF

**Figure 3:**
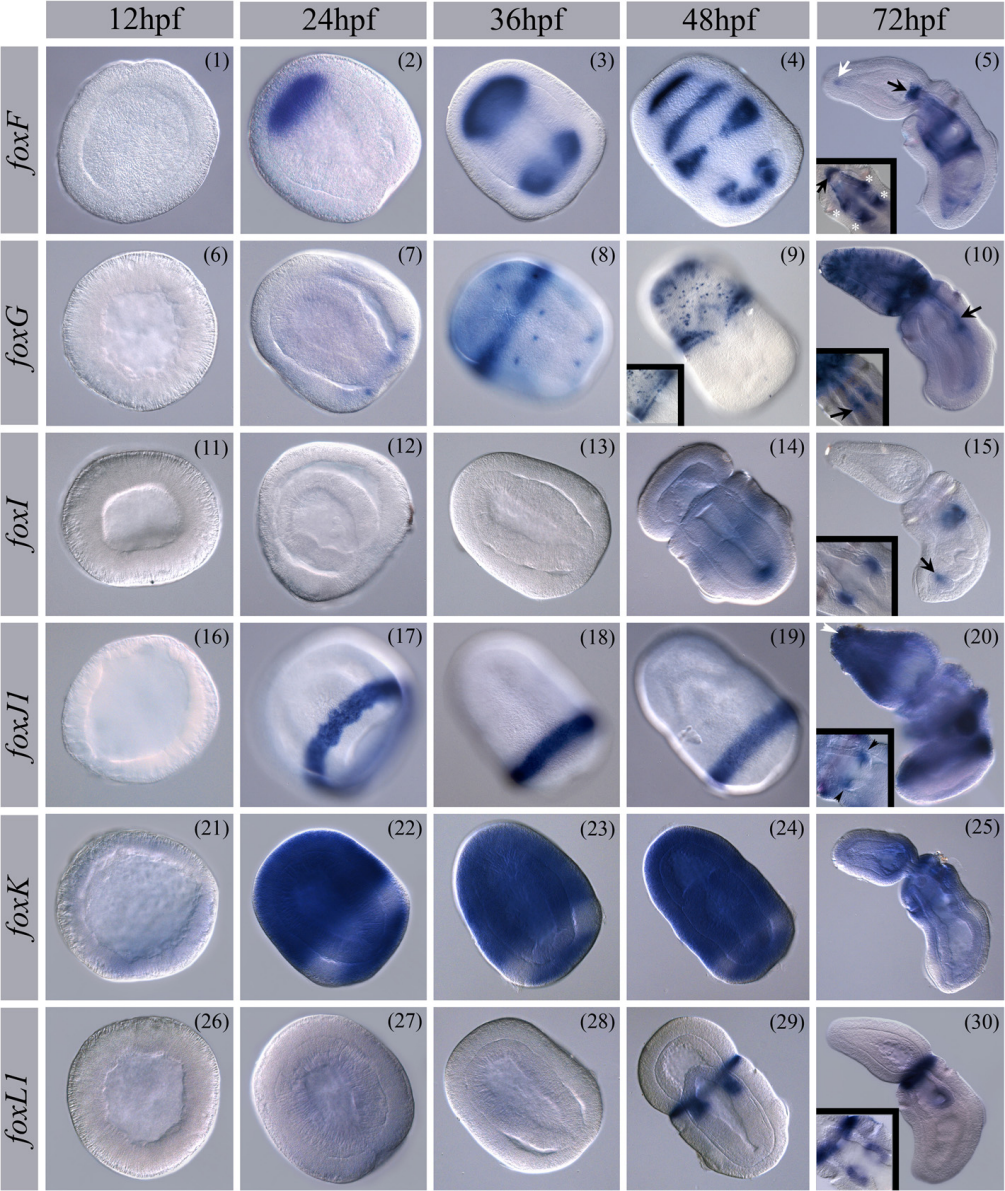
**Expression patterns of *S. kowalevskii foxF****-****L1.*** Spatial expression pattern of *S. kowalevskii foxF* - *foxL1*. Animals are oriented as indicated in cartoons of Figure 2 (1-5) for the corresponding stage if not otherwise specified. For a detailed description of the expression patterns see text. Panels 4, 8, 9, and 17-19 show surface views. (5) White arrow in panel 5 points at endodermal expression domain of *foxF* at the tip of the proboscis, black arrow points at the heart-kidney complex. Inlay shows dorsal view of the pharynx, black arrow points at the heart-kidney complex. White asterisk indicate expression in the pharyngeal mesoderm. (9) Panel 9 shows dorsal surface. Inlay shows ventral surface. (10) Arrow in panel 10 points at dorsal mesoderm. Inlay shows dorsal view of the pharynx, black arrow points at dorsal mesoderm. (15) Black arrow in panel 15 points at posterior endoderm expression of *foxI*. Inlay shows dorsal view of the forming gill pores. (20) Inlay shows dorsal view of the forming gill pores. Black arrow heads point to gill pouch endoderm. (30) Inlay shows dorsal view of the forming gill pores. An: animal pole; Veg: vegetal pole; L: left; R: right; A: anterior; P: posterior; D: dorsal; V: ventral. Brightness and contrast of pictures were adjusted when appropriate to match overall appearance of the figure.

No *foxF* expression was observed at blastula (Figure 3 (1)). Expression is first detected during gastrulation in the anterior endomesoderm that will give rise to the anterior mesoderm (Figure 3 (2)). At 36 hpf, it is expressed in the developing posterior mesoderm before enterocoely (Figure 3 (3)), and at 48 hpf in the anterior, mid, and posterior mesoderm (Figure 3 (4)) [111]. At later stages mesodermal expression is detected laterally on both sides of the pharynx (Figure 3 (5 inlay, indicated by stars)), in small patches around the posterior gut, in the most anterior tip of the embryo directly underlying the apical organ (Figure 3 (5 white arrow)) (Additional file 11: Figure S3), and the heart/kidney complex at the tip of the developing stomocord (Figure 3 (5 and inlay, black arrow)). Mesoderm expressing *foxF* during juvenile development always lines the endoderm (visceral mesoderm) except for the expression at the tip of the proboscis mesoderm (See also Additional file 11: Figure S3).

#### foxG

No expression of *foxG* was detected during the blastula stage (Figure 3 (6)). Expression is first detected in the posterior ectoderm during gastrulation in a few single cells (Figure 3 (7)). From 36 hpf on, it is expressed in a circumferential ring in the anterior third of the embryo and in scattered cells throughout the ectoderm (Figure 3 (8)). At 48 hpf, the ring of expression refines into two separate rings of cells at the base of the proboscis but does not extend to the dorsal midline (Figure 3 (9 dorsal view, inlay shows ventral view)). Single cell expression is detected throughout the proboscis ectoderm with a greater density at the proboscis tip. In juveniles, *foxG* is detected in the dorsal mesoderm overlaying the pharynx and gut (Figure 3 (10, black arrow, inlay shows dorsal view)). A partial description of *foxG* expression was previously published [71,73].

#### foxI

Localized *foxI* expression was not detected at early developmental stages (Figure 3 (11-13)) and is first detected at 48 hpf in the most posterior endoderm and in an ectodermal circumferential ring of expression anterior to the ciliated band (Figure 3 (14)). At juvenile stages, expression is detected in a small domain in the center of the gill pouch endoderm (Figure 3 (15, inlay dorsal view)) and in the posterior gut (Figure 3 (15, black arrow)) (See also Additional file 11: Figure S3).

#### foxJ1

No *foxJ1* expression was detected in blastulae (Figure 3 (16)). *foxJ1* is expressed from gastrula in an ectodermal ring marking the forming ciliated band (Figure 3 (17)). The expression in the ciliated band is persistent in all later stages examined (Figure 3 (17-20)). In juveniles, additional broad ectodermal expression is detected in the proboscis and the anterior trunk (Figure 3 (20)) including the apical organ (Figure 3 (20 white arrow head)). Endodermal expression is detected in the gill pouches (Figure 3 (20 and inlay, black arrow heads)) (see also Additional file 11: Figure S3).

#### foxK

Only weak staining of *foxK* was observed at the blastula stage (Figure 3 (21)). An almost ubiquitous expression of *foxK* is detected at the late gastrula stage throughout the ectoderm but excluded from the ciliated band (Figure 3 (22)). This expression persists throughout development (Figure 3 (22-24)) until it is restricted to the collar ectoderm in juvenile embryos (Figure 3 (25)). Endoderm and mesoderm expression was not examined in early developmental stages, and no mesoderm or endoderm staining was observed in juveniles.

#### foxL1

No *foxL1* expression was detected in early development (Figure 3 (26-28)). *foxL1* expression is first detected at 48 hpf in a circumferential ectodermal band at the base of the proboscis and in the endoderm of the out-pocketing gill pouches and in all subsequent stages examined (Figure 3 (29/30 inlay shows dorsal view)).

#### foxN1/4

**Figure 4:**
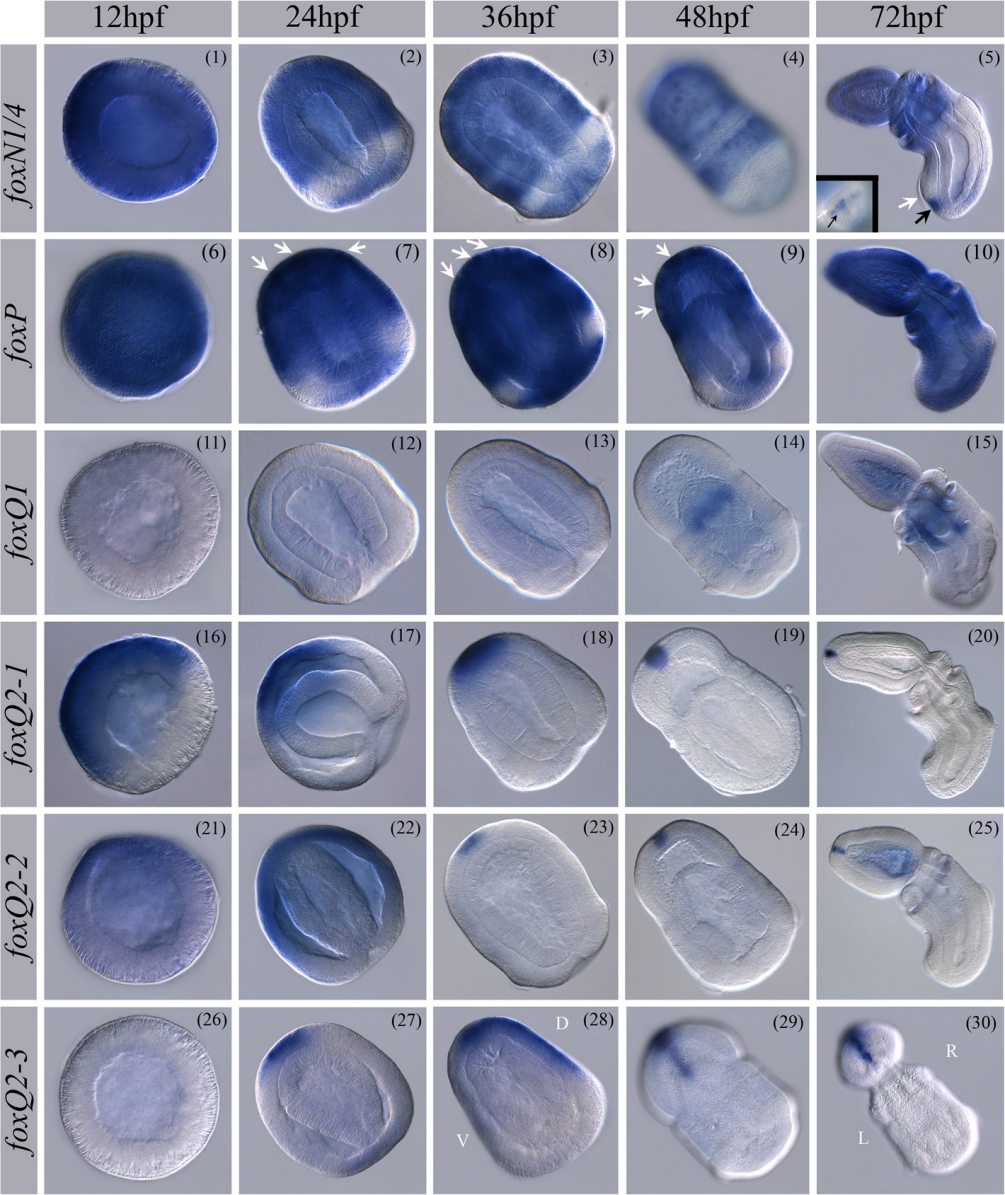
**Expression patterns of *S. kowalevskii foxN1/4****-****Q2****-****3.*** Spatial expression pattern of *S. kowalevskii foxN1/4* - *foxQ2-3*. Animals are oriented as indicated in cartoons of Figure 2 (1-5) for the corresponding stage if not differently specified. For a detailed description of the expression patterns see text. Panels 4, 6, 29, and 30 show surface views. Panels 14/15 and 23-25 show light stained embryos. For longer stained embryos see Additional file 11: Figure S3. (5) Black arrow points at expression domain of *foxN1/4* in the ventral ectoderm at the posterior tip of the trunk, white arrow points at the ciliated band. Inlay shows ventral view of the posterior tip of the trunk, black arrow points at expression domain of *foxN1/4*. (7-9) White arrows point to cells with high levels of *foxP* expression in the proboscis ectoderm. An: animal pole; Veg: vegetal pole; L: left; R: right; A: anterior; P: posterior; D: dorsal; V: ventral. Brightness and contrast of pictures were adjusted when appropriate to match overall appearance of the figure.

Ubiquitous expression of *foxN1/4* is observed at blastula (Figure 4 (1)). At 24 hpf and 36 hpf *foxN1/4* is expressed throughout the ectoderm except the ciliated band. Scattered cells nested within the broad domains of ectodermal expression show higher levels of expression compared to the general ectodermal staining (Figure 4 (2/3)) (See also Additional file 11: Figure S3). At 48 hpf, *foxN1/4* expression continues in the anterior ectoderm but only faint staining was detected in the central and posterior collar region (Figure 4 (4)). At juvenile stages, *foxN1/4* expression is restricted to the proboscis and collar, and to a thin row of cells (black arrow) posterior to the ciliated band (white arrow) (Figure 4 (5, inlay shows ventral view of the trunk tip)). Endoderm and mesoderm expression was not examined. (For surface views see Additional file 11: Figure S3).

#### foxP

Ubiquitous ectodermal *foxP* expression is detected at blastula (Figure 4 (6)). From gastrulation, expression is detected in the entire ectoderm except the ciliated band (Figure 4 (7)), and stronger expression is detected in single cells scattered throughout the anterior (Figure 4 (7-9 white arrows)). From 48 hpf, expression is detected in single cells throughout the proboscis ectoderm (Figure 4 (9)), this expression persists into the juvenile stage (Figure 4 (10)). Endoderm and mesoderm expression was not examined.

#### foxQ1

No *foxQ1* expression was detected up to 36 hpf of development (Figure 4 (11-13)). Localized expression is first detected at 48 hpf in the anterior endoderm at the position where the gill pouches are forming (Figure 4 (14/15)). At juvenile stage, expression continues in the anterior pharynx (Figure 4 (15)). If embryos are stained long expression in the overlying ectoderm of the trunk and collar, with the exclusion of the ciliated band, is also observed (Additional file 11: Figure S3).

#### foxQ2

All three FoxQ2 paralogs (*foxQ2-1*, *foxQ2-2*, and *foxQ2-3*) share an apical expression pattern, but each exhibits unique expression characteristics (Figure 4 (16-30)). *foxQ2-1* is expressed in the animal hemisphere at blastula (Figure 4 (16)). During development from gastrula to juvenile, expression becomes refined to the most apical cells marking the site of the ciliated apical organ (Figure 4 (17-20)). A partial description of the expression pattern of *foxQ2-1* was reported previously by Darras et al. [93]. *foxQ2-2* expression also displays an ectodermal apical domain similar to *foxQ2-1* (Figure 4 (21-25))*.* However, in contrast to *foxQ2-1*, *foxQ2-2* shows ubiquitous ectodermal expression throughout the embryo if stained for longer period of time (Additional file 11: Figure S3). Localized *foxQ2-3* expression is first detected at the gastrula stage in the apical territory (Figure 4 (27)). At 36 hpf, expression in the apical domain extends in a stripe along the dorsal midline of the embryo starting from the most apical part of the embryo and extending posteriorly, covering approximately two-thirds of the embryo (Figure 4 (28)). At later stages, this dorsal stripe becomes restricted anteriorly to the dorsal proboscis midline (Figure 4 (29/30)).

#### foxJ2/3*,* foxL2*,* foxM*,* foxN2/3*, and* foxO

For five genes *foxJ2/3*, *foxL2*, *foxM*, *foxN2/3*, and *foxO*, reliable localization was not detectable by *in situ* hybridization.

**Figure 5:**
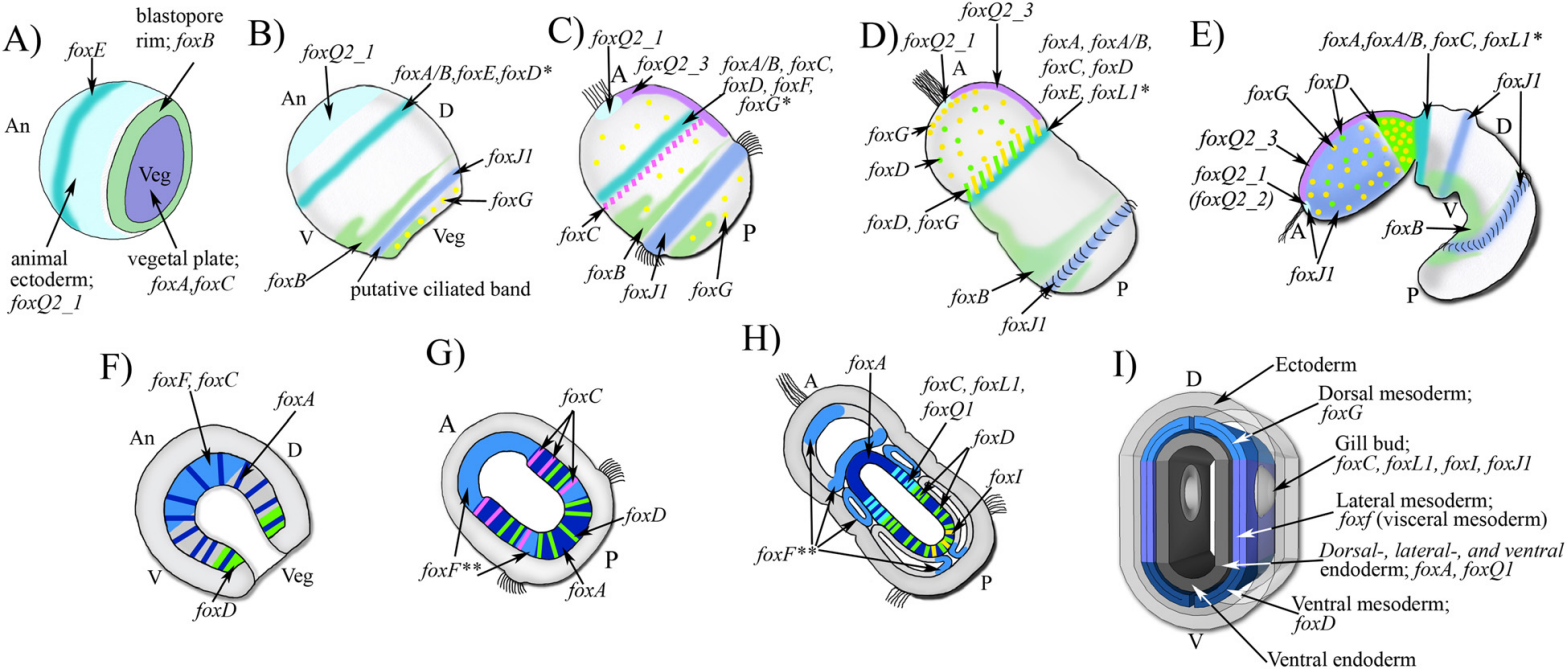
**Expression summary. (A-I)** Expression summary of all *S. kowalevskii* Fox genes with clear localized expression patterns. **(A-E)** Blastula -, Gastrula -, 36 h embryo -, 48 h embryo -, 72 h embryo - surface view. **(F-H)** Gastrula -, 36 h embryo -, 48 h embryo - cross section. **(I)** Cross section of the gill pore area of a 72-h-old embryo. For details see text. *Potential co-expression is inferred from single gene expression analysis. No double *in situ* hybridization was performed. **The expression of *foxF* is very dynamic and only a more detailed analysis will be able to show all expression domains at any given developmental time point. An: animal, Veg: vegetal, A: anterior, P: posterior, D: dorsal, V: ventral.

For a comprehensive summary of all localized *S. kowalevskii* Fox genes see Figure 5.

## Discussion

### Fox gene complement of *Saccoglossus kowalevskii*

The Fox gene family is a large group of transcription factors with at least 24 family members. Our characterization of the Fox gene complement in *S. kowalevskii* revealed 21 of the 22 Fox family members predicted for the ancestral bilaterian. This includes a member of the FoxE family, which is absent in sea urchins, suggesting a loss of FoxE somewhere in the echinoderm lineage. *S. kowalevskii* only lacks a representative of the FoxH family, a gene that is absent from the genome of the sea urchin *S. purpuratus* and likely secondarily lost in the ambulacrarian lineage (See Results and Figure 1). We further identified three FoxQ2 genes in *S. kowalevskii*. The FoxQ2 family likely arose in the lineage leading to the common cnidarian bilaterian ancestor since no FoxQ2 homologs have been described in sponges [17,33,66] or ctenophores [112]. From our phylogenetic analysis, we have identified a subdivision of the FoxQ2 family into two distinct groups that occurred at least at the base of the bilaterians, but possibly earlier (Figure 1b and Results). This interpretation is supported by the position of the EH-I binding motif, which is located either on the C-terminal or N-terminal side of the bilaterian FoxQ2 proteins. Each of the two bilaterian FoxQ2 groups is characterized by either the C-or N-terminal motif. All cnidarian FoxQ2 genes with one exception are characterized by a C-terminal EH-I motif. Two of these group with the bilaterian FoxQ2 group characterized by the EH-I motif at the N-terminus. It is therefore likely that the position of the EH-I motif was ancestrally at the C-terminus and was relocated to the N-terminal domain in one copy of the bilaterian FoxQ2 paralogs at the base of the bilaterians. The presence of an N-terminal motif in the Hydra gene *HmFoxQ2a*, which is long branching and could not be placed in our phylogenetic analysis, appears to be secondarily derived.

The question remains whether a duplication in the FoxQ2 family occurred at the bilaterian base or before the bilaterian/cnidarian split. The latter scenario is the most parsimonious based on our analysis, but more data from cnidarians are needed to answer this question definitively: most available cnidarian sequences currently group outside all bilaterian FoxQ2 sequences with only a few inside the N-terminal clade making this a difficult issue to resolve.

### Conserved expression domains of Fox genes

By comparing the results of our expression analyses (Figures 2, 3, 4, and 5) to the current literature we highlight the expression of several Fox genes that may represent ancestral deuterostome or bilaterian developmental roles.

### Apical ectoderm patterning and tissue specification (*foxQ2*)

FoxQ2 gene expression has been characterized during the development of a phylogenetically wide range of bilaterian and non-bilaterian animals (For references see Result section for FoxQ2 and Table 1). In bilaterians FoxQ2 genes are commonly expressed at the animal pole early in development and quickly restricted to a narrow region at the most apical region of the embryo [20,21,37,61]. Since this pattern is observed in many bilaterians it is proposed that FoxQ2 has an evolutionarily conserved role in apical ectoderm patterning [45,61]. Functional studies in bilaterians further imply that the restriction of FoxQ2 to the apical tip of the ectoderm is mediated by Wnt/β-catenin signaling [45,93,113] and imply that this regulation is also evolutionarily conserved.

**Table 1:** Literature summary

**Gene name**	**Observed expression in *S. kowalevskii***	**Related gene expression domain in other organisms**
**foxA**	**Late blastula:**	**Protostomes:**
	Vegetal plate.	Endoderm specification [103,114-116].
	**48 hpf:**	**Hemichordates:**
	Anterior collar groove ectoderm; entire endoderm.	Vegetal plate, endoderm/foregut [117].
	**Juvenile stage:**	**Echinoderms:**
	Entire endoderm with the exception of the dorsolateral outpocketing gill pores; most anterior collar ectoderm with a gap in expression on the dorsal midline	Presumptive ventral ectoderm [118]; Endomesoderm specification [44,46].
		**Vertebrates:**
		Gastrulation, endoderm patterning, notochord formation [18,119].
		**Urochordates:**
		Gastrulation, axis formation [120].
		**Cnidarians:**
		Preendodermal plate, pharynx [62,121].
**foxAB**	**24 hpf:**	**Protostomes:**
	Circumferential ectodermal ring which localizes to the anterior collar groove during later development.	In the bryozoans larval ciliated cleft and apical ectodermal territory [103].
	**Juvenile stage:**	FoxAB orthologues are further identified in echinoderms [20], cephalochordates [21], cnidarians [19] but expression is unknown.
	The developing mouth of the embryo breaks through at the ventral side of the embryo inside the *foxAB* expression domain	
**foxB**	**Late blastula:**	**Sea urchins:**
	Ring pattern surrounding the vegetal plate.	Ingressing primary mesenchyme cells; asymmetrically expressed along the oral-aboral axis in the oral ectoderm and endoderm [122-124].
	**48 hpf:**	**Cnidarians:**
	Ventral ectoderm anterior and posterior to the ciliated band.	Around the site of gastrulation, larval endoderm region, in the statocyst, gonad and tentacle bulb of the medusa [19,63].
	**Juvenile stage:**	**Chordates:**
	Ventral ectoderm and ventral pharyngeal endoderm.	Dorsal ectoderm, mesoderm, brain: *Xenopus*[125-127], ascidians [59], *B. floridae*[128].
		**Protostomes:**
		Ventral ectoderm [40,129]; ventral nervous system [35].
**foxC**	**Late blastula:**	**Chordates:**
	Vegetal plate.	Pharyngeal mesoderm patterning [31,32,34,130-132]. Pharyngeal endoderm [34].
	**24 hpf:**	**Vertebrates:**
	Anterior endomesoderm.	Ventral and lateral mesoderm, lateral border of neuroectoderm, eye, pronephros [133,134] (*Xenopus*, reviewed in [122])
	**48 hpf:**	**Cnidarians:**
	Circumferential ectodermal expression in the anterior and posterior collar groove; endomesodermally at the positions of mesoderm formation.	Presumptive endoderm and mesenteries [63].
	**Juvenile stage:**	
	Endodermal expression in the pharyngeal endoderm associated with gill pouch formation.	
**foxD**	**24 hpf:**	**Chordates:**
	Anterior ectodermal circumferential ring.	Notochord [52,57]
	**36 hpf:**	Diencephalon: reviewed in [61]
		Neural crest [52,76,135-142] (Reviewed in [52,122]). Maintenance of dorsolateral mesoderm (*Xenopus)*[130,143] (reviewed in [122]). Hindgut [135,144].
		**Urochordates:**
	Anterior ectodermal circumferential ring.	Dorsal anterior ectoderm [57].
	Posterior endomesoderm.	
	**48 hpf:**	**Cephalochordates:**
	Anterior ectodermal circumferential ring.	Anterior neural plate, the anterior somites, the neural tube, and later in the cerebral vesicle, hindgut [52,122,145-149].
	Ectodermal single cells throughout the proboscis.	
	**Juvenile stage:**	**Sea urchins:**
	Posterior-most endoderm forming the hindgut; ventrolateral mesoderm.	Hindgut [20].
		**Protostomes:**
		Dorso-ventral circumferential cell migration and axon projection; ventral mediolateral muscles, intestinal precursor cells (*C. elegans)*[150-153]. Ventral nervous system (*Drosophila*) [19].
		**Cnidarians:**
		Aboral third of the embryo, tentacle buds [63].
		**Interaction with BMP/TGF**-**beta pathway:**
		[36,40,55,130,143,150,151,154],[155].
**foxE**	**12 hpf:**	**Vertebrates:**
	Ectodermal ring around the animal pole.	Thyroid (Endoderm) [156]
	**48 hpf:**	**Urochordates:**
	Ectodermal circumferential ring localized at the base of the prosome (48 hpf).	Endostyle (Endoderm) [58] Reviewed in [157].
	**Juvenile stage:**	**Cephalochordates:**
	Anterior-dorsal pharynx endoderm including the stomochord.	Club shaped gland (Endoderm) [51,158].
**foxF**	**24 hpf:**	**Chordates:**
	Anterior endomesoderm.	Mesoderm patterning [19,31,32,34,159-161].
	**36 hpf:**	Gill slit formation in chordates: reviewed in [34].
	Developing lateral and posterior mesoderm.	Neural plate border, cephalic neural crest, pronephros: *Xenopus*, reviewed in [122].
	**48 hpf:**	**Protostomes:**
	Posterior, central, and anterior mesoderm.	Mesoderm [36].
	**Juvenile stage:**	
	Mesoderm surrounding the pharynx; mesoderm around the posterior gut; a mesodermal spot underneath the site of apical organ formation; heart-kidney complex; the pharyngeal mesoderm with accumulation of *foxF* expressing cells anterior and posterior to the forming gill pores. (Expression is absent at the position where the evaginating gill pore endoderm connects to the ectoderm.)	
**foxG**	**24 hpf:**	**Vertebrates:**
	Few single cells in the ectoderm.	Telencephalon (reviewed [50]) [162-164].
	**36 hpf:**	**Cephalochordates:**
	Strong ectodermal circumferential ring in the anterior third of the embryo.	Scattered cells surrounding the cerebral vesicle and inside the cerebral vesicle [50]
	**48 hpf:**	
	Two ectodermal rings with a gap of expression on the dorsal midline; single cell expression throughout the proboscis with a density of single cells at the proboscis tip.	
	**Juvenile stage:**	
	Additionally to 48 hpf expression: dorsal mesoderm overlaying the pharynx and gut.	
**foxH**	Not present in the *S. kowalevskii* genome	
**foxI**	**48 hpf:**	**Vertebrates:**
	Most posterior endoderm; weak ectodermal circumferential ring of anterior to the ciliated band.	Craniofacial development [122,127,165].
	**Juvenile stage:**	**Sea urchins:**
	Small domain in the center of the outpocketing gill pouch endoderm; posterior gut.	Larval hindgut with high expression levels on the aboral side [20].
**foxJ1**	**24 hpf:**	**Vertebrates:**
	Ectodermal in the ciliated band domain.	Master regulator in the formation of motile cilia [60,166-172].
		Mediates left-right asymmetry [166,173-177].
	**Juvenile stage:**	**Echinoderms:**
	Ectodermal in the ciliated band, anterior proboscis ectoderm including the apical organ; gill pores; posterior collar.	Oral side of the apical plate [20]; Larval ciliary band [48].
		**Protostomes:**
		Ampullary cells, crescent cells, and prototroch (*Platynereis*) [113]
		**Cnidarians:**
		[17,178]
		**Yeast:**[178]
		(Hcm1p) is involved in spindle pole body formation [179].
		FoxJ1 orthologues are further identified in Choanoflagellates [180], sponges [66], other deuterostomes and protostomes [33], but expression and function is not known.
**foxJ2/3**	Not determined.	
**foxK**	**>12 hpf:**	**Vertebrates:**
	Ubiquitous throughout the ectoderm with the exception of the ciliated band.	Dorsal midline, lateral cephalic neural crest, brain, eye, lateral muscle precursors (*Xenopus*) [122,168].
	**Juvenile stage:**	
	Collar ectoderm.	
**foxL1**	**48 hpf:**	**Chordates:**
	Circumferential ectodermal band in the anterior collar groove and in the endoderm of the outpocketing gill pores. This expression persists until the one gill slit stage.	Pharyngeal mesoderm patterning [31,32,34].
		Gill slit endoderm (*Scyliorhinus canicula)*[34].
**foxL2**	Not determined.	
**foxM**	Not determined.	
**foxN1/4**	**12 hpf:**	**Mammals:**
	Ubiquitous expression in the ectoderm.	*foxN1* is essential for proper immune response in mice [181]. Downstream target of the Wnt-pathway [182].
		*foxN4* is involved in specifying amacrine and horizontal cells in the retina and is upstream of the bHLH gene Math3, NeuroD1, and Prox1 [113,183,184] (reviewed in [185]).
		It is necessary for the development of V2a and V2b interneurons in the spinal cord using lateral inhibition via the Delta-Notch pathway by activating the transcription of Delta4 and the bHLH gene Mash-1 [186,187].
	**24 hpf:**	
	Ubiquitous ectodermal expression with the exception of the ciliated band.	
	**48 hpf:**	
	Entire ectoderm with the exception of the central and posterior collar region and the ciliated band. Nested inside these expression domains are single cells that show a high levels of expression.	
	**Juvenile stage:**	
	Proboscis and collar region; thin row of cells posterior to the ciliated band.	
**foxN2/3**	Not determined	
**foxO**	Not determined	
**foxP**	**24 hpf:**	**Vertebrates:**
	Entire ectoderm with the exception of the ciliated band.	Basal ganglia, cerebral cortex, cerebellum, and thalamus, hippocampus [127,188-190]. In *Medaka foxP1* expression indicates a role during striatum projection neuron development [191] and basal ganglia development of the developing and central nervous system [192].
	>**36 hpf:**	Mutations in human *foxP2* gene lead to severe language disorders [193-195] (reviewed in [127,196]).
	High levels of expression in single cells in the anterior ectoderm of the embryo; weak endodermal expression.	**Urochordate:**
		Developing brain [59].
		**Echinoderms:**
		Fore- and mid-gut of the larva [20].
		**Protostomes:**
		In *Drosophila* the two splice variants of foxP (*fd85Ea* and *fd85Eb*) are expressed in the developing CNS [37,197].
		A FoxP ortholog is also found in other ecdysozoans, cnidarians, and sponges [33,66], but expression patterns are not yet reported.
**foxQ1**	**48 hpf:**	**Vertebrates:**
	Anterior endoderm at the position where the gill pores are forming; faint circumferential ectodermal ring at the position where the gill pores are forming.	Prospective pharynx, pharyngeal pouches [60],
	**Juvenile stage:**	**Cephalochordates:**
	Anterior pharynx.	Endostyle [54].
		**Urochordates:**
		Pharyngeal gills, endostyle [58].
		**Protostomes:**
		Pharyngeal endoderm [32].
**foxQ2-1**	**12 hpf:**	**Deuterostomes:**
	Animal hemisphere.	Apical ectoderm [20,21,53,198].
	>**24 hpf:**	**In protostomes:**
	During the development from gastrula to juvenile, the ectodermal expression becomes refined to cells forming the apical organ.	Anterior tip of the embryo at the blastoderm stage, pharyngeal structures and the brain hemispheres (*Drosophila*) [37]. Apical ectoderm (*Platynereis*) [113]
		**Cnidarians:**
		Apical tuft [19,178].
		**Linke to Wnt/β**-**catenin signaling:**
		[19,45,64,73].
**foxQ2-2**	*foxQ2-2* expression resembles the expression of *foxQ2-1* with additional ubiquitous expression throughout the embryo ectoderm.	
**foxQ2-3**	**24 hpf:**	
	Apical ectodermal territory.	
	**36 hpf:**	
	Ectodermal stripe along the dorsal axis of the embryo starting from the most apical part of the embryo and extending posterior, covering approximately two-thirds of the embryo.	
	**Juvenile stage:**	
	Ectodermal stripe along the dorsal proboscis midline.	

Recent functional studies outside bilaterians also demonstrate a regulatory interaction between Wnt/β-catenin signaling and FoxQ2 expression [19,65]. In the cnidarian *Clytia hemisphaerica*, *CheFoxQ2a* is expressed opposite the site of gastrulation, which is marked by nuclear β-catenin and the expression of multiple Wnt genes [19,64]. Knock down of *CheWnt3a* prevents the restriction of *CheFoxQ2a* to the most aboral ectodermal tip of the embryo [64,65] demonstrating a regulatory interaction of *CheFoxQ2a* with the canonical Wnt signaling pathway during axial patterning and supporting the hypothesis of an evolutionarily conserved interaction of FoxQ2 and Wnt/β-catenin signaling predating the split between cnidarians and bilaterians.

In addition to the role of FoxQ2 in apical ectoderm patterning it is also proposed to be involved in apical organ formation, a neural rich structure located at the apical tip of many bilaterian and non-bilaterian larvae [199] generally comprised of sensory cells, neurons, and long motile cilia that form the apical tuft. Even though a homology of the apical organ among metazoans is favored [113,178,200-203] there remain dissenting views [61,204,205].

**Figure 6:**
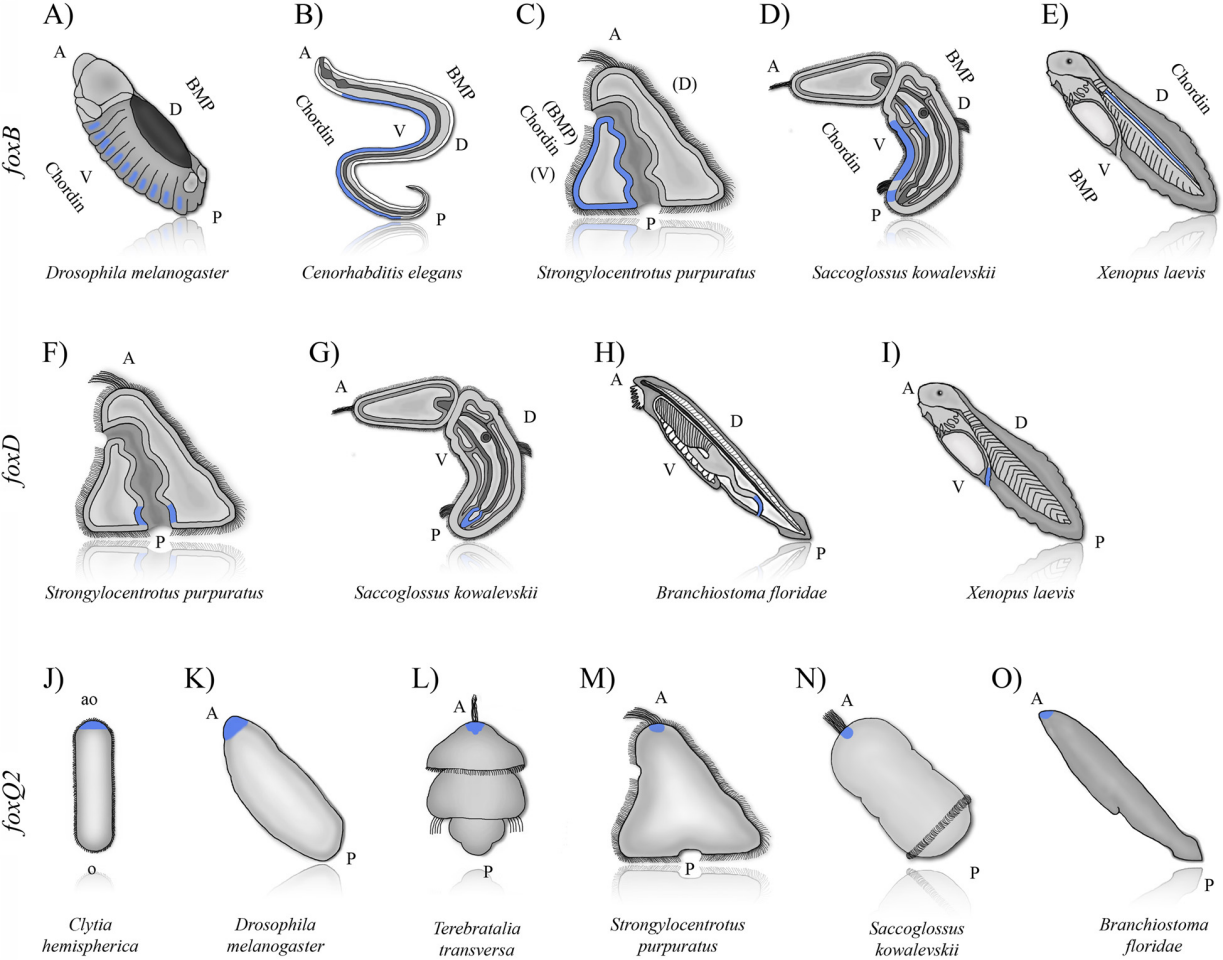
**Examples of conserved Fox gene expression domains. (A**-**E)** FoxB expression (blue) in multiple species. The expression of FoxB seems to be correlated to the expression of *chordin*, a BMP inhibitor. The endodermal gut expression domain seems to be unique to echinoderms and hemichordates. **(F**-**I)** FoxD expression (blue) in the hindgut of different species. The conservation of the expression in the hindgut across deuterostomes indicates that it was likely already present in the hindgut of the deuterostome ancestor. **(J**-**O)** Ectodermal expression domains of FoxQ2 (blue) in multiple species across phyla. Expression across bilateria suggest a conserved role in apical (apical neural) patterning. Expression at the aboral side in cnidarians suggests a deep evolutionary origin for this expression in patterning terminal neural structures. For literature summary see Table 1 and for additional discussion see Additional file 12. A: anterior, P: posterior, D: dorsal, V: ventral, oa: aboral, o: oral.

In both bilaterian and cnidarian larvae with an apical organ/tuft, FoxQ2 expression coincides with the position of this structure (see Figure 6) [19,178]. Functional studies specifically investigating the role of FoxQ2 in apical organ formation have been demonstrated in sea urchins and cnidarians [178,206]. Loss of function in sea urchins compromises the development of the long apical tuft cilia [206]. In the cnidarian *N. vectensis* expression of one out of four FoxQ2 genes has been reported [178]. At the planula stage it is expressed around the apical organ/apical tuft. Knock down experiments show that *NvFoxQ2a* is involved in the determination of the size of the apical organ/apical tuft [178].

Expression data and functional studies further suggest an evolutionarily conserved core regulatory network for apical organ formation including an apical Six3 domain, with FoxQ2 and FoxJ1 expressed in the apical organ domain in a Wnt negative territory [113,178].

In *S. kowalevskii* expression of all three FoxQ2 genes is localized at the apical tip of the ectoderm during development. *foxQ2-1*, which shows the most resemblance in expression to other reported FoxQ2 expression patterns, begins broadly in the animal hemisphere at early blastula stage. At later developmental stages, expression becomes restricted to the apical tip at the site of the apical organ (Figure 4 (16-20)) similar to other bilaterian groups. *foxQ2-1* is co-expressed with the motile cilia marker *foxJ1* in the apical organ (see below and Result section for *foxJ1*), but unlike other groups *foxQ2-1* is co-expressed with *six3*[71], and *foxJ1* has other broad expression domains not tightly localized to the apical organ. Experimental manipulations of Wnt/β-catenin signaling in *S. kowalevskii* provide support for a Wnt/β-catenin dependent localization of *foxQ2-1* to the apical ectoderm [93], but a role of *foxQ2-1* in apical tuft patterning will still need to be directly tested by experimental knockdown. Our analysis of *S. kowalevskii* FoxQ2 genes therefore supports the hypothesis that they play an evolutionarily conserved role in patterning an anterior ectodermal territory in bilaterians and that anterior restriction of FoxQ2 is mediated by Wnt/β-catenin signaling.

### Formation of motile cilia (*foxJ1*)

In contrast to primary cilia which have a 9 + 0 arrangement of their microtubules and only sometimes possess dynein arms (Nodal cilia), motile cilia show a 9 + 2 arrangement of their microtubules, are generally longer than primary cilia, and possess outer dynein arms mediating motility (reviewed in [207,208]). In vertebrates, motile cilia are mainly found in tissues where fluid movement is necessary, like lung epithelia or in the embryo node where it is involved in mediating left-right asymmetry [166,173-176,209]. Expression and functional studies in vertebrates imply that FoxJ1 is a master regulator of the formation of these motile cilia [60,166-169,210] (reviewed in [209]). A conserved role of FoxJ1 in motile cilia formation is supported from expression patterns outside chordates. *foxJ1* expression has been described during sea urchin development where it is expressed in the most apical ectoderm marking the apical organ/apical tuft, and the ciliated bands [20,48], the sea star *Patiria miniata* where *foxJ1* is expressed throughout the ectoderm at the blastula stage and the ciliated bands at the larval stage [48], the planarian *Schmidtea mediterranea* where *Smed-foxJ1-2* and *Smed-foxJ1-2* are expressed along the AP axis in a ventral stripe of presumptive motile ciliated sensory cells [210], the annelid *Platynereis dumerilii* where *foxJ1* is expressed in the apical plate and the ciliated bands, and the cnidarian *Nematostella vectensis* where *NvFoxJ1* is marking the apical organ territory [178].

In *S. kowalevskii foxJ1* is also expressed in the region forming motile cilia; the ciliated band (Figure 3 (17-20)) and the gill pore endoderm in juveniles (Figure 3 (20)). In juveniles it is further expressed broadly in the anterior ectoderm which incorporates the territory of the apical organ. Our data therefore support the hypothesis that FoxJ1 has an evolutionarily conserved function in motile cilia formation that predates the bilaterian-cnidarian split. Further, our data support an evolutionarily conserved function of FoxJ1 in cilia formation in the apical organ despite the rather broad anterior ectodermal expression in *S. kowalevskii* when compared to the tightly localized expression of FoxJ1 in the developing apical organs of other metazoans. However, since the expression of FoxJ1 is correlated with all motile ciliated cells with a 9 + 2 arrangement of their microtubules, FoxJ1 expression provides limited insights into the homology of this structure.

### Anterior endoderm and mesoderm patterning (*foxQ1, foxF, foxC, foxL1*)

The third germ layer of bilaterians, mesoderm, likely evolved at the base of the bilaterians and gives rise to many essential components of the bilaterian body. The emergence and evolution of mesoderm is therefore of special interest to understand bilaterian body plan evolution. Several Fox genes, namely FoxF, FoxC, and FoxL1, are proposed to have evolutionarily conserved functions in patterning distinct mesodermal populations [31,32,211]. Ancestral linkage of these three Fox genes, along with FoxQ1 in stem bilaterians, has been proposed to be related to their conserved developmental functions [31,32,34,159,211]. The analysis from Shimeld et al. [32] suggests that FoxC and FoxL1 play a conserved role in somatic mesoderm formation (mesoderm lining of ectoderm), FoxF in visceral mesoderm formation (mesoderm lining of endoderm), and FoxQ1 in anterior gut endoderm formation.

Our genomic analysis of *foxQ1*, *foxF*, *foxC*, and *foxL1* in *S. kowalevskii* shows a possible link of these four genes in the *S .kowalevskii* genome (see Additional file 9: Figure S2). Expression studies show that *foxL1* is only expressed in the endoderm in *S. kowalevskii* but not in the mesoderm*.* Expression of FoxL1 in the mesoderm of protostomes and other deuterostomes suggests this likely represents secondary loss in hemichordates. Expression of *foxQ1* in the foregut of *S. kowalevskii* is consistent with the proposed conserved role in anterior endoderm formation*.* Early expression of *foxF* in the forming mesoderm suggests a conserved role in mesoderm patterning, but the division of early mesoderm into somatic and visceral territories remains to be characterized in *S. kowalevskii*. However*,* in later stages (juveniles) *foxF* marks the mesoderm surrounding the gut (Figure 3 (1-5 and Additional file 11: Figure S3)) in both the collar and trunk, consistent with a role in visceral mesoderm patterning. *foxC* is expressed in all the mesodermal compartments during initial specification and before out-pocketing of coeloms. However, our analysis did not detect later expression in the differentiated mesoderm making it difficult to speculate whether this gene plays a conserved role specifically in somatic mesoderm development.

In conclusion, our data provide support for an ancestral bilaterian chromosomal linkage of *foxQ1*-*foxF*-*foxC*-*foxL1*, and an evolutionarily conserved role of FoxF in visceral mesoderm patterning and FoxQ1 in foregut patterning. However, whether the early expression of *foxC* in mesoderm patterning is related to somatic mesoderm formation will require further investigation.

### Gill slit patterning (*foxC, foxI, foxJ1, foxL1)*

Hypotheses of homology of deuterostome pharyngeal gill slits have a long history in comparative studies [74]. Morphological and molecular studies in hemichordates support the homology of pharyngeal gills between ambulacrarians and chordates [75-79,212,213]. In this study we describe the expression of several Fox genes that can further contribute to this discussion. In *S. kowalevskii foxC*, *foxI, foxJ1*, and *foxL1* are expressed in the endoderm of the first gill pouch (Figure 2 (24/25), 3 (15), 3 (29/30)). Recent molecular data revealed that FoxC, and FoxL1 play conserved roles in gill slit formation in chordates, with conserved expression in the forming gill slit mesoderm (reviewed in Wotton et al. [34]). However, endodermal expression of *foxC* and *foxL1* during gill formation has only been described in the dogfish *Scyliorhinus canicula*[34]. Endodermal expression of FoxI during pharyngeal pouch development has been described in mice (*foxi3*[165]) and zebrafish (*foxi1*[214,215]). In *S. kowalevskii* endodermal expression of *foxI* is detected during the development of the first gill pouch (Figure 3 (15)), supporting a conserved role for this gene in deuterostome gill formation. Our data suggest that endodermal expression of FoxC, FoxL1, and FoxI was involved in patterning the pharyngeal gill endoderm in stem deuterostomes, extending the analysis of Gillis et al. [79] and further strengthening hypotheses of deuterostome gill slit morphological homology by reconstructing ancestral gene regulatory networks involved in early deuterostome pharyngeal endodermal patterning.

### Ventral endoderm, mesoderm, and ectoderm patterning *(foxB, foxD*)

Basic anatomical comparisons on the relative organization of organ systems across the dorsoventral axis of arthropods and chordates have resulted in hypotheses suggesting the equivalence of the dorsal side of chordates and the ventral side of arthropods. This so-called dorsoventral axis inversion hypothesis has gained molecular support from comparative developmental genetics [216,217] (reviewed in [218,219]). In *S. kowalevskii bmp2/4* and *chordin*, which are involved in mediating DV patterning in bilaterian groups, are expressed in the same relative position as protostomes during DV patterning; *bmp2/4* dorsally, and *chordin* ventrally, which is inverted relative to their expression in chordates. These data suggest a molecular inversion of the DV axis after the split of ambulacrarians and chordates [83,219]. In our survey, we revealed two Fox genes, *foxB* and *foxD*, with differential expression along the DV axis, further supporting this observation of DV inversion.

In chordates, FoxB and FoxD are consistently expressed dorsally. In *Xenopus laevis foxD1*, *foxD2*, and *foxD3* are all expressed in the dorsal mesoderm [122] and are necessary for dorsolateral mesoderm identity [130,143]. In protostomes FoxD expression is detected in the ventral nervous system of *D. melanogaster*[35], and the ventral mediolateral muscles of *C. elegans*[150,151] where it is known for its function in dorso-ventral cell migration and axon projection [152,153]. FoxB is expressed in the dorsal ectoderm and mesoderm in chordates (frogs [122,125], ascidians [59], and cephalochordates [128]). In protostomes, FoxB expression in *C. elegans* (*lin-31*) is localized to ventral ectodermal cells [129], and in *D. melanogaster*, the two FoxB orthologs (*FD4* and *FD5*) are expressed in the ventral nervous system [35].

The localized expression of FoxD and FoxB along the bilaterian DV axis raises the possibility of a link to the BMP/TGF-beta pathway. Limited comparative functional studies confirm a link between FoxD and the BMP/TGF-beta pathway [55,154,155,220,221]. In chordates (*Xenopus*), *foxD1* (*XBF-2*) is downstream of BMP-antagonists like Cerberus, Noggin, and Gremlin, and plays a role in maintaining dorsolateral mesoderm during gastrulation by downregulating BMP-4 [130,143]. In protostomes (*C. elegans*) FoxD (UNC-130) acts as a transcriptional repressor and inhibits the expression of UNC-129, a *C. elegans* TGF-beta ortholog [40,150,151]. However, expression data from sea urchins and ascidians are inconsistent with a link to BMP signaling [20,57]. In *S. kowalevskii*, *foxD* and *foxB* are expressed ventrally opposite the side of BMP expression [83] (Figure 2 (17-20), 2 (30)) (Figure 5) supporting a link to BMP signaling and DV patterning, but this will need to be functionally validated.

### Through gut evolution (*foxAB*, *foxD*, *foxI)*

In our study we found three Fox genes that are expressed either in the mouth or hindgut of the embryo. *foxAB*, is expressed in a circumferential ectodermal ring in the anterior collar groove in *S. kowalevskii* at the position where the mouth forms (Figure 2 (12-15)). *S. kowalevskii foxD* and *foxI* are expressed in the hindgut (Figure 2 (30), 3 (15)). FoxD is also expressed in the hindgut of several other deuterostome species, including the sea urchin *S. purpuratus*, the cephalochordate *B. floridae*, the frog *X. laevis*, and the fish *O. latipes*, and *D. rerio*[20,52,135,144,192] (illustrated in Figure 6 F-I). In the protostome groups examined, *D. melanogaster* and *C. elegans*, there is no support for a conserved role of FoxD in gut pattering [35,150]. Hindgut FoxI expression similar to *S. kowalevskii* has so far only been described in sea urchins, where *foxI* is expressed in the larval hindgut with strong expression on the aboral side [20].

A FoxAB ortholog was also identified in echinoderms [20], cnidarians [19], cephalochordates [21], and bryozoans [103]. Expression however, is only known from bryozoans where it is expressed only transiently in larval structures in the ciliated cleft and abapical ectodermal territory [103]. Further expression analyses will be required before evolutionary hypotheses of its role in mouth formation can be tested.

From these data we conclude that FoxD likely plays a conserved role in deuterostome hindgut patterning, FoxI was likely co-opted into hindgut patterning during ambulacrarian evolution, and a broader bilaterian role of FoxAB in mouth patterning will require additional data from other phyla.

For a literature summary, see Table 1 and for additional discussion on expression patterns see Additional file 12.

## Conclusions

Analyzing the expression patterns of Fox genes in the hemichordate *Saccoglossus kowalevskii* builds on comparative data from echinoderms and chordates and helps to reconstruct the evolutionary history of the developmental roles of this important family of transcription factors during deuterostome evolution. Further comparing these data to available studies from all metazoans helps us to construct more robust hypotheses about the role of Fox genes as components of evolutionarily conserved gene regulatory networks by distinguishing them from lineage specific co-option.

Our sequence analysis demonstrates that all 23 Fox genes of *S. kowalevskii* fall into their respective families. It further refines our understanding of the evolution and diversification of the FoxQ2 gene family revealing a split of this family deep in metazoan evolution. We provide evidence for a clustered arrangement of *foxQ1-foxF-FoxC-foxL1* in the *S. kowalevskii* genome, which has been proposed to be an ancestral feature of bilaterians.

From our expression analyses we propose several evolutionarily conserved expression domains. In multiple cases these gene expressions support hypotheses of anatomical homology between phyla; conserved expression of *foxC*, *foxI*, *foxJ1*, and *foxL1* during gill slit formation provides additional molecular support for the presence of pharyngeal gills in the common deuterostome ancestor; mesodermal *foxF* expression supports an evolutionarily conserved role for FoxF in visceral mesoderm patterning; expression of *foxQ1* supports an evolutionarily conserved role of FoxQ1 in pharyngeal endoderm patterning; *foxJ1* expression supports a conserved role of FoxJ1 in forming motile cilia throughout metazoans; and a conserved role of FoxI and FoxD in hindgut patterning in ambulacrarians and deuterostomes is supported by their expression pattern in *S. kowalevskii*, respectively. In other cases, expression in *S. kowalevskii* supports conserved interactions with signaling pathways such as FoxQ2 and Wnt/β-catenin signaling, and FoxB and FoxD with the BMP pathway.

Further studies of other phyla, particularly the lophotrochozoans, acoels, and cnidarians, are now required to broaden the phylogenetic scope of these comparisons. Functional studies are further required to confirm the proposed interactions of Fox genes with signaling pathways to further elucidate the evolution of this transcription factor family and its roles in embryonic patterning.

## Competing interests

The authors declare that they have no competing interests.

## Authors’ contributions

JHF conceived of the study, identified and cloned Fox genes which were not already present from previous work, carried out *in situ* hybridization experiments and phylogenetic analysis. JG, RMF, and CJL provided sequences, RMF helped with the phylogenetic analysis and provided bioinformatic tools. JHF, JG and CJL wrote the manuscript and all authors read, corrected and approved the final manuscript.

## Supplementary Material

Additional file 1: Table S1 *S. kowalevskii* Fox gene sequence references/ accession numbers. Click here for file

Additional file 2: Table S2 *S. kowalevskii* Fox gene prediction sequences. Click here for file

Additional file 3: Table S3 Sequence IDs for all sequences used for phylogenetic analysis. Click here for file

Additional file 4: Table S4 Alignment for Fox phylogeny (Figure 1a). Click here for file

Additional file 5: Table S5 EH I-like motif in the FoxQ2 family. Click here for file

Additional file 6: Table S6 Alignment for Figure 1b (FoxQ2 family). Click here for file

Additional file 7: Figure S1 Phylogenetic analysis of the FoxAB family. *S. kowalevskii* FoxAB groups together with previously found members of this new Fox gene family supporting the idea that this new family is a separate ancestral Fox family. Bayesian analysis was performed using the mixed amino acid substitution model applying four independent simultaneous Metropolis-coupled Markov Chains Monte Carlo in two independent simultaneous runs. The likelihood model was set to gamma rates = 4. A tree was sampled every 250 generations for two million generations. The first 25% of the sampled trees were excluded via ‘burnin’ prior to consensus tree calculation. *Xenopus laevis* FoxE4 was used as outgroup. The trees converged to a standard deviation of 0.0071. Maximum likelihood analysis was performed using the Le-Gascuel (LG) amino acid substitution model [101] with estimated proportion of invariable sites and gamma shape parameters. The number of substitution rate categories was set to 4. Starting tree was computed with BIONJ and 1,000 bootstraps were performed. The input alignment comprises 39 sequences with 78 characters (see Additional file 8: Table S7). For sequence accession numbers see Additional file 1: Table S1 and Additional file 3: Table S3. Baysian posterior probabilities are displayed on top of each branch and maximum likelihood values underneath each branch. Stars indicate differing tree topologies which lead to no support value at that position. Branches with posterior probabilities below 50% are condensed. Abbreviations: Hs: *Homo sapiens*; Bf: *Branchiostoma floridae*; Nv: *Nematostella vectensis*, Ci: *Ciona intestinalis*; Sk: *Saccoglossus kowalevskii*; Xl: *Xenopus laevis*; Sp: *Strongylocentrotus purpuratus*; Ce: *Caenorhabditis elegans*; Dm: *Drosophila melanogaster*; Hv: *Hydra vulgaris*; Hm: *Hydra magnipapillata*; Ch: *Clytia hemisphaerica*. Click here for file

Additional file 8: Table S7 Alignment for Additional file 1: Figure S1 (FoxAB family). Click here for file

Additional file 9: Figure S2 Fox gene cluster analysis. By using the current *S. kowalevskii* genome assembly at Baylor College of Medicine (BCM), the HudsonAlpha assembly, HudsonAlpha Institute for Biotechnology, AL (unpublished data), as well as by performing manual genome walks and bidirectional blasts we were able to identify two Fox clusters, a *foxQ2-1 - foxQ2-3* cluster and a *foxQ1-foxF-foxC-foxL1* cluster. *foxC* and *foxL1* are joined on one scaffold and *foxQ2-1* and *foxQ2-3* are closely linked on a separate scaffold. In addition, *foxF* clusters with the *foxC* -*foxL1* scaffold depending on the algorithm used (it is linked in the BCM assembly but not in the HudsonAlpha assembly). Further, we provide evidence of a link of *foxQ1* to the *foxF*, *foxC*, and *foxL1* containing scaffold by manual genome walking using unassembled trace sequences and by bidirectional blast of the scaffold ends (see Additional file 10: Table S8). However, even though no better match was found in the genome, the scaffold ends mostly contain repeats and a final assignment of *foxQ1* and *foxF* requires further characterization. The *S. kowalevskii foxQ2-1* and *foxQ2-3* cluster indicates a species-specific tandem duplication event. Red arrows indicate orientation of the genes, black arrows indicate the continuation of a scaffold, and distances are given in kilobase pairs underneath each cluster. Black line connecting *foxQ1* and the *foxF-foxC-foxL* cluster indicates area of manual genome walking. Click here for file

Additional file 10: Table S8 Bridging contigs for *foxQ1* and *foxL1*-*foxF* contig. Click here for file

Additional file 11: Figure S3 Additional stages and views of Fox gene expression patterns. Click here for file

Additional file 12 Discussing various Fox gene expression patterns and their potential evolutionary relevance. Click here for file
