# Supplementary material for: The Fox/Forkhead transcription factor family of the hemichordate Saccoglossus kowalevskii
Source: EvoDevo. 2014 May 7;5:17. doi: 10.1186/2041-9139-5-17 (PMC4077281; doi:10.1186/2041-9139-5-17)
Supplement: Additional file 1: Table S1 — S. kowalevskii Fox gene sequence references/ accession numbers. [file 2041-9139-5-17-S1.pdf]

Additional Table 1: *S. kowalevskii* Fox gene sequence references/ accession numbers

Gene name	Synonym	Gene bank ID	Prediction ID	Scaffold
foxA	forkhead box A	NP_001158426<br>ACG76356		17409
foxAB	fork-head boxA/B	NP_001164676<br>ADB22667		49913
foxB	forkhead box B1<br>forkhead box B	NP_001158435<br>ACH68432		44408
foxC	FoxC-like	NP_001158465<br>ACH68433		50907
foxD	fork-head box D	NP_001164677<br>ADB22668		5417
foxE	forkhead box E1<br>forkhead box E<br>tyroind transcription factor 2 (ttf2)	NP_001158436<br>ACH68434		53919
foxF	forkhead box F1<br>forkhead box F	NP_001158437<br>ACH68435		50907
foxG	brain factor 1 (bf1)<br>forkhead box G1<br>forkhead box G	AAP79301		211907
foxI	fork-head box I	XP_002734694	g13991.t1	19910
foxJ1	forkhead box J1	NP_001158438<br>ACH73226		3006
foxJ2	fork-head box2/3	ADB22670<br>XP_002731348		3506
foxK	fork-head box k	ADB22671<br>XP_002742034		51914
foxL1	forkheadbox L1	ACY92523<br>NP_001161544		50907
foxL2	fork-head box L2	XP_002738065	g24480.t1	33423
foxM	fork-head box M	XP_002731977	g4960.t1	6417
foxN1/4	forkhead box N	NP_001158439<br>ACH73227		25407
foxN2/3	fork-head box N2/3	ADB22674<br>XP_002739254		38909
foxO	forkhead box O	NP_001158440<br>ACH68436		53412
foxP	forkhead box P<br>forkhead box P2	NP_001158441<br>ACH73228		39907
foxQ1	fork-head box Q1	XP_002733006	g8330.t1	11909
foxQ2-1	fork-head box Q2/ QM, FoxQ2c	ADB22676<br>GU224267		11412
foxQ2-2	FoxQ2-like (Pit)<br>forkhead box Q2-like, FoxQ2b	ACY92525<br>NP_001161546		36407
foxQ2-3	FoxQ/D-like (Dold)<br>forkhead box Q/D-like, FoxQ2a, FoxQML	ACY92524<br>NP_001161545<br>GU075995		11412
