# Supplementary material for: The Fox/Forkhead transcription factor family of the hemichordate Saccoglossus kowalevskii
Source: EvoDevo. 2014 May 7;5:17. doi: 10.1186/2041-9139-5-17 (PMC4077281; doi:10.1186/2041-9139-5-17)
Supplement: Additional file 2: Table S2 — S. kowalevskii Fox gene prediction sequences. [file 2041-9139-5-17-S2.pdf]

Additional Table 2: ***S. kowalevskii*** **Fox gene prediction sequences**

Gene name	Prediction sequence
foxI<br><br>Prediction ID:<br>g13991.t1	CGTGTGCCGTGATTGGCCAACGGAGATGACGTCGTTGAAAGTTAACTTATATCAACGAAGTTAGCCGTGAATGGTAGTACA<br>TATGTTCACTTCTATGAATGATCCGTGGAAAAGACCGATTGCCAACATGTATCACCAACATAGCGTACAGAGATCGCCTCCA<br>AGCTATCTAAGTAATCCGGCGTTGGATTATACAACTAATCCGGCTTATCCGGCATCAATGTGGTTTAATGCGTCCGCTATGAG<br>TGCCGCTAATCCTTTTAATACTGGGTACGTTCCACCGAGTTATTCAACGCCGCCGCCACCACCAATGCTAACACCGAACCAT<br>CAAAGACAATACTTCCCGTGTTCGGCGAGTCTCAGCGCCACTGATTTCGGCTGGCTGTCGATACCGACGCAACAAGAACTC<br>TTTAAGCTTGTACGTCCACCGTATTCGTACTCCGCGCTGATTGCGATGGCTATTCAGTCCGCCGGGGAGAAGAAAATAACAC<br>TGAGCGGTATCTACAAGTATGTCTCAGACAACTTCCCATTCTACAAGAAGAGTAAAGCTGGCTGGCAGAATTCCATCCGCC<br>ACAATCTCTCACTAAACGACTGTTTCAAGAAGGTGCCTAGGAGTGAGGACGACCCAGGGAAGGGTAATTACTGGATGCTG<br>GACCCTAACTGCGAAAAAATGTTCGATAACGGCAACTTCCGAAGGAAACGTAAACGACGCGGTGATAGCAGTACCACCGC<br>AGCGAACAGCGGTGTCCAAGGCAGCAGTCTGAATTCCTCATCGTCCACTGCCAGCACGACCAGAGAAACAGCTAAGTCTC<br>AAACAAGTCACGCAGATACAGTCAAATCCACGAACAATGATACGACAATACTGACGGCGGCGACAACACCATCGCAAACA<br>CCAATCACGGACGCTCTGCTGAGCGGTGCTTACCATCAATCGATGGCATCACATGTGACTCCAAGCACAAACCAGTGTAAC<br>TTCAGCGTGAGTAGTCTCTTACAAAATCACTGTAGTTCTGGTCATATTTAGTAAAGCATTGCGTATTGTGCGGACATATCTAT<br>ACGCCTACTAAAGAGACAACCCATGACAAGAAAAAACATTTATTTCCATTACCTTCGTCGAGTATTTTCCGATTTACGAAAG<br>TATGGAAAGATGGTATATATTATAAAAGTTGTGTATATATTCTTTAAAACTTCAGAAATACATGATAGATTATATTAATTTGTTC<br>GACAGACACACATCATCGCGTAGTTGTTAGCAAATCGTCTGACGAATATGCCGTTAGATTTAACCCATTCATGTTGATGTGT<br>CGTGTGTATGGCCAATTACGAATATGCCAGCCACCATATATTTTAAAGCTACAAATCGACAAATCGGATTTTGTGAAACTTTC<br>TATCTTCATTTAATCGAGTAAGATCATGAGATTATTTTATTATTGTTTGAGAATTTGAAATATGTCTTGACATTATGTGATTACT<br>TTAGAACCTCGATCTTAATACAAGTAATATTCCGAGTCCAAAAAACGAATCTTTTAGTTGCATCTTCTTTAGGCGGATAACGC<br>CGAGTGAATGGCAGTTATGGCCTGACATTATTGTAATAAGACTTCTTTGCGTCAGTTTCATAGTTGTTAATTTTGAATGACGA<br>TCGATTGGGGTGAATACTCGGAATTAATATTATCAATTACAATAATTCTATTCAATCGATTGAGGTATTAGAAATTATGAATAAT<br>AGCAAGACGACCATTGGCTTGATCACCGGACATGGGAAATTGCCGCGACATTTAACGTTAGTAATATACATATACTGTTTTT<br>GTTTATTGTAAATATATTATATTTTGTGAAAATGTTATTAGAATTGACAGTATATATCAGTAAAACAATTCTCCATGATGAATAA<br>AATAGTTGGCTGTTT
foxM<br><br>Prediction ID:<br>g4960.t1	GGCTGGCGTAAACGAGATCTTAATTTAAAAACTCGACCTCCGCGTATTTTCAAAGTGTACATCCATGCCTGGCCGCGTCTTG<br>TTGTTCTATTTCGAATTTGACGCTTATCTTGGCTAAAATAAGCCCATGCTGGATAAGGCAAGACCCATGACGATTGACCTAA<br>AAATACCATTAATGGTGTGATGAAACTATTGAGAAAAACCTAAAAAGACAAGATTCATACTGAAATAATGGATAAATATAAT<br>CCAAGGAAATCTCGGGATCAGACAGACGGAGGACGAATTTTCCTAAATCATGACCTCACTTTTAAAACATGTGGAAGTGA<br>AGACAATGTGAAATCACTAATTGACAGGATGAGTAATCAGAAACATTCAAGAAAGGTTATCATTCTTCGTCATCCAAAACC<br>CAAGAGCACCTTGTCTGAAGAACCCCCAAACAAACAAGTCACCAGTAATGTCACAGGCATGTCCACAGTGTTGTCTAGTA<br>CATTCAAGTCCACAGAAAGCAGGAATACTGCAACTACTCTACAAAGTGTTCAACGGAATTCATTGCATGATACAATGTTGA<br>ATAATCTGCAAAGCCGTGACAAACATAATATGAACACAACTTGTGGCATGACTAGTATTCAGAATCGTACATCCTTATTAGGT<br>AATAACTCGTATGTTATGTACCCAAATATGGTGGAAAAGAATTGTACACGAAAGACGTCAATGACGCAAAGCTACAACAGT<br>GCCACCCAACAAGGTGTTAGGATGGCATCTGCAACTATGGTACCTATGCGCAAACAGTCAAAAGAGGCAGTTACAGAAGT<br>AATACAACCAGGAGAGCAGACCAGATTGAAGCCACAGGACGCCATGAATGACAGTTTAACCAATATTACATGGTTGGGAG<br>GTGCAAGTGTTAATCCTAACTTAGAGATAAAAGTTGAAAATGAACGATCAAAGGTAATGTCAATTCGGAAGGAGAGTCCGC<br>ATAAACGTCCCTCGTACTCGTATATGTCCATGATACAGTTTGCTATAAATAGTAAACCGGACAAAAAGATGACGCTACAAGA<br>GATTTACCATTGGGTGGAGTCTACTTTCCCTTACTTTCAGTCAGCAAAGCCAGGATGGAAGAATTCAATACGACACAACCT<br>GTCTCTACATGATGTCTTTGTACGTGAAAAGCCAGAGGTAAACGGAAAGTCGTCATTCTGGAAGTTAAAAGAAGAAGAAA<br>AACAAATAAAAGTACAACCCTTTACTGGGTACTCAGTTCCTGGTGCCCGTTCCAAAAATACACCAATCTTGCCAAGATTAA<br>CACAGTCTTATGCTATTATTCCTATTCCGTTAATGGTACAAGCTATGCCATGTATTTCGTCTCCTGTATTGACACCCTCCTTACC<br>CATCACATCGACCCCAGCACCAAATTTCTTCACACCAAGCGGTCCCGAAAGTACAATGACGATGCGCCCAATGTGTCTTTC<br>TAAGAATCTCAAACGATATGCACAGGAGAGTAATGGTGAACCAGGCAATAAAAAGGTCAAAATAGCTCCAAAGCTTTTGA<br>CTACTGACTCTTGTGAAAGCAATGCTAGAGTAAGCACAAGATACACATTCAATGGCTCATCTCCACATATATCCATAAATCA<br>GGATTCTGGAATGGATTCTATGATCTCAGAAACTGAGCATTGTGTCACCCATCAGCCAACTATTGGACGTTTACCCATGCCT<br>GAAATACCCAGAAAGAAACATGGTCGCAAATCTCGGAGACCGCACAAGATTGTCAAAGATGAAGATCAGCAAGAAAATGT<br>GCATTTTGACGTTAGTTTTTCAGAAATATTTCCACCCTTTGATGGTTTGGAGTCATCTCCTCTACGGGGTTTAATGAAAACTC<br>CACCAAAAATTGGTATCACTTTTACTTCAACTCCAAACAAAAATATGTCCAACATAGATGGACTGACTTCTTTTGGTTTTAC<br>ACCATTGAAGACACTGGACCATATTGATAGTGGAATTGAGTCTTCTCATAGGTCAAGCAACAGAAAGTTTGGTAATAACTT<br>GACTCCACTGGTGAACAATTTCTCAAGTCCAGGAGTAAACAGATTCTCAGACATGATTGGTTTCAGCAATGACCTTGTACT<br>GACTCCTGATGCTACCAAAGCTGTTGACTGTATTGACGGATCAGCATTCAACTTTGATTTTGCATCACCCAAACTGTTGACT<br>CCAAACAAATTTAAACTCAATAAATCAACAGATGGGGACGACTCACTGAGAATTGGCTCGCTCAGTGACCTTGGTCTGACC<br>GGGTTAACACCAATGAAAGAAAACAAAATGGAATCAAACCAGAGTTTTACTAAGTTATGGAATGATGGTAGAGGAATTCTA<br>AAATCTATTTGTTTGGGTTCGGAAAAAAAGATTTTAACACAATAATAGTATCATGAGGTTGTCATAGCAAGTGGATAATATAC<br>CACTACTACGAATGGTTGAATTTTGTATGAACCAAAGATATATTTGACATGTACTGTATGTTACATGGGACTATATGGAACCC<br>AGTACACTGCACTAATTGTGATGTATTATCATTCAGACACATTAAGAAATGATTACCATTATTATAGTCAATATAAAAGTTTTG<br>CTTGAGAACAATGTGCAAGACACATTACATCTTGAATGAATACATAATAACAATTGAACGAAGAATCTAAAGAAAACTACA<br>TACACCACATATGAATGGTACAAATAATCAGGATGAGTCACTTCTGATATATTATTTAAAAAACATGTAAAACCACCTGTTTA<br>AAATGGTTTACAAGAAACACGTGTTATTAGGTGAAAAGCATAATCAACAGTGCGTGTTTGAAGTAGTTATGTGTCTCTGTTA<br>TTGGATGTAATTGAGCCAATATGTCATTACAGGGAGAAAAAAAAATAACTGGAACAGCAAAGTAACTAATGTTGAGTTACT<br>GTGGTTTGGATGTCTGATAAAGAGCGGTGGTCACTGTGCAGTGCCTGTTAAAACAAAAAATGCATTTTACACACGTTGTAT<br>ATATCACTGAGTCCTATGTCGATGCAGCAAGATGCACTTCTATCAGAAACCTTGTAATGTTGATTGGCCATAAAGAGTTACA<br>GTCATTGAACAGTGTGCATTCACATTCCATGTAACAACCAGTGTATTAAACACATGTTGCATGGGATTCAAACACAGCACA<br>ACGACCTCCGACCTTTAATTAAGATATTACTTCTCAAACAAAAAACTAATCAACTAGCGAGTAACAAGTACAGTAAAGTGT<br>TTACTTGGATAAAATACAAAAATAGTTTTTTTGTTAATTAAAAACACTTCAATAATAAAAATTTGCTGTTTTG

Gene name	Prediction sequence
foxQ1<br><br>Prediction ID:<br>g8330.t1	CTAAACTTGTTTTCGTAAATGACTAGTAAGTAACTCTGTGTTCGTAACTGACTAGTATCTAACCTTGTTTTCATAACCGACTA<br>GTATCTAACCGTGTGTTCGTAACTGACTAGTATCTAACCGTGTGTTCGTAACTGATTAGTATCTAACCCTGTGTTCGTAACGG<br>ACTAGTAAGTAACTCTGTATTCGTAACTGATTAGTATCTACACTTGTTATCATAACCGACTAGTATCTAACCCTGTGTTCGTAA<br>CTGACTAGTAAGTAACTCTGTGTTTGTAACTGACTAGTATCTAAACTTGTTTTCATAACCGACTAGTATCTAACCGTGTGTTC<br>GTAACTGACTAGTATCTAACCCTGTGTTCGAAACTGACTAGTATCTAACCCTGTGTTCGTAACTGACTAGTATCTAACCCTGT<br>GTTCGTAACTGACTAGTATCTAACCGTGTGTTCGTAAGCAACTAGTATCTAAACTTGTTTTCATAACCGACTAGTATCTAACC<br>GTGTGTTCGTAACTGACTAGTATCTAACCCTGTGTTCGTAACTGACTAGTATCTAACCCTGTGTTCGTAACTGACTAGTATCT<br>AACCGTGTGTTCGTAACCAACTAGTATCTAAACTTGTTTTCATAACCGACGAGTATCTAACCGTGTGTTCGTAACTGACTAG<br>TATCTGATCATGTGTTCGTAACTGACTAGTATCTAAACTTGTTTTCGTAAATGACTAGTATGTAACCCTGTTTTCGTAACTGAC<br>TTGTATCTAACCTTGTTTTCGTAGCTGACTAGTATCTAACCTTGTGTTCGTAACTGACTAGTATCTAAACTTGTTTTCGTAAAT<br>GACTAGTAAGTAACTCTGTGTTCGTAACTGACTAGTATTTGACCTTGTGTTCGTAACTGACTAGTATCTAACGTGTGTTCGTA<br>ACTGACTAGTATCTAAACTTGTTTTCGACACTGACTAGTATCTAACCGTGTGTTCGTACATGACTAGAAGACACCACTGAGT<br>GAATGCCATCATCGCACTGAAGATTTACAAACTTTATGTGCGGAGTACATACTGATTAGTGAGTGTCATCCGGCGGGCACTT<br>TGGTTTTCGACATCCTATGGTAACGAAATCATTTTCGATAAGGATCGTTTTTGACAATAATTATACCTGGTATTTTTAAAAACG<br>AAGTTGGAAAGAGACATCGGACCGAAGATGCCCGAAACAGCAGATCCATCAACTATTATAGTTCCTTCAAGAAGTGTAGTT<br>GCGTCAAGCGAAAATGAGAACATCATTTTGAAAATGGATTTGTCAGACAACGAACACAGTATTAAAGATGAGCTGTCATAC<br>CGGTCTGATGATCAAAGTCACGAGGACAATTTAATGGAAGACAGAAACATAGAAAATACCATCTCCACAAGCACAACAAC<br>AACGACAACTACTTCTGGGAATTCGTCAGGAAAGGCGACATACCACCGACACCCCAAACCACCGTTATCGTACATTGCACT<br>AATTGCTGCGGCAATACAAGAATCGCCTACCAGGAAATTAACTTTGGCAGAGATCAACGACTACCTGATGAAGAAATATCC<br>CTTCTTTCGCGGTAGTTACACGGGTTGGCGGAACTCGGTCCGACATAATCTCTCTCTCAACGAATGTTTCACGAAAGTCCTT<br>CGTGACCCGTCTCGTCCTTGGGGAAAGGACAACTACTGGACAATCAATTGCAACAGTGAGTATACTTTCGCCGACGGCGTC<br>TTTCGGCGCAGAAGAAAAAGAATCAACAGGAAAAAGTCGAAAAAGAGCAAGGAGATTGGATTGACTGGCCATCATATCG<br>CGGTTGCAGGAGAGAAAAAATTCAACAGTCCCTTCGCTATTGATAATATCCTTGGAAAATCATCGGACGATGAGACTCATCT<br>ACTCCACACTGTGGCGGTGTCGGCTCCTTCGCACTGGCACAACGGATTTTCGACGGTAAATGGTATCGAGCACCAAAGACT<br>CATTTTGCAGCAGCAGCAGCAACAACATTTATATGAACGAAAATTGCTCGGTTACCCTGTCCCCAGTACGGGTCTGCATGCT<br>TTCTCTCCTGTTGCGGTAGCCGCTACAGCGCACCACGCGATCATTCCTGCTTATTACCAAAGTCTCCTGCAGTCCGGTTACA<br>GGTACCCGGCGATGGAGGCTCTCCAACAACACGTCCGCGCGCATAGTCACATCAGCGGCACGACACACCAACTACATTCC<br>CACCAGGCCACACAAGCCTCCGTGGCGGTTAAACCATCTACGGCTGCCATAGTGTACTCTACCATGATGCCAGAAGACTTA<br>CGCGTTAGCAGCAGACATCAGCGGGACGGTGCGAGCTCACAGACGCGGCGAGCAGAGCCGTCGCTCCGACAAAGTACAA<br>GTAGTTTCAGCGTGGAAAATTTGATATCTTAGGAACAGTGTTAATATGTATTAATACAGGTATCGTAGTGTATGTGTTCCCGA<br>GCTTCAACCAAACTCTGTTAAGTCGGTGAGCACGTCCTGAAATGCAACATAAATTGATAATCGCATTACACCGTTGTCAAG<br>ATGACATTGTTTAAACGTTGGTGGACTTTAGTCGAACATGGAACGGATAAAACGCACCACAATTGTATGCGAGGCAGTAGC<br>CGCTCGGGTGGTATAAAATATCTCTGTCAAATCGGCAGCGTTAGGTGTGTATAATACGATATTTAAGATCCAAATTAAAATCA<br>CTGGCTGCATGTTTTTTTCTTTTCTTCAAATGTATGTACTAAAAAGGCAATTCTGAGAAACCCATAAATATGGCGTTTCTAAG<br>TACTAGTATTACAGTGAATTCATATTTAGGGCTGTTTTTTTTTAATTTCAGGGTTAATGTAATAAATGTTATTGTGATTT
foxL2<br><br>Prediction ID:<br>g24480.t1	TTTGGAGTTTGTGCACGGTTAACTAGATAATACCCGTGATGCCCTGTTTTCTTTTTATATTTTGCTATTTTCTGTATTATGAACG<br>AGAAAGACATGGAGAACGTGATCAACGATTTACGAGTTAACATCAAGAAGGAGCCCGATGCCGAAAAGTTCCGATGCAAC<br>GGACTGGAAAGTGATGAACATCTAGCCAAGGTTGTGGAATTGAAACCGATCGGCAGTATCCAGACCATGAATGAGGCGGT<br>CAAAAAAGAAACAGATACTGACAAGAAATCGGAGGAGAACCACCCCAAACCACCGTATTCATATGTTGCTTTAATCGCAAT<br>GGCAATACGGGAAAGTCAAGAAAAACGTCTGACTTTGAGTCAAATCTACGACTTTATCGTGAACAAGTTCCCATTCTATGA<br>AAAGAACAAGAAAGGTTGGCAGAACTCAATCAGACATAATCTAAGTTTGAATGAATGTTTTATCAAGATCCCGCGCGAAG<br>GAGGTGGGGAGAGGAAAGGAAATTTCTGGACGCTAGACCCAGCGTGTGAAGATATGTTCGAGAAAGGAAACTATCGTCG<br>CCGTCGCCGTATGAAGGCAAGACCGTACAGGACATCGGCGCAATCAGCATGCTACGAACCCAAGTCGCTGCTGACAGCTG<br>ATCCGACCTATGGAGGGGGATACCCACTATCGCATAAATATCTACCAAGTGCTTATAACAATTGGCCTGCGGTTCATCATGGC<br>CAAACTACACCAAGTCAACTCGGTTATAGCAGTTGCCAAACTCCTTCGCGATTACCACAGACTGTAGAAATAAACTACCCA<br>AGCAGCGGCTCGATGCAGCTCCCCGGCTATAGCCAAGTGGATCTGAGTCCAATGTCAACAATGAATGGTTCCACCCCGGGT<br>AGCGTGAGTTACAGTGCTTACGCGTTCCCCTGTCATCGCAATGCCGCTGAATCCGCTATGTCCTCGTACGCTTACTGGACCG<br>CCGAGAAGCAACACGTCGGTAATCACCTCATTAATTGACAATCGGATTTTAATTGTTTAGAAGCATTCAATACTGCGACCTA<br>GCAAGTATAAACTCTTGTTACCATTTAATGAGCGTGTCGATTGATTATTTCATGCCGTTATGTTTGTGTCAGTTAATAATTTGT<br>TCTAGGTTTAACATTTGTGTTATTTCACTCTTATCGAAGTTGTTAGGGACCAATTTTATAAATTACGTGAAACAGTTTAAAGT<br>TTAAAACTAGCACCAGAACATTTTCTGGCTAAAATAAATCATATATTCATTTTCATAGTATTTTCATGTCAGAAAGACTTTATT<br>TGTTTGTGTCTGTTGTTAAACTTGGGAAATGTCTGGACCAAGCCCACAAGTTCAAAACGAATATGATTTATGTAAGTTTAAT<br>ATTTCAAAGATGAGAGATGGTTATTATACGTAACCAGATTTATAAATTCATGTTTATTATATACTGTTGATAGATTAAAACATAT<br>TTTTATTG
