# Supplementary material for: The Fox/Forkhead transcription factor family of the hemichordate Saccoglossus kowalevskii
Source: EvoDevo. 2014 May 7;5:17. doi: 10.1186/2041-9139-5-17 (PMC4077281; doi:10.1186/2041-9139-5-17)
Supplement: Additional file 3: Table S3 — Sequence IDs for all sequences used for phylogenetic analysis. [file 2041-9139-5-17-S3.pdf]

Additional Table 3: Sequence ID's for all sequences used for phylogenetic analysis

Abbreviation	Definition	NCBI accession number
**Homo sapiens**		
HsFoxA1	forkhead box A1	NP_004487
HsFoxA2	forkhead box A2	NP_068556
HsFoxA3	forkhead box A3	NP_004488.2
HsFoxB1	forkhead box B1	NP_036314
HsFoxB2	forkhead box B2	NP_001013757
HsFoxC1	forkhead box C1	NP_001444
HsFoxC2	forkhead box C2	NP_005242
HsFoxD1	forkhead box D1	NP_004463
HsFoxD2	forkhead box D2	NP_004465
HsFoxD3	forkhead box D3	NP_036315
HsFoxD4	forkhead box D4	NP_997188
HsFoxE1	forkhead box E1	NP_004464
HsFoxE3	forkhead box E3	NP_036318
HsFoxF1	forkhead box F1	NP_001442
HsFoxF2	forkhead box F2	NP_001443
HsFoxG1	forkhead box G1	NP_005240
HsFoxH1	forkhead box H1	NP_003914
HsFoxI1	forkhead box I1	NP_036320
HsFoxI2	forkhead box I2	NP_997309
HsFoxJ1	forkhead box J1	NP_001445
HsFoxJ2	forkhead box J2	NP_060886
HsFoxJ3	forkhead box J3	NP_055762
HsFoxK1	forkhead box K1	NP_001032242
HsFoxK2	forkhead box K2	NP_004505
HsFoxL1	forkhead box L1	NP_005241
HsFoxL2	forkhead box L2	NP_075555
HsFoxM1	forkhead box M1	NP_973731
HsFoxN1	forkhead box N1	NP_003584
HsFoxN2	forkhead box N2	NP_002149
HsFoxN3	forkhead box N3	NP_001078940
HsFoxN4	forkhead box N4	NP_998761
HsFoxO1	forkhead box O1	NP_002006
HsFoxO3	forkhead box O3	NP_963853
HsFoxO4	forkhead box O4	NP_005929
HsFoxP1	forkhead box P1	NP_116071
HsFoxP2	forkhead box P2	NP_001166237
HsFoxP3	forkhead box P3	NP_054728
HsFoxP4	forkhead box P4	NP_001012427
HsFoxQ1	forkhead box Q1	NP_150285
HsFoxR1	forkhead box R1	NP_859072

Abbreviation	Definition	NCBI accession number
HsFoxR2	forkhead box R2	NP_940853
HsFoxS1	forkhead box S1	NP_004109
**Strongylocentrotus purpuratus**		
SpFoxA	forkhead transcription factor A	DQ459376
SpFpxAB	forkhead transcription factor A /B-Like	DQ286736
SpFoxB	forkhead transcription factor B	NM_214632
SpFoxC	forkhead transcription factor C	DQ286740
SpFoxD	forkhead transcription factor D	DQ286738
SpFoxF	forkhead transcription factor F	DQ286741
SpFoxG	forkhead transcription factor G	DQ286739
SpFoxI	forkhead transcription factor I	DQ286747
SpFoxJ1	forkhead transcription factor J1	DQ286742
SpFoxJ2	forkhead transcription factor J2	DQ286737
SpFoxK	forkhead transcription factor K	DQ286748
SpFoxL1	forkhead transcription factor L1	DQ286750
SpFoxL2	forkhead transcription factor L2	DQ286745
SpFoxM	forkhead transcription factor M	DQ286752
SpFoxN14	similar to forkhead transcription factor N1/4 transcript variant 1	XP_001194048
SpFoxN23	forkhead transcription factor N2/3	DQ286744
SpFoxO	forkhead transcription factor O	DQ286746
SpFoxP	forkhead transcription factor P	DQ286749
SpFoxQ1	forkhead transcription factor Q1	DQ286751
SpFoxQ2	forkhead transcription factor Q2	DQ286735
SpFoxX	forkhead transcription factor X	DQ286743
SpFoxY	forkhead transcription factor Y	AF517552
**Branchiostoma floridae**		
BfFoxA_a	forkhead transcription factor FoxAa	ACE79136
BfFoxA_b	forkhead transcription factor FoxAb	ACE79149
BfFoxAB	forkhead transcription factor FoxA/B	ACE79151
BfFoxB	forkhead transcription factor FoxB	ACE79150
BfFoxA_a	forkhead transcription factor FoxAa	ACE79136
BfFoxA_b	forkhead transcription factor FoxAb	ACE79149
BfFoxAB	forkhead transcription factor FoxA/B	ACE79151
BfFoxB	forkhead transcription factor FoxB	ACE79150
BfFoxC	forkhead transcription factor FoxC	ACE79154
BfFoxD	forkhead transcription factor FoxD	AF512537_1
BfFoxEa	forkhead transcription factor FoxEa	ACE79155
BfFoxEb	forkhead transcription factor FoxEb	ACE79156
BfFoxF	forkhead transcription factor FoxF	CAH69695
BfFoxG	forkhead transcription factor FoxG	AAC18392
BfFoxH	forkhead transcription factor FoxH	ACE79158

Abbreviation	Definition	NCBI accession number	Abbreviation	Definition	NCBI accession number
BfFoxI	forkhead transcription factor FoxI	XP_002608285	ChFoxQ2a	forkhead box-containing transcription factor FoxQ2a	ABG21224
BfFoxJ1	forkhead transcription factor FoxJ1	ACE79142	ChFoxQ2b	forkhead box-containing transcription factor FoxQ2b	ABG21225
BfFoxJ23	forkhead transcription factor FoxJ2/3	XP_002591636	**Hydra vulgaris**		
BfFoxK	forkhead transcription factor FoxK	ACE79146	HvFoxA	budhead [Hydra vulgaris]	AAO92606
BfFoxL1	forkhead transcription factor FoxL1	XP_002594265	HvFkh2	Fkh2 [Hydra vulgaris]	Chevalier *et al.*, 2006
BfFoxL2	forkhead transcription factor FoxL2	XP_002599263	HvFkh3	Fkh3 [Hydra vulgaris]	Chevalier *et al.*, 2006
BfFoxM	forkhead transcription factor FoxM	ACE79148	**Hydra magnipapillata**		
BfFoxN14a	forkhead transcription factor FoxN1/4a	ACE79139	HmFoxA	similar to forkhead box-containing transcription factor FoxQ2b	Chevalier *et al.*, 2006
BfFoxN14b	forkhead transcription factor FoxN1/4b	ACE79137	HmFoxK	similar to forkhead box-containing transcription factor FoxK	Chevalier *et al.*, 2006
BfFox23	forkhead transcription factor FoxN2/3	ACE79140	HmFoxQ2a	similar to forkhead box-containing transcription factor FoxQ2a	XP_002165467
BfFoxO	forkhead transcription factor FoxO	ACE79159	HmFoxQ2b	similar to forkhead box-containing transcription factor FoxQ2b	XP_002159677
BfFoxP	forkhead transcription factor FoxP	XP_002613659	HmFoxQ2c	similar to forkhead box-containing transcription factor FoxQ2c	XP_002166935
BfFoxQ1	forkhead transcription factor FoxQ1	ACE79143	**Ciona intestinalis**		
BfFoxQ2a	forkhead transcription factor FoxQ2a	ACE79145	CiFoxA	forkhead homolog	NP_001027657
BfFoxQ2b	forkhead transcription factor FoxQ2b	XP_002613661	CiFoxB	FoxB protein	NP_001027695
BfFoxQ2c	forkhead transcription factor FoxQ2c	ACE79147	CiFoxC	transcription factor protein	NP_001071708
BfFox1	forkhead transcription factor Fox1	XP_002604922	CiFoxD	transcription factor protein	NP_001071709
**Nematostella vectensis**			CiFoxE	foxE protein	NP_001027947
NvFoxA	forkhead domain protein A	AAS13442	CiFoxF	transcription factor protein	NP_001071710
NvFoxB	forkhead domain protein B	ABA03229	CiFoxG	transcription factor protein	BAE06438
NvFoxC	forkhead domain protein C	ABA03230	CiFoxH	transcription factor protein	XP_002129743
NvFoxD1	forkhead domain protein D1	ABA03231	CiFoxI	transcription factor protein	NP_001071713
NvFoxD2	forkhead domain protein D2	XP_001637168	CiFoxJ2	transcription factor protein	NP_001071714
NvFoxE	forkhead domain protein E	ABA03232	CiFoxK	transcription factor protein	NP_001071715
NvFox1	forkhead domain protein 1	ABA03228	CiFoxL2	FoxL protein	NP_001027769
NvFox2	forkhead domain protein 2	XP_001637106	CiFoxM	transcription factor protein	NP_001071993
NvFox3	forkhead domain protein 3	XP_001631592	CiFoxN23	transcription factor protein	NP_001071716
NvFox4	forkhead domain protein 4	Magie *et al.*, 2005	CiFoxO	transcription factor protein	NP_001071717
NvFox5	forkhead domain protein 5	XP_001634717	CiFoxP	transcription factor protein	NP_001071939
NvFox6	forkhead domain protein 6	Magie *et al.*, 2005	CiFoxQ1	transcription factor protein	NP_001071718
NvFoxL2	forkhead domain protein L2	XP_001639875		**Xenopus laevis**	
NvFoxO1	forkhead domain protein O1	Magie *et al.*, 2005	XlFoxA4	forkhead box protein A4-A	NP_001080963
NvFoxO2	forkhead domain protein O2	Chevalier et al., 2006	XlFoxE4	forkhead box protein E4	NP_001079202
NvFoxNx	forkhead domain protein Nx	XP_001641147		**Danio rerio**	
**Clytia hemisphaerica**			DrFoxI3	forkhead box I3a	NP_944599
ChFoxB	forkhead box-containing transcription factor FoxB	ABG21223	DrFoxQ2	forkhead box Q2	NP_001098411
ChFoxO	forkhead box-containing transcription factor FoxO	ABG21226			

Abbreviation	Definition	NCBI accession number		Abbreviation	Definition	NCBI accession number
	**Drosophila melanogaster**				**Caenorhabditis elegans**	
DmFoxA	fork head, isoform A	NP_524542		CeFoxA	defective PHArynx development family member (pha-4)	NP_001041114
DmFoxB	forkhead domain 96Ca	NP_524495		CeFoxB	abnormal cell LINeage family member (lin-31)	NP_494704
DmFoxC	crocodile	NP_524202		CeFoxD	UNCoordinated family member (unc-130)	NP_496411
DmFoxD	forkhead domain 59A	NP_523814		CeDAF16	abnormal DAuer Formation family member (daf-16)	NP_001021593
DmFoxF	biniou	NP_523950		CeFkh2	ForKHead transcription factor family member (fkh-2)	NP_508644
DmSlp1	sloppy paired 1	NP_476730		Celet381	LEThal family member (let-381)	NP_491826
DmSlp2	sloppy paired 2	NP_476834		CeFkh7	ForKHead transcription factor family member (fkh-7)	NP_001023147
DmFoxK	Mnf, isoform G	NP_648440		CeFkh10	ForKHead transcription factor family member (fkh-10)	NP_492676
DmFoxN14	jumeau	NP_524302				
DmFoxN23	checkpoint suppressor homologue, isoform B	NP_996362			**Suberites domuncula**	
DmFoxO	forkhead box, sub-group O, isoform C	NP_996204		SdFoxF	forkhead foxF	CAE51210
DmFoxP	CG16899	NP_001097726		SdFoxD	forkhead foxD	CAE51209
DmFoxQ2	CG11152	NP_651951		SdFoxL2	forkhead foxL2	CAE51212
	**Saccharomyces cerevisiae**			SdFox1	forkhead fox1	CAE51213
SccFox1	Fkh1p	CAY80377		SdFoxP	forkhead foxP	CAE51211
SccFox2	Fkh2p	NP_014331			**Amphimedon queenslandica**	
	**Drosophila grimshawi**			ReFoxL1	FoxL1-like	ACA04752
DgFoxL1	GH15375	XP_001983802		ReFoxJ		
	**Mnemiopsis leidyi**				**Schizosaccharomyces pombe**	
MlFoxG	brain factor 1-like protein	AF477500_1		SchFox2	fork head transcription factor Fkh2	NP_596764
	**Debaryomyces hansenii**				**Aspergillus niger**	
DebFkh	DEHA2A08910p	XP_456717		AspFkh2	hypothetical protein An02g07900	XP_001399923
