# Supplementary material for: The Fox/Forkhead transcription factor family of the hemichordate Saccoglossus kowalevskii
Source: EvoDevo. 2014 May 7;5:17. doi: 10.1186/2041-9139-5-17 (PMC4077281; doi:10.1186/2041-9139-5-17)
Supplement: Additional file 4: Table S4 — Alignment for Fox phylogeny (Figure 1a). [file 2041-9139-5-17-S4.pdf]

Additional Table 4. Alignment for Fox phylogeny (Figure 1a)

```
SccFox1     KPPQSYASMITQAILSTPEGSISLADIYKFISDNYAFYRF-----SQMAWQNSVRHNLSLNKAFEKVPKR----AGQ-QGKGMNWKIS
HsFoxN1     KPIYSYSILIFMALKNSKTGSLPVSEIYNFMTEHFPYFKT-----APDGWKNSVRHNLSLNKCFEKVENK----SGSSSRKGCLWALN
HsFoxN4     KPIYSYSCLIAMALKNSKTGSLPVSEIYSFMKEHFPYFKT-----APDGWKNSVRHNLSLNKCFEKVENK----MSGSSRKGCLWALN
SkFoxN14    KPAYSYSCLIAMALKNSKTGCLPVSEIYHFMCENFPYFKT-----APDGWKNSVRHNLSLNKCFEKIEKP---PGGGSTRKGCLWALN
SpFoxN14    KPAYSYSCLITMSLKNSQNGCLPVSEIYQFMCENFPYFKT-----APDGWKNSVRHNLSLNKCFAKIEKPQVNNGNGSARKGCLWAMN
BfFoxN14a   KPAYSYSCLIAMALKNSKTGCLPVSEIYNFMCDNFPYFKT-----APDGWKNSVRHNLSLNKCFEKVEKS----TGGTSKKGCLWTLN
DmFoxN14    KPAYSYSCLIALALKNSRAGSLPVSEIYSFLCQHFPYFEN-----APSGWKNSVRHNLSLNKCFEKIERP----ATNGNRKGCRWAMN
BfFoxN14b   KPPYSYSCLIALAMMKSRTGNMPVTEIYSFIREHYPYFKT-----APDGWKNSVRHNLSLNKCFLKIEPS----QADPNRKGCLWALH
BfFoxM      RPPYSYMALIQFAINSSPARRMTLKEIYTWIEGRFPYFKT-----AKQGWKNSIRHNLSLHDIFIRETTK----C-ESGRALSYWTLC
CiFoxM      RPPYSYMALIQFAINSSSTGKMTLRQIYQWIEEKFPYFKT-----AKPGWKNSIRHNLSLHDIFVRQVT--------TGSKASYWTLR
HsFoxM1     RPPYSYMAMIQFAINSTERKRMTLKDIYTWIEDHFPYFKH----IAKPGWKNSIRHNLSLHDMFVRETS--------ANGKVSFWTIH
SpFoxM      RPPYSYSSLIQFAISSAPEGKLTLRDVYFWIETHFPYFRT-----AKLGWKNSIRHNLSLHKIFVREAP-------SGPGQPAFWTLR
SkFoxM      RPSYSYMSMIQFAINSKPDKKMTLQEIYHWVESTFPYFQS-----AKPGWKNSIRHNLSLHDVFVREKP-------EVNGKSSFWKLK
HsFoxO1     WGNLSYADLITKAIESSAEKRLTLSQIYEWMVKSVPYFKDKGDSNSSAGWKNSIRHNLSLHSKFIRVQN-------EGTGKSSWWMLN
BfFoxO      WGNLSYADLITKAIQSSPEGRLTLSQIYDWMVRCVPYFRDKGDSNSSAGWKNSIRHNLSLHSRFIRVQN-------EGTGKSSWWMLN
SkFoxO      WGNLSYADLITKAIESAPDKRLTLSQIYEWMVKSVPYFKDKGDSNSSAGWKNSIRHNLSLHSRFVRVQN-------EGTGKSSWWMIN
SpFoxO      WGNLSYADLITKAIQSAPDQRLTLSQIYDWMVKNVPFFKDKGDSNSSAGWKNSIRHNLSLHSRFVRVQN-------EGTGKSSWGXST
HsFoxO3     WGNLSYADLITRAIESSPDKRLTLSQIYEWMVRCVPYFKDKGDSNSSAGWKNSIRHNLSLHSRFMRVQN-------EGTGKSSWWIIN
DmFoxO      WGNLSYADLITHAIGSATDKRLTLSQIYEWMVQNVPYFKDKGDSNSSAGWKNSIRHNLSLHNRFMRVQN-------EGTGKSSWWMLN
CiFoxO      WGNMSYADLITQGIESSPDKRLTLAQIYDWMVKNVPYFKDKGDSNSSAGWKNSIRHNLSLHSKFKRIQN-------EGTGKSSWWIIN
NvFoxO_2    WGNYSYADLITQAIQSSPEKRLTLSQIYDWMVNSVPYFRDKGDSNSSAGWKNSIRHNLSLHSKFVRVQN-------EGNGKSSWWVLN
HsFoxO4     WGNQSYAELISQAIESAPEKRLTLAQIYEWMVRTVPYFKDKGDSNSSAGWKNSIRHNLSLHSKFIKVHN-------EATGKSSWWMLN
ChFoxO      WGNLSYADLITRAIQSSPEQRLTLSQIYEWMVRNIPYFKDKGDSTSSAGWKNSIRHNLSLHSRFMRVQN-------DNNGKSSYWVIN
NvFoxO_1    WGSESYSEMISKAIMSCPSQEATLHTIYEWIVNNVSYFADKADYPSTHGWKNSIRHNLSLHSKFVRVQN-------EGNGKSSWWVLN
CeDAF16     WGEESYSDIIAKALESAPDGRLKLNEIYQWFSDNIPYFGERSSPEEAAGWKNSIRHNLSLHSRFMRIQN-------EGAGKSSWWVIN
HsFoxF1     KPPYSYIALIVMAIQSSPTKRLTLSEIYQFLQSRFPFFRG-----SYQGWKNSVRHNLSLNECFIKLPKG----LGRP-GKGHYWTID
BfFoxF      KPPYSYIALIVMAIQSSATKRLTLSEIYQFLQQRFPFFRG-----PYQGWKNSVRHNLSLNECFIKLPKG----LGRP-GKGHYWTID
HsFoxF2     KPPYSYIALIVMAIQSSPSKRLTLSEIYQFLQARFPFFRG-----AYQGWKNSVRHNLSLNECFIKLPKG----LGRP-GKGHYWTID
SkFoxF      KPPYSYIALIVMAIQSSPTKRLTLSEIYQFLMNRFPFFRG-----PYQGWKNSVRHNLSLNECFIKLPKG----LGRP-GKGHYWTID
SpFoxF      KPPYSYIALIVMAIQSSPAKRLTLSEIYQFLMQRFPFFRG-----PYQGWKNSVRHNLSLNECFIKLPKG----LGRP-GKGHYWTID
CiFoxF      KPPYSYIALIVMAIQSSPAKKLTLSEIYNFLQTRFEFFRG-----AYQGWKNSVRHNLSLNECFIKLPKG----LGRP-GKGHYWTID
DmFoxF      KPALSYINMIGHAIKESPTGKLTLSEIYAYLQKSYEFFRG-----PYVGWKNSVRHNLSLNECFKKLPKG----MGKP-GKGNYWTID
CeLet381    --PFSYIALIAMAISKRPDKKATLAEIYSYLQENFEFFRG-----EYAGWRNSIRHNLSLNECFVKLPKD----TGES-GKGHKWTIS
HsFoxH1     KPPYTYLAMIALVIQAAPSRRLKLAQIIRQVQAVFPFFRE-----DYEGWKDSIRHNLSSNRCFRKVPKD----PAKPQAKGNFWAVD
BfFoxH      KPPYSYLALVVMAIQNAPEKKLPLKEIHEALKKMYPFFRG-----DYTGWKDSVRHNLSTYKCFYKVPKD----PSRPFAKGNYWAVY
BfFox1      KPPVSYTAMIAAVIQESPEKKLTLLQIVDELKKRYSFFNG-----DYKGWKNSVRHNLSLNKCFVKVPRD----ADRPFGKDNFWTVD
CiFoxH      KPPYSYVSLITLSILSSPEKKLRLSQILKRISEMFPFFNG-----SYQGWRDSVRHNLSQNECFVKVLKN----PYRPTAKGNYWTVN
SpFoxQ1     KPPFSYIALIAMAIRDSGHGKLTLAEINEYLMKKFPFFRG-----SYTGWRNSVRHNLSLNECFRKILRD----PSRPWGKDNYWTIN
CiFoxQ1     KPPFSYIALIAMAIRDSANGKLTLAEINEYLMKKFPFFRG-----SYTGWRNSVRHNLSLNECFQKILRD----PSRPWGKDNYWTIN
BfFoxQ1     KPPFSYIALIAMAIRDSPNNRLTLAEINDYLMKKFEFFRG-----PYTGWRNSVRHNLSLNECFTKVLRD----PSRPWGKDNYWTIN
SkFoxQ1     KPPLSYIALIAAAIQESPTRKLTLAEINDYLMKKYPFFRG-----SYTGWRNSVRHNLSLNECFTKVLRD----PSRPWGKDNYWTIN
HsFoxQ1     KPPYSYIALIAMAIRDSAGGRLTLAEINEYLMGKFPFFRG-----SYTGWRNSVRHNLSLNDCFVKVLRD----PSRPWGKDNYWMLN
HsFoxA1     KPPYSYISLITMAIQQAPSKMLTLSEIYQWIMDLFPYYRQ-----NQQRWQNSIRHSLSFNDCFVKVARS----PDK-PGKGSYWTLH
HsFoxA3     KPPYSYISLITMAIQQAPGKMLTLSEIYQWIMDLFPYYRE-----NQQRWQNSIRHSLSFNDCFVKVARS----PDK-PGKGSYWALH
XlFoxA4     KPPYSYISLITMAIQQAPNKMMTLNEIYQWIIDLFPYYRQ-----NQQRWQNSIRHSLSFNDCFVKVPRS----PEK-PGKGSYWTLH
HsFoxA2     KPPYSYISLITMAIQQSPNKMLTLSEIYQWIMDLFPFYRQ-----NQQRWQNSIRHSLSFNDCFLKVPRS----PDK-PGKGSFWTLH
NvFoxA      KPPYSYISLITMAIQQSPNKMLTLSEIYQFIMDLFPYYRQ-----NQQRWQNSIRHSLSFNDCFVKVPRS----PDR-PGKGSYWTLH
CiFoxA      KPPYSYISLITMALQSSPNKMMTLSEIYNWIMDLFPFYRQ-----NQQRWQNSIRHSLSFNDCFVKVPRS----ADK-PGKGSYWSLH
CeFoxA      KPPYSYISLITMAIQKSNSRQLTLSEIYNWIMDLFPYYQN-----NQQRWQNSIRHSLSFNDCFVKVARS----PDK-PGKGSFWTLH
SkFoxA      KPPYSYISLITMAIQSSPNKMVTLSDIYQFIMDLFPFYRQ-----NQQRWQNSIRHSLSFNDCFLKVPRT----PDR-PGKGSFWTLH
SpFoxA      KPPYSYISLITMAIQQSPQKMVTLSDIYQFIMDLFPFYRQ-----NQQRWQNSIRHSLSFNDCFVKVPRT----PDR-PGKGSFWTLH
BfFoxA_a    KPPYSYISLITMSIQSSPNKMVTLAEIYQFIMDLFPYYRQ-----NQQRWQNSIRHSLSFNDCFVKVPRT----PDR-PGKGSYWTLH
BfFoxA_b    KPPYSYIALITMAVQSSPNKMVTLSEIYQFIMDLFPFYRQ-----NQQRWQNSIRHSLSFNDCFVKVQRT----PDR-PGKGSYWTLH
DmFoxA      KPPYSYISLITMAIQNNPTRMLTLSEIYQFIMDLFPFYRQ-----NQQRWQNSIRHSLSFNDCFVKIPRT----PDK-PGKGSFWTLH
HvFoxA      KPPYSYISLITMSIQNTPGKAVTLSEIYQFIMDHFPYYRQ-----NQQRWQNSIRHSLSFNDCFIKVPRS----PDK-PGKGSFWTLH
HmFoxA      KPPYSYISLITMSIQNTPGKAVTLSEIYQFIMDHFPYYRQ-----NQQRWQNSIRHSLSFNDCFIKVPRS----PDK-PGKGSFWTLH
HsFoxB1     KPPYSYISLTAMAIQSSPEKMLPLSEIYKFIMDRFPYYRE-----NTQRWQNSLRHNLSFNDCFIKIPRR----PDQ-PGKGSFWALH
BfFoxB      KPPYSYISLTAMAIQSSGEKMLPLSDIYKFIMDRFPFYRQ-----NTQRWQNSLRHNLSFNDCFIKIPRR----PDQ-PGKGSFWALH
HsFoxB2     KPPYSYISLTAMAIQHSAEKMLPLSDIYKFIMERFPYYRE-----HTQRWQNSLRHNLSFNDCFIKIPRR----PDQ-PGKGSFWALH
SkFoxB      KPPYSYIALTAMAIQSSTEKMLPLSDIYKFIMDRFPFYRK-----NTQRWQNSLRHNLSFNDCFIKIPRR----PDR-PGKGSYWALH
SpFoxB      KPPYSYISLTAMAIQSSQEKMLPLSDIYKFIMDRFPYYRK-----NTQRWQNSLRHNLSFNDCFLKIPRR----PDR-PGKGSYWALH
DmFoxB      KPPYSYISLTAMAIWSSPEKMLPLSDIYKFITDRFPYYRK-----NTQRWQNSLRHNLSFNDCFIKVPRR----PDR-PGKGAYWALH
CeFoxB      KPPYSYIWLTYMAIQDSDDKMLPLTEIYKYIMDRFPFYRK-----NTQRWQNSLRHNLSFNDCFIKIPRR----ADR-PGKGSYWAVH
CiFoxB      KPPYSYIALTAMAIQSSPNKMMSLSEIYRYIMDRFPFYRN-----NTQRWQNSLRHNLSFNDCFVKVPRR----GDQ-PGKGSLWSLH
NvFoxB      KPPYSYISLTAMAIQSSPQKMLSLSEIYQFIMDHFPFYRD-----NTQRWQNSLRHNLSFNDCFVKIPRR----PDQ-PGKGSLWALH
ChFoxB      KPPYSYVALCAMAIHSSPAKMMTLSQIYKFIMDNFPFYRK-----NSTRWQNSLRHNLSFNDCFVKVSKT----SEH-GGKGNYWTLH
HvFkh3      KPPYSYVALCAMAIHSSPYQMMTLSDIYKYIMNKFPFYRK-----NNKKWQNSLRHNLSFNDCFVKISKT----SKP-GGKGNYWTMH
SkFoxAB     KPPYSYIALIAMSLENAQDGMLTLNEVYEFIMNKFPYFRE-----NQQRWQNSIRHNLSLNDCFVKIPRA----PGR-AGKGNYWALH
SpFoxAB     KPPYSYIALITMALEGSKDGMMTLNEVYQFIMDKFPYFRE-----NQQRWQNSIRHNLSLNDCFIKVPRA----PGR-PGKGNYWALH
NvFox3      KPPFSYIALITMSIEASPYRMRTLNEIYEFIMTRFPYFRK-----NQQKWQNSIRHNLSLNDCFVKVPRS----FGK-PGKGNYWTLH
BfFoxAB     KPPYSYIALIVMALRSSPLGALPLTGIYEFIERTFPYFRR-----NKRRWQNSIRHNLSLNDCFVKIPRS----CEQ-PGKGGLWALH
HsFoxE1     KPPYSYIALIAMAIAHAPERRLTLGGIYKFITERFPFYRD-----NPKKWQNSIRHNLTLNDCFLKIPRE----AGR-PGKGNYWALD
HsFoxE3     KPPYSYIALIAMALAHAPGRRLTLAAIYRFITERFAFYRD-----SPRKWQNSIRHNLTLNDCFVKVPRE----PGN-PGKGNYWTLD
XlFoxE4     KPPYSYIALIAMAIANSPERKLTLGGIYKFIMERFPFYRE-----NSKKWQNSIRHNLTLNDCFVKIPRE----PGH-PGKGNYWTLD
SkFoxE      KPPYSYIALIAMSIANSPERKLTLGGIYKFIMDRFPFYRD-----NSKKWQNSIRHNLTLNDCFVKLPRE----PGR-PGKGHYWTLD
BfFoxEa     KPPYSYIALISMAIANSPERKLTLGGIYKFIMDRFPYYRD-----RDKKWQNSIRHNLTLNDCFVKIPRE----PGR-PGKGNYWTLD
CiFoxE      KPPYSYIALISMAIASSPERKLTLGHIYKFIMERFPFYRE-----QNKKWQNSIRHNLTLNDCFIKLPRE----PGK-PGKGNYWTLD
NvFoxE      KPPYSYIALICMAITSSPQRQLTLSEIYDFISQRFPFYQT-----CSIKWKNSIRHNLTLNDCFIKLPRE----PNR-PGKGNYWTID
BfFoxEb     KPPYSYVALITMAIVNSPERKTTLAGIYKFIMDHFPYYRE-----ADKKWQNSIRHNLTLNDCFVKLARH----PNR-PGKGSLWALD
BfFoxEc     KPPYSYVALITMAIVNSPERKTTLAGIYKFIMDHFPYYRE-----ADKKWQNSIRHNLTLNDCFVKLARH----PNR-PGKGSLWALD
HsFoxD1     KPPYSYIALITMAILQSPKKRLTLSEICEFISGRFPYYRE-----KFPAWQNSIRHNLSLNDCFVKIPRE----PGN-PGKGNYWTLD
HsFoxD2     KPPYSYIALITMAILQSPKKRLTLSEICEFISGRFPYYRE-----KFPAWQNSIRHNLSLNDCFVKIPRE----PGN-PGKGNYWTLD
HsFoxD3     KPPYSYIALITMAILQSPQKKLTLSGICEFISNRFPYYRE-----KFPAWQNSIRHNLSLNDCFVKIPRE----PGN-PGKGNYWTLD
SpFoxD      KPPYSYIALITMSILQSPQKRLTLSGICEFIMNRFPYYRE-----KFPVWQNSIRHNLSLNDCFVKIPRE----PGN-PGKGNYWTLD
SkFoxD      KPPYSYIALITMAVLQSPQKRLTLSGICEFIMNRFPYYRE-----RFPVWQNSIRHNLSLNDCFVKIPRE----PGN-PGKGNYWTLD
BfFoxD      KPPYSYIALITMSILQSPQKKLTLSQICEFIMNRFPYYRE-----RFPVWQNSIRHNLSLNDCFVKIPRE----PGN-PGKGNYWTLD
CiFoxD      KPPYSYIALITMSILQSPDKKLTLSGICDFIMNRFPYYKE-----KFPAWQNSIRHNLSLNDCFVKIPRE----PGN-PGKGNYWTMD
NvFoxD2     KPPYSYIALITMAILQSPQRKLTLSDICEFIKRRFPYYRE-----KFPSWQNSIRHNLSLNDCFVKMPRE----PGN-PGKGNYWTLD
HsFoxD4     KPPSSYIALITMAILQSPHKRLTLSGICAFISDRFPYYRR-----KFPAWQNSIRHNLSLNDCFVKIPRE----PGR-PGKGNYWSLD
DmFoxD      KPPYSYIALITMAILQSPHKKLTLSGICDFIMSRFPYYKD-----KFPAWQNSIRHNLSLNDCFIKVPRE----PGN-PGKGNFWTLD
CeFoxD      KPPYSYIALIAMSILNSPEKKLTLSEICEFIINKFEYYKE-----KFPAWQNSIRHNLSLNDCFVKVARG----PGN-PGKGNYWALD
SdFoxD      KPPYSYIALIAMAISHSPNKMLTLGEICDYIIHQFTYYHK-----RWPAWQNSIRHNLSLNDCFIKVPRE----YGS-SGKGNFWKLH
NvFox6      KPPYSYIALIAMAIVEAPDKRRTLSEIVEFIKRRFSYYRD-----CIKGWQNSIRHNLSLNDCFIKTWRD----PTN-PSKGHLWTLH
ReFoxL1     KPAYSYIALIAMSIECAPHKRATLSEICQFIRDRFPYYQN-----CKQGWENSIRHNLSLNECFVKQPRE----QGR-PGKGHYWTLD
SdFoxF      KPPYSYIALITLAIMSKAERKATLAEICQYIRETFSYYRE-----NKQGWENSIRHNLSLNQCFQKLPRE----QGK-PGKGHYWVID
HsFoxC1     KPPYSYIALITMAIQNAPDKKITLNGIYQFIMDRFPFYRD-----NKQGWQNSIRHNLSLNECFVKVPRD----DKK-PGKGSYWTLD
HsFoxC2     KPPYSYIALITMAIQNAPEKKITLNGIYQFIMDRFPFYRE-----NKQGWQNSIRHNLSLNECFVKVPRD----DKK-PGKGSYWTLD
SkFoxC      KPPYSYIALIAMAIQNAPEKKVTLNGIYQFIMDRFPFYRE-----NKQGWQNSIRHNLSLNDCFIKVPRD----DKK-PGKGSYWSLD
BfFoxC      KPPYSYIALIAMAIQNAPDKKVTLNGIYQFIMDRFPYYRE-----NKQGWQNSIRHNLSLNECFIKVPRD----DKK-PGKGSYWSLD
SpFoxC      KPPYSYIALIAMAIMNASDKKITLNGIYQFIMDRFPFYRE-----NKQGWQNSIRHNLSLNDCFIKIPRD----DKK-PGKGSYWSLD
DmFoxC      KPPYSYIALIAMAIQNAADKKVTLNGIYQYIMERFPYYRD-----NKQGWQNSIRHNLSLNECFVKVARD----DKK-PGKGSYWTLD
CiFoxC      KPPYSYIALIAMAIQNAPDKKVTLNGIYQWIMERFPFYRE-----NKQGWQNSIRHNLSLNECFVKIPRD----DKK-PGKGSYWTMD
NvFoxC      KPPYSYIALIAMAIQSAPEKRITLSGIYSFIMDRFPYYR------NNQGWQNSIRHNLSLNECFVKVPRD----DKK-PGKGSFWMLD
HsFoxS1     KPPYSYIALIAMAIQSSPGQRATLSGIYRYIMGRFAFYRH-----NRPGWQNSIRHNLSLNECFVKVPRD----DRK-PGKGSYWTLD
DgFoxL1     KPPYSYIALIAMAIQNASDKKVTLNGIYQYIMERFPYYRD-----NKQGWQNSIRHNLSLNECFVKVARD----DKK-PGKGSYWTLD
SpFoxL1     KPPYSYIALIAMAIRNSSDKKVTLNGIYQFIMDRFPYYHD-----NKQGWQNSIRHNLSLNDCFVKVARE----KGK-PGKGNYWTLA
SkFoxL1     KPPYSYIALIAMAIRSAPDQKTTLNGIYQFIMERFPYYHD-----NKQGWQNSIRHNLSLNDCFVKVPRE----KGK-PGKGNYWSLA
BfFoxL1     KPPYSYIALIAMAIRSSPDQKITLNGIYQWIMDRFPYYHD-----NKQGWQNSIRHNLSLNDCFVKDCEE----MFE---NGNYRRRK
```

```
HsFoxL1     KPPYSYIALIAMAIQDAPEQRVTLNGIYQFIMDRFPFYHD-----NRQGWQNSIRHNLSLNDCFVKVPRE----KGR-PGKGSYWTLD
HsFoxI1     RPPYSYSALIAMAIHGAPDKRLTLSQIYQYVADNFPFYNK-----SKAGWQNSIRHNLSLNDCFKKVPRD----EDD-PGKGNYWTLD
DrFoxI3     RPPYSYSALIAMAIHGAPNRRVTLSQIYQYVADNFPFYNK-----SKASWQNSIRHNLSLNDCFMKVPRD----DSD-PGKGNYWTLD
HsFoxI2     RPPYSYSALIAMAIQSAPLRKLTLSQIYQYVAGNFPFYKR-----SKAGWQNSIRHNLSLNDCFKKVPRD----EDD-PGKGNYWTLD
BfFoxI      RPPYSYSALIAMAIQAAPEKKLTLSGIYQYVADNFPFYKK-----SKAGWQNSIRHNLSLNDCFKKVPRD----EDD-PGKGNYWTLD
CiFoxI      RPPYSYSALIAMAIQNSPEKKLTLSQIYQYVAENFPFYKK-----SRAGWQNSIRHNLSLNDCFKKVARD----EDD-PGKGNYWSLD
SkFoxI      RPPYSYSALIAMAIQSAGEKKITLSGIYKYVSDNFPFYKK-----SKAGWQNSIRHNLSLNDCFKKVPRS----EDD-PGKGNYWMLD
SpFoxI      RPPYSYSALIAMAIQSSPDHKITLSGIYRYVAENFPFYKR-----SKAGWQNSIRHNLSLNDCFIKVPRA----DND-PGKGHYWTLD
HsFoxL2     KPPYSYVALIAMAIRESAEKRLTLSGIYQYIIAKFPFYEK-----NKKGWQNSIRHNLSLNECFIKVPRE----GGG-ERKGNYWTLD
NvFoxL2     KPPYSYVALIAMAIRESPEKRLTLNGIYQYIISKFPYYEK-----NKKGWQNSIRHNLSLNECFIKVPRE----GGG-ERKGNYWTLD
BfFoxL2     KPPYSYVALIAMAIKESQEKRLTLSQIYNYIIQKFPYYEK-----NKKGWQNSIRHNLSLNECFIKVPRE----GGG-ERKGNYWTLD
SkFoxL2     KPPYSYVALIAMAIRESQEKRLTLSQIYDFIVNKFPFYEK-----NKKGWQNSIRHNLSLNECFIKIPRE----GGG-ERKGNFWTLD
CiFoxL2     KPPYSYVALIAMAIRDSNEKKLTLSGIYQYIVDKFPFYEK-----NRKGWQNSIRHNLSLNECFVKVPRE----GGG-ERKGNFWMLD
SpFoxL2     KPPFSYVALIAMAIKDSPERKLTLSQIYQYIINKFSYYEK-----NKKGWQNSIRHNLSLNECFLKIARE----GGGGEKKGNYWTLD
SdFoxL2     KPPYSYVALIAMSIAKSPDKRLTLSGIYQYIMDNFPYYAK-----NKKGWQNSIRHNLSLNECFVKVPKE----GGD--RKGNYWTLD
BfFoxG      KPPFSYNALIMMAIRQSPEKRLTLNGIYEFIMKNFPYYRE-----NKQGWQNSIRHNLSLNKCFVKVPRH----YDD-PGKGNYWMLD
HsFoxG1     KPPFSYNALIMMAMRQSPEKRLTLNGIYEFIMKNFPYYRE-----NKQGWQNSIRHNLSLNKCFVKVPRH----YDD-PGKGNYWMLD
SkFoxG      KPPFSYNALIMMAIRQSPEKRLTLNGIYEFIMKHFPYYRE-----NKQGWQNSIRHNLSLNKCFVKVPRH----YDD-PGKGNYWMLD
SpFoxG      KPPFSYNALIMMAIRSSPEKRLTLNGIYEYIMTNFPYYRE-----NKQGWQNSIRHNLSLNKCFVKVPRH----YDD-PGKGNYWMLD
DmSlp2      KPPYSYNALIMMAIRQSSEKRLTLNGIYEYIMTNHPYYRD-----NKQGWQNSIRHNLSLNKCFVKVPRH----YDD-PGKGNYWMLD
CeFkh2      KPPFSYNALIMMAIKDSPEKRLTLAGIYEYIVTNYPFYRD-----NKQGWQNSIRHNLSLNKCFVKVPRN----FDD-PGKGNYWMLD
CiFoxG      KPPYSYNALIMMAIKKSPRKRLTLSQIYQYITTTFPYYKE-----NKQAWQNSIRHNLSSNKCFVKVPRH----YDD-PGKGNYWMLD
DmSlp1      KPPYSYNALIMMAIQDSPEQRLTLNGIYQYLINRFPYFKA-----NKRGWQNSIRHNLSLNKCFTKIPRS----YDD-PGKGNYWILD
MlFoxG      KPLFSYNALIAMAISQSPLKKLTLSEIYDFIIETFPYYRD-----NKKGWQNSIRHNLSLNKCFVKVPRH----YND-PGKGNYWMLN
NvFox1      KPPYSYVALISMAIKQSPGRKITLNGIYHFITSAFPYYTQ-----NKRGWQNSIRHNLSLNRCFVKVHRE----KAD-PGKGCYWTLD
NvFox5      KPPYSYVALISMAIKQSKGQKITLSGIYQFIIENFPYYRL-----NKRGWQNSIRHNLSLNKCFVKIPRE----RSD-PGKGCYWALD
NvFoxD1     RPPYSYIALIAMAVQNSPEKRLTLDGICKFIRDRFPFYRE-----TYPSWKICIRNNLSLNDCFIKTG-I----KSDEPLKGNYWTLD
SdFox1      RPPFSYITLISMAVKNAPTKKLTLNEIYSYIMDHFPFYRE-----NRRGWQNSIRHNLSLNECFVKVPRD----KDDPPGKGNYWTLA
SkFoxJ1     KPPYSYATLICMAMKETKKNKITLSAIYKWIQDNFMYYKV-----AEPSWQNSIRHNLSLNKCFTKVPRR----KDE-PGKGGFWKID
BfFoxJ1     KPPYSYATLICMAMKETKKSKITLSDIYKWIKTNFKYYEM-----AEPSWQNSIRHNLSLNKCFTKVPRS----KNE-PGKGGFWKID
SpFoxJ1     KPPYSYSTLIWMAMKESKKHKITLSSIYKWITENFKYYQV-----ADPSWQNSIRHNLSLNKCFQKVPRK----KDE-PGKGGFWRID
HsFoxJ1     KPPYSYATLICMAMQASKATKITLSAIYKWITDNFCYFRH-----ADPTWQNSIRHNLSLNKCFIKVPRE----KDE-PGKGGFWRID
Hvfkh2      KPPYSYAALIIMAMKSKVCGKMTLSEIYKWIGDHFPFYKY-----AEPSWQNSIRHNLSLNKCFTKIPRN----KGD-PGKGGYWTVI
ReFoxJ      ------TTIIYLAIRSSKNDKVTLGEIYQWIKDHFMYYRV-----AEPTWQNSVRHNLSLN--------------------------
SkFoxJ2     KPPYSYANLITFAINSSPKKKMTLSEIYQWICENFPFYRE-----AGNGWKNSIRHNLSLNKCFLKVPRS----KDD-PGKGSYWAID
SpFoxJ2     KPPYSYANLITFAINSSPKKKMTLSEIYQWICENFPYYRE-----AGNGWKNSIRHNLSLNKCFMKVPRS----KDD-PGKGSYWAID
BfFoxJ23    KPPYSYANLITFAINSSPKKKMTLSEIYQWICDNFPYYRD-----AGNGWKNSIRHNLSLNKCFLKVPRS----KDD-PGKGSYWAID
HsFoxJ3     KPPYSYASLITFAINSSPKKKMTLSEIYQWICDNFPYYRE-----AGSGWKNSIRHNLSLNKCFLKVPRS----KDD-PGKGSYWAID
CiFoxJ2     KPPYSYASLISLAINSSNEKKMTLSEIYQWICKTFPYYSG-----AGTGWKNSIRHNLSLNKCFMKVPRA----KDD-PGKGSYWAID
HsFoxJ2     KPRYSYATLITYAINSSPAKKMTLSEIYRWICDNFPYYKN-----AGIGWKNSIRHNLSLNKCFRKVPRP----RDD-PGKGSYWTID
HsFoxK1     KPPFSYAQLIVQAISSAQDRQLTLSGIYAHITKHYPYYRT-----ADKGWQNSIRHNLSLNRYFIKVPRS----QEE-PGKGSFWRID
BfFoxK      KPPYSYAQLIVQAITSANDKQLTLSGIYAHITKNYPYYRT-----ADKGWQNSIRHNLSLNRYFIKVPRS----QEE-PGKGSFWRID
HsFoxK2     KPPYSYAQLIVQAITMAPDKQLTLNGIYTHITKNYPYYRT-----ADKGWQNSIRHNLSLNRYFIKVPRS----QEE-PGKGSFWRID
SkFoxK      KPPYSYAQLIVQAIISAQDKQLTLSGIYCHIMKNYPYYRS-----ADKGWQNSIRHNLSLNRYFIKVPRS----QEE-PGKGSFWRLD
SpFoxK      KPPYSYAQLIVQAIMSAQDKQLTLSGIYSYITKTYPYYRT-----ADKGWQNSIRHNLSLNRYFIKVPRS----QEE-PGKGSFWRLD
CiFoxK      KPPYSYAQLIIQAISSAPHRQLTLSGIYAHITKNYPYYRT-----ADKGWQNSIRHNLSLNRYFVKVPRS----QEE-SGKGSFWKVD
DmFoxK      KPPYSYAQLIVQAISAAPDKQLTLSGIYSFIVKHYPYYRE-----TNKGWQNSIRHNLSLNRYFIKVARS----QDE-PGKGSFWRID
HmFoxK      KPPYSYAQLIVQAITSSADKQLTLNGIYQFIMKNYPYYRI-----NDKGW-------------------------------------
SccFox2     KPPHSYATMITQAILSSPEGVISLADIYKYISSNYAYYRF-----AKSGWQNSIRHNLSLNKAFEKVPRR----PNE-PGKGMKWRIS
DebFkh      KPPYSYATMITQAILSNDDGVMSLSEIYNWIASHYAYYKY-----SKTGWQNSIRHNLSLNKAFEKVPRR----PNE-PGKGMKWQIS
SchFox2     KPPYSYSVMIAQAILSSSECMMTLSNIYSWISTHYPYYRT-----TKSGWQNSIRHNLSLNKAFRKVPRK----SGE-QGKGMKWSIV
AspFkh2     KPPYSYATLIAQAIFSSEEEKLTLNSIYNWIMDKYAFYRH-----SQSGWQNSIRHNLSLNKAFQKVPRR----TDE-PGKGMKWQIA
DrFoxQ2     KPAQSYIALISMAILDSDEKKLLLCDIYQWIMDHYPYFKS-----KDKNWRNSVRHNLSLNECFIKAGRS----DN---GKGHFWAIH
ChFoxQ2a    KPTQSYIALIATAILKSKDKRLVLSDIYKYILDNYSYFQS-----QDKSWRNSIRHNLSLNECFIKVGRS----E----GKGHYWAIH
SkFoxQ2-2   KPTHSYIALISMAILSTSERKMLLSEIYKYIMNNFPYYRN-----KEKSWRNSVRHNLSLNECFIKNGRS----YN---GKGNYWSIH
NvFox4      KPAHSYIALIAMAILSNSSKKMILGDIYQYISDNFPYYRN-----KDKSWRNSIRHNLSLNECFITAGRS-----E---GKGNYWAIH
SkFoxQ2-1   KPTESYIALIAKAILSVREQKMLLCDIYQNIMDIYPFYRN-----NDKSWRNSIRHNLSLNECFIKNGRS----ND---GRGNYWSIH
SpFoxQ2     KPPHSYIALIAMAIINSQDKHLLLCDIYEYIMKRFPFFKD-----NERSWRNSIRHNLSLNECFIKAGRS----GD---GRGHFWAIH
BfFoxQ2a    KPRHSYIALIAMAIMSSKDKRLLLGDIYQWIMDNFPFYRN-----NERSWRNSIRHNLSLNDCFIKAGRS----QD---GKGNYWAIH
BfFoxQ2b    KPPLSYIALIAKAILGSPAKRLSLGSIYQYITDNYPYYQN-----RGQGWRNSVRHNLSLNDCFIKAGRC----ED---GKGNYWAIH
SkFoxQ2Dol  KPSHSYIGLIAMAILKSKDRKMVLSDIYQYILDNYPYFRA-----RGPGWRNSIRHNLSLNDCFVKAGRS----AN---GKGHYWAIH
DmFoxQ2     KPQHSYIGLIAMAILSSTDMKLVLSDIYQYILDNYPYFRS-----RGPGWRNSIRHNLSLNDCFIKSGRS----A----GKGHYWAIH
BfFoxQ2c    KPSHSYIGLIAMAIMSSKEKKLVLSDIYKYILDNYPYFRN-----RGPGWRNSIRHNLSLNDCFVKMGRS----AN---GKGHFWAVH
CeFkh10     KPQHSYIGLIAMAILSSPQKKMVLAEVYEWIMNEYPYFRS-----RGAGWRNSIRHNLSLNDCFVKAGRA----A----GKGHYWAVH
ChFoxQ2b    KPNHSYISLIANAILSSKEKRLVLSDIYQFVLDTQPYFRK-----AGQGWRNSIRHNLSLNECFVKAGRS----P----GKGHFWAIN
HmFoxQ2b    KPSHSYISLIANAILASPDKRLVLSDIYKYVLERYDYFKK-----KGSGWRNSIRHNLSLNDCFIKAGRS----PN---GKGHYWAIN
NvFox2      KPSQSYIGLIGKAIMSVPQKKLVLSDIYNYILTHYPYFRN-----KGAGWRNSIRHNLSLNECFVKVGRS----S----GKGHFWAIN
HmFoxQ2a    RNTPSYTAIIAQAILSSKEKKLPLGDVYEYIAENFPEFLK-----KGQGWRNCVRHNLSLSECFVKAGRA----RN---GRGNYWGIH
HmFoxQ2c    EKIASYTEMIAKAIFSGKGNMSTLQDIYEFLIENFPILKS-----RGKSWKNSVRHTLSLNEWFVKIPRT----DN---GKSCYWSIH
SpFoxY      LPGFTYAELITMAIQSSPSRMMTIVDIQQFFRDRFPCFRT-----SYKGWHNSIRHNLSARECFYKVPIV----DKRHKCRTHYWMIN
HsFoxP1     RPPFTYASLIRQAILESPEKQLTLNEIYNWFTRMFAYFRR-----NAATWKNAVRHNLSLHKCFVRVENV----KGAV------WTVD
BfFoxP      RPPFTYASLIRQAIIESPEKQLTLNEIYNWFTRTFAYFRR-----NAATWKNAVRHNLSLHKCFVRVENV----KGAV------WTVD
HsFoxP4     RPPFTYASLIRQAILETPDRQLTLNEIYNWFTRMFAYFRR-----NTATWKNAVRHNLSLHKCFVRVENV----KGAV------WTVD
SkFoxP      RPPFTYAALIRQSIIDSPDGQLTLNEIYNWFTRTFAYFRR-----NAATWKNAVRHNLSLHKCFVRVENV----KGAV------WTVD
SpFoxP      RPPFTYAALIRQGIIDAPDRQLTLNEIYNWFTRTFAYFRR-----NAATWKNAVRHNLSLHKCFVRVENV----KGAV------WTVD
HsFoxP2     RPPFTYATLIRQAIMESSDRQLTLNEIYSWFTRTFAYFRR-----NAATWKNAVRHNLSLHKCFVRVENV----KGAV------WTVD
DmFoxP      RPPFTYASLIRQAIIDSPDKQLTLNEIYNWFQNTFCYFRR-----NAATWKNAVRHNLSLHKCFMRVENV----KGAV------WTVD
CiFoxP      RPPFTYASLIRQAVLEAPDHQMTLNEIYNWFQKKFAYFRR-----NAPTWKNAVRHNLSLHKCFVRVENV----KGAV------WTVD
CeFkh7      RPPYTYASLIRQAIMESSDCQLTLNEIYTWFTETFAYFRR-----NAATWKNAVRHNLSLHKCFQRVEQV----KGAV------WTVD
SdFoxP      RPPFTYASLIRQAILESSDQCLTLCEIYAWFMKNFVYFRD-----NNPTWKNAIRHNLSLHKCFVRVELS----RGAV------WTVD
HsFoxP3     RPPFTYATLIRWAILEAPEKQRTLNEIYHWFTRMFAFFRN-----HPATWKNAIRHNLSLHKCFVRVESE----KGAV------WTVD
SpFoxX      RPPHSYVRIVIMSLLDCPGHEATIREIYDMITYKFPYYQE-----NRLHWKNSVRHNLTVFSCFERVITE----EGSTSRGSNRWRLI
HsFoxN3     KPPYSFSCLIFMAIEDSPTKRLPVKDIYNWILEHFPYFAN-----APTGWKNSVRHNLSLNKCFKKVDKE----RSQSIGKGSLWCID
BfFoxN23    KPPYSFSCLIFMAIEDSPSKRLPVKEIYNWILEHFPYFVN-----APTGWKNSVRHNLSLNKCFKKVEKE----KGQSIGKGSLWMID
SkFoxN23    KPPFSFSCLIFMAVEDSPNKRLPVKDIYQWILDHFPYFQN-----APTGWKNSVRHNLSLNKCFKKVEKE----KGQTIGKGSLWCID
SpFoxN23    KPPFSFSCLIFMSIEDCPLKRLPVKEIYRYIQDHFPYFRT-----APTGWKNSVRHNLSLNKCFRKVDKI----KGQSLGKGSLWCVD
HsFoxN2     KPPYSFSLLIYMAIEHSPNKCLPVKEIYSWILDHFPYFAT-----APTGWKNSVRHNLSLNKCFQKVERS----HGKVNGKGSLWCVD
CiFoxN      KPPYSFSCLIFMAVEDSVEKRLPVKEIYSWVCKHFPYFKT-----APSGWKNSIRHNLSLNRCFKKAEIP----NKRKEVKGSLWCID
DmFoxN23    KPPYSFSSLIFMAIEGSNEKALPVKEIYAWIVQHFPYFKT-----APNGWKNSVRHNLSLNKSFVKVEKA----PNMG--KGSLWRVE
NvFoxNx     KPPYSFSSLIFMAIEESPNKRLPVKDIYNWIMDHFPYFRD-----ARLGWKNSVRHNLSLNKCFKKVDKD----KGQVRRSFDMSLID
HsFoxR1     RPPLNYFHLIALALRNSSPCGLNVQQIYSFTRKHFPFFRT-----APEGWKNTVRHNLCFRDSFEKVPVSMQ-GGASTRPRSCLWKLT
HsFoxR2     RPPLNCSHLIALALRNNPHCGLSVQEIYNFTRQHFPFFWT-----APDGWKSTIHYNLCFLDSFEKVPDSLK-DEDNARPRSCLWKLT
```
