# Supplementary material for: The Fox/Forkhead transcription factor family of the hemichordate Saccoglossus kowalevskii
Source: EvoDevo. 2014 May 7;5:17. doi: 10.1186/2041-9139-5-17 (PMC4077281; doi:10.1186/2041-9139-5-17)
Supplement: Additional file 5: Table S5 — EH I-like motif in the FoxQ2 family. [file 2041-9139-5-17-S5.pdf]

Additional Table 5. EH I-like motif in the FoxQ2 family

<table>
<tr><th colspan="2">EH I-like motif in the foxQ2 family</th></tr>
<tr><th>Protein name</th><th>Sequence with EH I-like motif</th></tr>
<tr><td>Hydra magnipapillata
HmFoxQ2a</td><td>MQMTISMEEKDKKTVENKNGKKVSYFSIDQILSKNLYEVEEKSIKVNANNDNNINSSIDKLNECVKSEDLIEKKEIRNTPSY
TAIIAQAILSSKEKKLPLGDVYEYIAENFPEFLKKGQGWRNCVRHNLSLSECFVKAGRARNGRGNYWGIHPRYIKNFSLGD
YRKRRASHRQRSFGPSFVNCSYQDNHTNQYIFEHKNFLSKNFTMESILTPEHGKHRIPFYLLNTAYNENILRENSNHFHQAY
SLRVPIRNYSLEPCIPKLSSNQNMNYVSEVNCSCSKCPILNTINRNGLI</td></tr>
<tr><td>Hydra magnipapillata
HmFoxQ2b</td><td>MIEEESKLIISRASVSTDYNSDDDSYPTLIMNKHSSSFNEEHRINNELFNNKPSHSYISLIANAILASPDKRLVLSDIYKYVLER
YDYFKKKGSGWRNSIRHNLSLNDCFIKAGRSPNGKGHYWAINTANYEDFARGDFRRRRVQRRVRRGVSSPTYPPYAINYYP
FQLHYPQNKGFTDNSGYILEKDILCSIEKYSFEKKEEPREFFPRELAEAKSQTSSAKTSHSVKSNRPFDIENLLKDDKITKRKY
SIEPSRIYFERPYLY</td></tr>
<tr><td>Hydra magnipapillata
HmFoxQ2c</td><td>MSLNQFAEHKPLFSEQKPQIKHSVAEITSPFNEDNSSQIDKDVKNVFKDELLENDLSCNKKVEPEKIASYTEMIAKAIFSGKG
NMSTLQDIYEFLIENFPILKSRGKSWKNSVRHTLSLNEWFVKIPRTDNGKSCYWSIHPIYLNRFRKGDFQKQRKSGITRLHHS
SKRTHVFPSYFYTESTPSDYPHMVPTLPSPYREHCPYVSNPTLPNLSLQYYPSAAIPNRSHTDSDSQSYIKTDKTDIKPYSLKS
PSSYFFSPYQKYPQDQLSPCGAQKMYMESLRQSPMMHFPQTFSPSYGNTYSGDNNVKSSIETRNMYFPTDYRTCHVTDFP
YAPYTPY</td></tr>
<tr><td>Nematostella vectensis
NvFoxQ2b</td><td>MANSGYSAFKPVGNYILESKHDKKQRCYDRDFCCTVERPGLLTIPDDYTCWKVGPMWPFWSPIPTQFLRFNSFVTYSHEKP
NQSYISLISEAILSSPEQKLILSDIYNFILTRYPYFRTKGTGWRNSIRHNLSLNECFVKAGRSPNGKGHFWAIDATYFDDFRRG
DFRKRRSYRRYKSKISNRGRVDDAFEGKVELLKVDRECGDHKRDDISWDGYKRQAQDSTKQALLGYGDHKSDYIALGVN
EGQVKTRTDEEEILLSNNVLTTRYKMQRVVSSTRQVIPIMIADKPKYDTSTTPVMAKRKKSFDVESLLAP</td></tr>
<tr><td>Nematostella vectensis
NvFoxQ2c</td><td>MHRTTAAIDKVTERALIMSQYFQLPARTQKFGYPDSCYDDVRFLHRPAEGLSPPLKRNCDSPVSTAREETRIKIEELERSEEE
NGIVVLKKVESSEEDSQVTEDDDMKQAEDHKEEDEAAKDDDKNAETFVAVIAQSILSVPTKRMTLSSIYSFIAKNYPHFDK
EKGPGWRNSVRHNLSSNDCFVKASRAENGKGHYWMIHPKDLPEFSKGNFRRRRKPRRPKCSHSLMFRDAPLYYHSYGLG
YSPYAYAPLRASQEPVESGLPLPPYGSTERALAHRLRARPGLLSAYNDRVGMLPYPYSYPSMLSYRGLGEAGRTTYPGLGG
HHNSSFHPVHQHSCSCHR</td></tr>
<tr><td>Nematostella vectensis
NvFoxQ2a / NvFox2</td><td>MMAFACLPSKEKSSAFTPFRMETTGNNCVQEISSPMPFYQTHSPPGSPHFLPFPPCYATQFYTEFSYIHRREESKMQANITNS
FFHSSVFGYHVTEEEKPSQSYIGLIGKAIMSVPQKKLVLSDIYNYILTHYPYFRNKGAGWRNSIRHNLSLNECFVKVGRSSN
GKGHFWAINPENYEDFSKGEYRRKRVSKKRTASTDGVARTSEKDRLVEKKPDEQVTCTKRARKQDQCKELLTGLLTTAKF
GFPKICESKPTEYRGFHIENILSGIKTEKRV*</td></tr>
<tr><td>Nematostella vectensis
NvFox4
fgenesh1_pg.scaffold_2180000
20</td><td>MEECAPESPLRNDYDYEELGENQQEPASDDGSDTESDALNTDCNENARERDTPEVVTDVDAEKSVSHLVKKETIVDDDAD
VKPAHSYIALIAMAILSNSSKKMILGDIYQYISDNFPYYRNKDKSWRNSIRHNLSLNECFIKAGRSENGKGNYWAIHPANLE
DFANGDFRRRRARRRVRKSNALKYGVSAGYPYFRSFTPYTPYSSYLAYRADDLTRVFNGYPSSYQLPQVSVARKSFAIDDL
MSTTPQYNQDALHKPEYSDSSAAVAVSNSYSGLTSPGHIYAGIYGSSSCGYSCLSSLAGYSSAERREKESWQDTLCKLQEQL
RKTSHL*</td></tr>
<tr><td>Patiria miniata
FoxQ2

gi|313906881|gb|ADR83539.1|</td><td>MTLFTIDKITEKAAPRPAAHHHHARRFQPYHPYPTVSDAAPVGYPAYLGKLAHSSPLPVTGPSGQRRDECLNSTGGSPSVSP
GGYSAVSSSEAGSPEPEKAPSKVTDKNNNKKSDTDEHKKPPHSYISLIAMAILASPEKRLLLCDIYQYIQENYPYYRNNDRS
WRNSIRHNLSLNECFIKYGRSGDGRGNFWAVHPANVEDFSRGDFHRRRARRRVRASDMMLHGYSVYHPYAAPSTPAHAYP
VPVAATPLGFVPMTVTTLPPYPVSPAVYPPSSYPAAGYPVPTLNPLPPMTSASTSPVSTQRSSPLNLAIPGKSSPVFTTSYEHA
PVSLPSSYPSTQPSYVYPPSTTQSPFHYSPPTSRDSPLARYPPTTLGLAPAPGYGSPLARYPPTTLGLAPAPGYGYPAVSQSVIT
SSLGSYQTESAIPRFLH</td></tr>
<tr><td>Saccoglossus kowalevskii

FoxQ2-1</td><td>MTLFSVQSLTDNHQASQTTQTQPISPGCFSTTSSSDASSLYAGSPVSDTSTSDASSKKPTESYIALIAKAILSVREQKMLLCDI
YQNIMDIYPFYRNNDKSWRNSIRHNLSLNECFIKNGRSNDGRGNYWSIHPANLEDFVKGDFRRRKARRRVRQCYDMVNAV
YCHHPFAAAAAYGPSGYVPMTASSLPYNYFSHPSFTTPMYPASPSMYSPSHQYTTPAAAVTTTPSMYSPSHQYTAPTTTSLP
VSPEPVPSLSSYCSQPLPVSPPQTPSMPPMMSAASWQDTFNRLQAMK</td></tr>
<tr><td>Saccoglossus kowalevskii

FoxQ2-2</td><td>MFENIEDMGDLITSDFCLKKNSLPVSEFNHVVTEEKDTEDLFDNSENIARVLLSLDRSKSKPHSSEVFTSSSRKQTNSDSNQS
SKPTHSYIALISMAILSTSERKMLLSEIYKYIMNNFPYYRNKEKSWRNSVRHNLSLNECFIKNGRSYNGKGNYWSIHAACEE
DFAKGDFRRRRARRRVRKCHRDEELVAMRTSYGYSGYLSMSAPDPYYSISAPCNAFVPMSYPSPSMLPRGLFNVDSFLQRN
DAFPHPLYAPFTTRTASISAPSNTGLTSSQTDLFGSNSQLPSWQDTLSRLPTNVIG</td></tr>
<tr><td>Saccoglossus kowalevskii

FoxQ2-3</td><td>MCSTSSSSLIPYEFKSTEQPTMYAAWGGSGMTQAAFEFHRAQMYNYNSRLRYAGIPGIPTATAHGISPYLHHHPHGADTFTA
LMAFNKVDPRARLIHEEPKPSHSYIGLIAMAILKSKDRKMVLSDIYQYILDNYPYFRARGPGWRNSIRHNLSLNDCFVKAG
RSANGKGHYWAIHPANIDDFTKGDFRRRRAQRKVRKHMGLSVPDDEDSPSPPPVTSAQMKWVNPSFLHSGVSSIGENAATI
TGHLHQPTKKRLFDVESLLAPETNENKDKSADEDDEVEDCKDTLEHDLKAAQIERNQPNTETAERISEFKSEERSSPNTETC
HSPTAKNLRQSPQTNLRSSWTAPTLPPRTNGWPGSAWSIVTPSTRTTYSAGITPIPYGTPTPSPGMDTVQQWQETFSRIMAKS
YSKNLQVES</td></tr>
<tr><td>Branchiostoma floridae

BfFoxQ2c</td><td>MHHFVRPVEMMPYHVKPSSGLQAPSSREGLQTIAVPRPQLPYQGVCVYDCGGRAHPSPPVHLPSDARLPMYASNPFFSTSY
LFYPRSYLPVGPGTSFGPIPHEEPKPSHSYIGLIAMAIMSSKEKKLVLSDIYKYILDNYPYFRNRGPGWRNSIRHNLSLNDCFV
KMGRSANGKGHFWAVHPANVDDFAQGDFRRRRAQRKVRKHMGLLPEDDGNSSSGSSNENSGTVSPPPRPCPSDPPGGSSH
DSVPPLTGREESTVLQTSFSSDMSCQEKAREKTAKISPDGTLVRRKFDMASLLEPDSQ</td></tr>
<tr><td>Fugu rubripes
FoxQ2
GSTENP00004847001</td><td>MTKEDTSSRTIGRERLGLSFTIDYLLFNKGVKGSREEATGSRTAEQTASSMLNHQNPKTKEVEIRAKTQENRLQRSETRERK
SEGEEGDEDQRAKRGEEVTTTALTCSGPENSADKPNQSYIALISKAILASEQKKLLLCDIYQWIMDHFPYFKSKDKNWRNS
VRHNLSLNDCFIKAGRSDNGKGHFWAIHPSNYQDFSNGDYHCRRARR</td></tr>
<tr><td>Lottia gigantea
FoxQ2-1

>jgi|Lotgi1|152124|
fgenesh2_pg.C_sca_1000262</td><td>MCSSNSGTILPYGLKIPTPSPYFPFAPGVGNLPLDVQRSQLCDYAARLQYGLRGYPGLGISPYHAHLPDPYTQAYLYKHDLR
ARYIQEEPKPSQSYIGLISMAILGSKDKKLILSDIYQWILDNYAYFRTRGPGWRNSIRHNLSLNDCFIKSGRSANGKGHYWAI
HPANLDDFSRGDFRRRRAQRKVRKHMGLAVPDDEDSPSPSPSSTPVNWQGSYQDDKVDEIKLSEVVENTSKIETGCSPATG
TGNGPPAKKRLFDMESILAPDNHRGSQITLQNENNNLDDGESEIEIDTDRETVDVCEIDSQHSNEEAPLSPNSTPSSPMEDGT
LSPRVHSQDDAEPGEIKPESFSSTSKSELWSIGNVLHGRPSASHHHLPTQAPWDAYATYIYPMLSSSYSVVLPGTTSISAEAAS
RWREAMYGTSSQRPKSPKEEKSE*</td></tr>
</table>

EH I-like motif in the foxQ2 family	
**Protein name**	**Sequence with EH I-like motif**
Lottia gigantea<br>FoxQ2-2<br>>jgi|Lotgi1|156566| fgenesh2_pg.C_sca_11000252	MRNFSIEFLTSKPTEKSPESRNQKIVMNNSLDSSWESSSSTSSPESSPSSVNKRFNSDAYMVNGIPGMSAYLTQNFAHIQQNA<br>EVLGSIPLNAGAIHYGSSNETTRQQEVANMAGKAISRSKQNSSEISMVKEPSKTKPSHSYIALISMAILESSEKKLLLGDIYEY<br>IMEKFPYFNNQEKAWRNSIRHNLSLNECFIKNGRSDNGKGNFWSIHPACLEDFSKGDFRRRQARRARRTLPQMPARNPDI<br>VYGTMGYVPMASSTLPLNSAAPQLGAYKPQVPMMSSSLPQMTNQIPQVSMTSRNLPSPTNESPKVTMTSGHLFSPTSLLPQ<br>ISMTPGNQLPQVSMTSGHLLSPTNPGAAYFSYPNNMNCFINQTSLVPNQFQSW*
Lottia gigantea<br>FoxQ2-3<br>>jgi|Lotgi1|152276| fgenesh2_pg.C_sca_1000414	MHSFSIDYLTRKEADDGNRSPARSLNSFGESNSPKDCGNISERSADGDQEPTYGSSKPPLSYIALISMAILDSADKQTLLGDIY<br>QFIMDKFPYYNNKGKAWRNSIRHNLSLNECFIKSGRAENGRGNFWSIHPACIEDFSKGDFRRRKARRRARNSGLLDISEMPL<br>SYRCNLGYVLMTPSVVPFSPVKQSSSEVTTYSHPETPFPSIYQQSPESFNSW*
Lottia gigantea<br>FoxQ2-4<br>>jgi|Lotgi1|132928| e_gw1.81.105.1 FoxQ2-4	MKKNYDGVLNLKKPEGSRSGFIPASLRKLSVKKPNMSYIGLISMAIQSSSSKKMLLSEIYRWIVNNFPYYKMSDRSWRNSV<br>RHNLSLNECFIKCGRSDNGKSHYWTIHPANMNCFSHGDFRRRHARQLVKRCDADLERLCGKPGDERLEYGGVITSKSRYV<br>PMTCTIVNDDYIRTQFGEEVLFTQKPVHHSIKSEVESSDDSS
Nasonia vitripennis<br>FoxQ2	MCNNESPPPSKETLAVGLSVGNSMTNFLPGIEHYRLQLYHYAMAERLRLAQQLHSQHSQHAVCQSASIAQSGTGQLGINTS<br>FPLPLYSTGSSYSRGLALSMALLQPHHQQVPEEPKPQHSYIGLIAMAILSSPEKKLVLSDIYQHILEHYPYFRRRGPGWRNSIR<br>HNLSLNDCFVKSGRSANGKGHYWAIHPANLEDFRRGDFRRRKAQRKVRRHMGLAVDEEPDSPSPPPLPATPPPLSTLGPPH<br>LSQPPTSGIWSPQNQHQQIHHLNRSFTTHQFQNSSLQPARKRQFDVASLLAPDDPYIKTSLQLSKSRQISCSEDEPENEPDDG<br>DIEIDVVAEPERKKVHSTLKLINSNCWRDAPLPSAEREHDSMVQLHDLHLSSYYMPANTNSTASSM
Pediculus humanus corporis<br>FoxQ2	MCSNQSPVPSPNMAPTTPVLLIQFRELQLYNYAVVERLRFSNFGHYNNQLPFCPGYPYNPRLALQMSLFHNRVFQPEEPKPQ<br>HSYIGLIAMAILSSPEGKLVLSDIYQYILDNYPYFRSRGPGWRNSIRHNLSLNDCFVKAGRSANGKGHYWAIHPANVEDFKK<br>GDFRRRKAQRKVRKHMGLAVDEEPDDSPSPPPVPITPPPPRVPIPGLCSWPTVGSSLGLTTSSLFPPIQNLTRKRQFDVASLLA<br>PDEEEEEVNVKHPRKYEDGYLSSDGDEIEGDDDIDVVNEDLAAVANSKEVSQSCSKLNRTGRNRIKNDLPDSPNQRLFVKD<br>LSIIENRQTSTPSPALSTSFNKEEETSPLRVSPIESNFSNETQRRSPQLPFWIRQTGIIHPMAMLPTGSLSFMEQQQHIVNKIYEQ<br>RLIAAQQHHQQKLLQQQNSLSGELSPINLKNEET
Oryzias latipes<br>FoxQ2	IAMENRHSHNGSKDRLGLSFTIDYLLFNRVKGSKDQTTGSSAAEQTNSSTLNDQNPTLKEVDKRLILSEEDSVRHKASEEEE<br>NEDHGEQKVTLAPPSTNPEKSADKPNQSYIALISRAILSSKEKKLLLCDIYQWIMDHYPYFKSKDKNWRNSVRHNLSLNEC<br>FIKAGRSDNGKGHFWAIHPGNYQDFSKGDYHCRRARR
Ornithorhynchus anatinus<br>FoxQ2	MAASRGGRPLGLSFTIDRLLFDQEKPSEGGGAPEAQLQECPEGKGQAGGEQSEPGGQASLRKPGQSYVALISTAILASPKRK<br>LLLSDIYQWIMDTYPYFKNQEKSWRNSIRHNLSLNECFVKAGRSDSGKGHFWTIHPANLEDFAKGEYHRQRARSQLRRMA<br>VNLRLCQPRTFYGLRGCPGRYSLCRCPLLSSGLTDVGPPAQVALPALPLSPPHPYHPVGPEFPQGEAEPLPAASDAARSHPFR<br>PKGYPANGGSEALRGPCEVASGDLGWAGVHSPSDCCRWMGDERNKPNGLLNPFPTPSGPLRPGTPITSNSGERTPTGKQFS<br>PLGFGFLTSPNGHHLIVVASPRAWKHLVRPTPSESRKVISPISLAPGAGERTGAGLEQDEETANERKWKTRETQQSINAETNT<br>SLTVQCCIPASEGWLDRSGPPATLNPGFSLRGRRARDGNRSRPSRFHPGGSGGREPGGGSRKWEMRRLPTETPGRARPKQK<br>EA

Additional Table 5. EH I-like motif in the FoxQ2 family

The table shows FoxQ2 proteins and their identified/ predicted EH-I like motifs. Fork-head domain highlighted in green, N-terminal EH-I like motif highlighted in yellow, C-terminal EH-I like motif highlighted in blue.
