# Supplementary figures and images for: The Fox/Forkhead transcription factor family of the hemichordate Saccoglossus kowalevskii
Source: EvoDevo. 2014 May 7;5:17. doi: 10.1186/2041-9139-5-17 (PMC4077281; doi:10.1186/2041-9139-5-17)

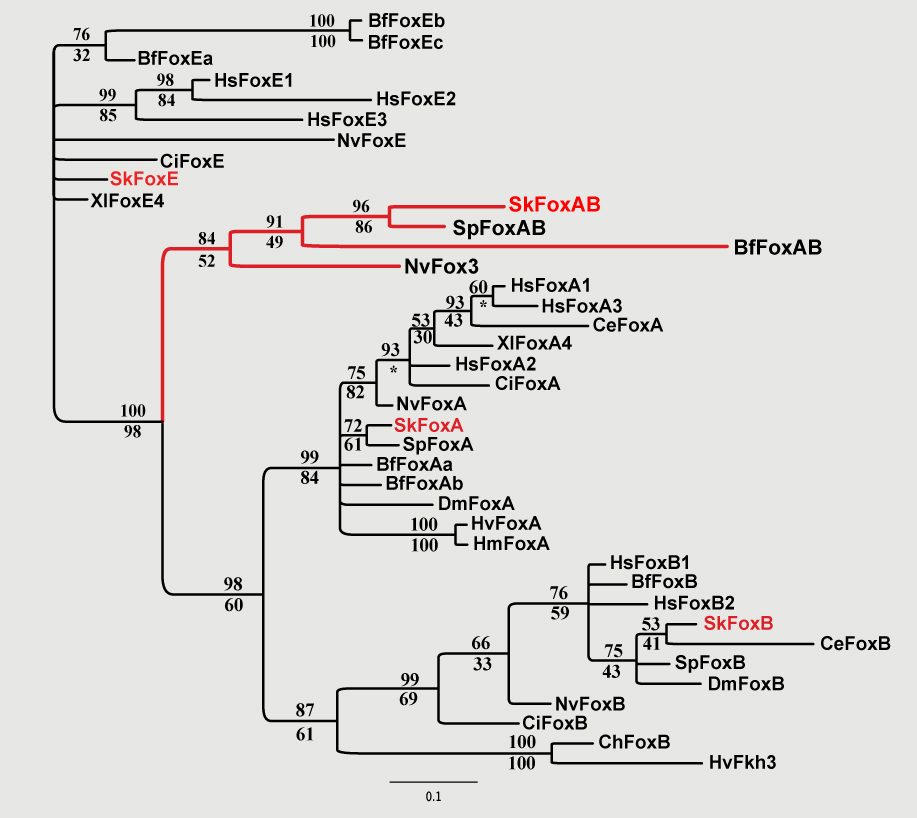

Supplement: Additional file 7: Figure S1 — Phylogenetic analysis of the FoxAB family. S. kowalevskii FoxAB groups together with previously found members of this new Fox gene family supporting the idea that this new family is a separate ancestral Fox family. Bayesian analysis was performed using the mixed amino acid substitution model applying four independent simultaneous Metropolis-coupled Markov Chains Monte Carlo in two independent simultaneous runs. The likelihood model was set to gamma rates = 4. A tree was sampled every 250 generations for two million generations. The first 25% of the sampled trees were excluded via ‘burnin’ prior to consensus tree calculation. Xenopus laevis FoxE4 was used as outgroup. The trees converged to a standard deviation of 0.0071. Maximum likelihood analysis was performed using the Le-Gascuel (LG) amino acid substitution model [101] with estimated proportion of invariable sites and gamma shape parameters. The number of substitution rate categories was set to 4. Starting tree was computed with BIONJ and 1,000 bootstraps were performed. The input alignment comprises 39 sequences with 78 characters (see Additional file 8: Table S7). For sequence accession numbers see Additional file 1: Table S1 and Additional file 3: Table S3. Baysian posterior probabilities are displayed on top of each branch and maximum likelihood values underneath each branch. Stars indicate differing tree topologies which lead to no support value at that position. Branches with posterior probabilities below 50% are condensed. Abbreviations: Hs: Homo sapiens; Bf: Branchiostoma floridae; Nv: Nematostella vectensis, Ci: Ciona intestinalis; Sk: Saccoglossus kowalevskii; Xl: Xenopus laevis; Sp: Strongylocentrotus purpuratus; Ce: Caenorhabditis elegans; Dm: Drosophila melanogaster; Hv: Hydra vulgaris; Hm: Hydra magnipapillata; Ch: Clytia hemisphaerica. [file 2041-9139-5-17-S7.jpeg]

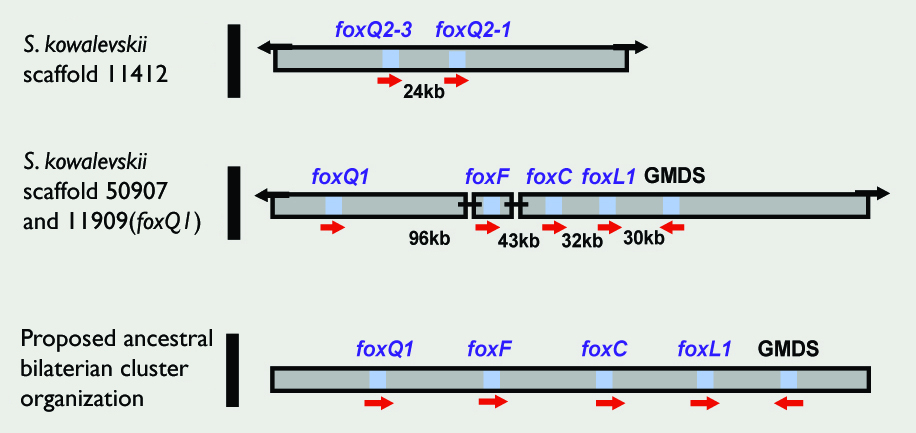

Supplement: Additional file 9: Figure S2 — Fox gene cluster analysis. By using the current S. kowalevskii genome assembly at Baylor College of Medicine (BCM), the HudsonAlpha assembly, HudsonAlpha Institute for Biotechnology, AL (unpublished data), as well as by performing manual genome walks and bidirectional blasts we were able to identify two Fox clusters, a foxQ2-1 - foxQ2-3 cluster and a foxQ1-foxF-foxC-foxL1 cluster. foxC and foxL1 are joined on one scaffold and foxQ2-1 and foxQ2-3 are closely linked on a separate scaffold. In addition, foxF clusters with the foxC -foxL1 scaffold depending on the algorithm used (it is linked in the BCM assembly but not in the HudsonAlpha assembly). Further, we provide evidence of a link of foxQ1 to the foxF, foxC, and foxL1 containing scaffold by manual genome walking using unassembled trace sequences and by bidirectional blast of the scaffold ends (see Additional file 10: Table S8). However, even though no better match was found in the genome, the scaffold ends mostly contain repeats and a final assignment of foxQ1 and foxF requires further characterization. The S. kowalevskii foxQ2-1 and foxQ2-3 cluster indicates a species-specific tandem duplication event. Red arrows indicate orientation of the genes, black arrows indicate the continuation of a scaffold, and distances are given in kilobase pairs underneath each cluster. Black line connecting foxQ1 and the foxF-foxC-foxL cluster indicates area of manual genome walking. [file 2041-9139-5-17-S9.jpeg]

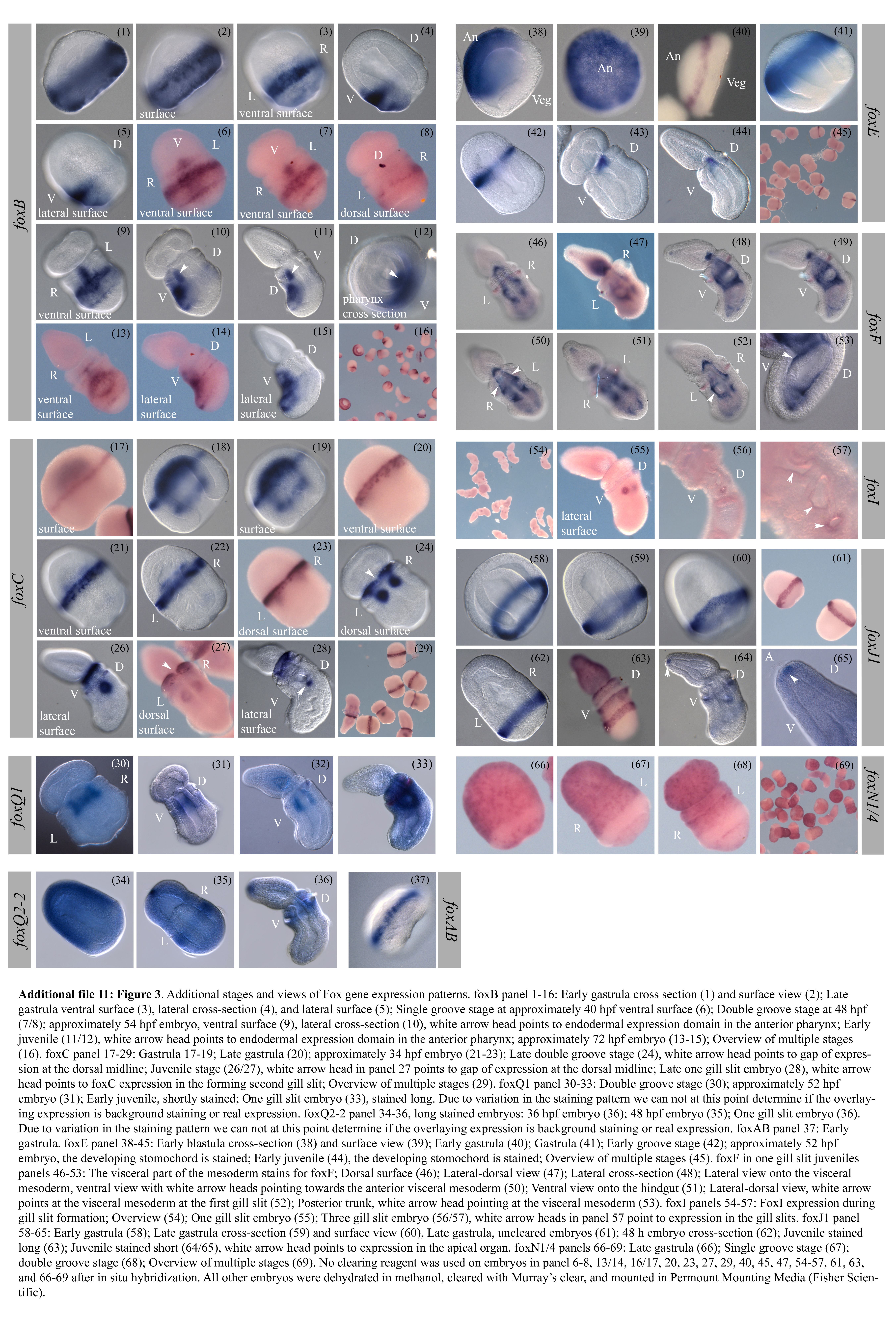

Supplement: Additional file 11: Figure S3 — Additional stages and views of Fox gene expression patterns. [file 2041-9139-5-17-S11.jpg]
