# Supplementary material for: The Fox/Forkhead transcription factor family of the hemichordate Saccoglossus kowalevskii
Source: EvoDevo. 2014 May 7;5:17. doi: 10.1186/2041-9139-5-17 (PMC4077281; doi:10.1186/2041-9139-5-17)
Supplement: Additional file 8: Table S7 — Alignment for Additional file 1: Figure S1 (FoxAB family). [file 2041-9139-5-17-S8.pdf]

Additional Table 7. Alignment for Additional Figure 1 (FoxAB family)

```
LottiaQ2-2 ISMVKEPSKT KPSHSYIALI SMAILESSEK KLLLGDIYEY IMEKFPYF-----NNQEKAW RNSIRHNLSL NECFIKNGRS DN---GKGNF WSIHPACL--EDFSKGDFRR RQARRRARRT LPQMPARNPD IVYGT-
LottiaQ2-3 GDQEPTYGSS KPPLSYIALI SMAILDSADK QTLLGDIYQF IMDKFPYY-----NNKGKAW RNSIRHNLSL NECFIKSGRA EN---GRGNF WSIHPACI--EDFSKGDFRR RKARRRARNS GLLDISEMP- LSYR--
SkFoxQ2-1  QTNSDSNQSS KPTHSYIALI SMAILSTSER KMLLSEIYKY IMNNFPYY-----RNKEKSW RNSVRHNLSL NECFIKNGRS YN---GKGNY WSIHAACE--EDFAKGDFRR RRARRRVRKC HRDEELIAMR TSYGYS
NvFox4fl   ETIVDDDADV KPAHSYIALI AMAILSNSSK KMILGDIYQY ISDNFPYY-----RNKDKSW RNSIRHNLSL NECFIKAGRS EN---GKGNY WAIHPANL--EDFANGDFRR RRARRRVRKS NALKYGVSAG YPYFRS
SkFoxQ2-2  DTSTSDASSK KPTESYIALI AKAILSVREQ KMLLCDIYQN IMDIYPFY-----RNNDKSW RNSIRHNLSL NECFIKNGRS ND---GRGNY WSIHPANL--EDFVKGDFRR RKARRRVRQC YDMVNAVYCH HPFAAA
SpFoxQ2    SICDSPPSTK KPPHSYIALI AMAIINSQDK HLLLCDIYEY IMKRFPFF-----KDNERSW RNSIRHNLSL NECFIKAGRS GD---GRGHF WAIHPANL--EDFARGDYRR RQARRRAR-- -SVSYSPYAY PSAIPS
SeaStarQ2  NKKSDTDEHK KPPHSYISLI AMAILASPEK RLLLCDIYQY IQENYPYY-----RNNDRSW RNSIRHNLSL NECFIKYGRS GD---GRGNF WAVHPANV--EDFSRGDFHR RRARRRVRAS DMMLHGYSV YHPYAAP
BfFoxQ2a   ESPESPSELE KPRHSYIALI AMAIMSSKDK RLLLGDIYQW IMDNFPFY-----RNNERSW RNSIRHNLSL NDCFIKAGRS QD---GKGNY WAIHPANM--EDFSRGDFHR RRARRNVRRY TAMAQHPYMQ YGYQYA
DrFoxQ2    THVKSEGTDE KPAQSYIALI SMAILDSDEK KLLLCDIYQW IMDHYPYF-----KSKDKNW RNSVRHNLSL NECFIKAGRS DN---GKGHF WAIHPANF--QDFSNGDYHR RRARRRIRRV TGQLPYALPA HYQTLG
OryzaQ2    PSTNPEKSAD KPNQSYIALI SRAILSSKEK KLLLCDIYQW IMDHYPYF-----KSKDKNW RNSVRHNLSL NECFIKAGRS DN---GKGHF WAIHPGNY--QDFSKGDYHC RRARR----- ---------- ------
Fugu       TCSGPENSAD KPNQSYIALI SKAILASEQK KLLLCDIYQW IMDHFPYF-----KSKDKNW RNSVRHNLSL NDCFIKAGRS DN---GKGHF WAIHPSNY--QDFSNGDYHC RRARR----- ---------- ------
OaFoxQ2    SEPGGQASLR KPGQSYVALI STAILASPKR KLLLSDIYQW IMDTYPYF-----KNQEKSW RNSIRHNLSL NECFVKAGRS DS---GKGHF WTIHPANL--EDFAKGEYHR QRARSQLRRM AVNLRLCQPR TFYGLR
ChFoxQ2a   ETQQGKKEDV KPTQSYIALI ATAILKSKDK RLVLSDIYKY ILDNYSYF-----QSQDKSW RNSIRHNLSL NECFIKVGRS E----GKGHY WAIHPANY--DDFSQGDFRR RRARRRVRRS FMDTHPYTGY PLRPYS
PediculusQ HNRVFQPEEP KPQHSYIGLI AMAILSSPEG KLVLSDIYQY ILDNYPYF-----RSRGPGW RNSIRHNLSL NDCFVKAGRS AN---GKGHY WAIHPANV--EDFKKGDFRR RKAQRKVRKH MGLAVDEEPD DSPSPP
TcFoxQ2    FPRALQPEEP KPQHSYIGLI AMAILSSPEG KLVLSDIYQH ILDHYPYF-----RTRGPGW RNSIRHNLSL NDCFIKAGRS AN---GKGHY WAIHPANV--DDFRKGDFRR RKAQRKVRKH MGLAVDEDGA DSPSPP
DmFoxQ2    TQRIFQPEEP KPQHSYIGLI AMAILSSTDM KLVLSDIYQY ILDNYPYF-----RSRGPGW RNSIRHNLSL NDCFIKSGRS A----GKGHY WAIHPANM--EDFRKGDFRR RKAQRKVRKH MGLSVDDAST DSPSPP
NasopiaQ2  PHHQQVPEEP KPQHSYIGLI AMAILSSPEK KLVLSDIYQH ILEHYPYF-----RRRGPGW RNSIRHNLSL NDCFVKSGRS AN---GKGHY WAIHPANL--EDFRRGDFRR RKAQRKVRRH MGLAVDEE-P DSPSPP
SkFoxQ2-3  PRARLIHEEP KPSHSYIGLI AMAILKSKDR KMVLSDIYQY ILDNYPYF-----RARGPGW RNSIRHNLSL NDCFVKAGRS AN---GKGHY WAIHPANI--DDFTKGDFRR RRAQRKVRKH MGLSVPDD-E DSPSPP
LottiaQ2-1 LRARYIQEEP KPSQSYIGLI SMAILGSKDK KLILSDIYQW ILDNYAYF-----RTRGPGW RNSIRHNLSL NDCFIKSGRS AN---GKGHY WAIHPANL--DDFSRGDFRR RRAQRKVRKH MGLAVPDD-E DSPSPS
BfFoxQ2c   SFGPIPHEEP KPSHSYIGLI AMAIMSSKEK KLVLSDIYKY ILDNYPYF-----RNRGPGW RNSIRHNLSL NDCFVKMGRS AN---GKGHF WAVHPANV--DDFAQGDFRR RRAQRKVRKH MGLLPEDDGN SSSGSS
CeFkh10    IMSPTECQQP KPQHSYIGLI AMAILSSPQK KMVLAEVYEW IMNEYPYF-----RSRGAGW RNSIRHNLSL NDCFVKAGRA AN---GKGHY WAVHPACV--KDFERGDFRR RRAQRKVRRH MGLQVEDGDS SDEEGS
ChFoxQ2b   GSDDEGTTDG KPNHSYISLI ANAILSSKEK RLVLSDIYQF VLDTQPYF-----RKAGQGW RNSIRHNLSL NECFVKAGRS -P---GKGHF WAINPANF--DDFSKGDFRR RRAQRRARRA MAFGDLIEHF GPYWSS
HmFoxQ2b   HRINNELFNN KPSHSYISLI ANAILASPDK RLVLSDIYKY VLERYDYF-----KKKGSGW RNSIRHNLSL NDCFIKAGRS PN---GKGHY WAINTANY--EDFARGDFRR RRVQRRVRRG VSSPTYPPYA INYYPF
NvFoxQ2b   FNSFVTYSHE KPNQSYISLI SEAILSSPEQ KLILSDIYNF ILTRYPYF-----RTKGTGW RNSIRHNLSL NECFVKAGRS PN---GKGHF WAIDATYF--DDFRRGDFRK RRSYRRYKSK ISNRGRVDDA --FEGK
NvFox2fl   VFGYHVTEEE KPSQSYIGLI GKAIMSVPQK KLVLSDIYNY ILTHYPYF-----RNKGAGW RNSIRHNLSL NECFVKVGRS SN---GKGHF WAINPENY--EDFSKGEYRR KRVSKKRTAS TDGVARTSEK DRLVEK
DmFoxO     ---------- WGNLSYADLI THAIGSATDK RLTLSQIYEW MVQNVPYFKDKGDSNSSAGW KNSIRHNLSL HNRFMRVQNE GT---GKSSW WMLN---------------- ---------- ---------- ------
SpFoxO     ---------- WGNLSYADLI TKAIQSAPDQ RLTLSQIYDW MVKNVPFFKDKGDSNSSAGW KNSIRHNLSL HSRFVRVQNE GT---GKSSW GXST---------------- ---------- ---------- ------
NvFoxO_2   ---------- WGNYSYADLI TQAIQSSPEK RLTLSQIYDW MVNSVPYFRDKGDSNSSAGW KNSIRHNLSL HSKFVRVQNE GN---GKSSW WVLN---------------- ---------- ---------- ------
```
