# Supplementary material for: The Fox/Forkhead transcription factor family of the hemichordate Saccoglossus kowalevskii
Source: EvoDevo. 2014 May 7;5:17. doi: 10.1186/2041-9139-5-17 (PMC4077281; doi:10.1186/2041-9139-5-17)
Supplement: Additional file 10: Table S8 — Bridging contigs for foxQ1 and foxL1-foxF contig. [file 2041-9139-5-17-S10.pdf]

Additional Table 8. Bridging contigs for *foxQ1* and *foxL1-foxF* contig

The following contigs from the NCBI shot gun archive were used to bridge the *foxQ1* containing scaffold with the *foxL1-foxF-foxC* containing scaffold:
224967454.scf; 228746148.scf; 229568878.scf; 230296516.scf; 230570927.scf; 231696822.scf; 231963681.scf; 232154360.scf; 232822186.scf; 233077898.scf; 233367797.scf
