# Supplementary material for: The Fox/Forkhead transcription factor family of the hemichordate Saccoglossus kowalevskii
Source: EvoDevo. 2014 May 7;5:17. doi: 10.1186/2041-9139-5-17 (PMC4077281; doi:10.1186/2041-9139-5-17)
Supplement: Additional file 12 — Discussing various Fox gene expression patterns and their potential evolutionary relevance. [file 2041-9139-5-17-S12.pdf]

Additional Comments

Synexpression of *foxA, foxAB, foxC, foxE,* and *foxL1* in the anterior collar groove ectoderm

foxA, foxAB, *foxC, foxE,* and *foxL1* show circumferential ectodermal expression in the anterior collar groove in *S. kowalevskii* (Figure 2 (9/10), 2 (12-15), 2 (22-25), 2 (32/33), and Figure 3 (29/30)). Comparative literature for all of these genes except FoxA outside chordates is sparse, and no co-expression could be identified in sea urchins. It is therefore difficult to judge at this point if synexpression of these genes was present in the deuterostome or bilaterian ancestor or whether it is a *S. kowalevskii*-specific innovation.

Lateral endoderm and mesoderm patterning (*foxA, foxQ1*)

The endodermal expression of *S. kowalevskii foxA* is very similar to that of the indirect developing hemichordate *Ptychodera flava* [1] during early development. In late larval development (> 8 days), *foxA* is expressed only in the foregut of the *P. flava* larva whereas it is expressed, with the exception of the gill pouches, in the entire endoderm in *S. kowalevskii*. FoxA expression has been observed in several species in the foregut and hindgut and seems to have an evolutionary conserved function in gut development (For an extensive review on this topic see references: [2-4]. Expression of FoxA can further be found in the pharynx of cnidarians [5, 6] indicating a deep evolutionary conservation for this gene in intestinal patterning.

FoxQ1 is a conserved pharyngeal endoderm marker and is also expressed in the prospective pharynx and later in the pharyngeal pouches of frogs [7], the pharyngeal gills in the urochordates *C. intestinalis* [8], and in the pharyngeal endoderm in the lophotrochozoan

C. teleta [9]. It further marks the endostyle in *B. floridae* [10] and in *C. intestinalis* [8]. It is not, however, expressed in the gills in the cephalochordate *B. floridae* [10]. The expression of *S. kowalevskii foxQ1* in the gill pouches forming endoderm reflects the ancestral expression of FoxQ1 in different organisms.

***foxE* (pharyngeal endoderm)**

In chordates, FoxE (previously called "thyroid transcription factor 2") together with FoxQ1 are markers for the endostyle, an endodermal derived structure found in urochordates, cephalochordates, and lampreys, which evolved into the thyroid in vertebrates [7, 8, 11-13]. In cephalochordates FoxE is not expressed in the endostyle but it is in the club shaped gland [12]. It is proposed that the club shaped gland was present in the common deuterostome ancestor and that FoxE expression got co-opted into the endostyle when the club shaped gland got lost during evolution in the chordate lineage [12]. In *S. kowalevskii*, *foxE* is expressed in the pharyngeal endoderm (Figure 2 (35)) mostly anterior to *foxQ1* expression which is expressed in the anterior pharynx (Figure 4 (14/15)). Only a small domain of co-expression can be observed directly anterior to the gill buds. *S. kowalevskii foxE* seems to be also expressed in the forming stomochord in the anterior dorsal pharynx endoderm.

***foxG* (dorsal endoderm and mesoderm expression)**

Next to its ectodermal expression is *foxG* expressed preferentially on the dorsal side of the *S. kowalevskii* endoderm and mesoderm (Figure 3 (10)). The dorsal mesoderm expression might be a hemichordate-specific trait.

Fox genes with conserved neuronal functions (*foxD, foxG, foxN1/4*, *foxP)*

Certain Fox genes are known for their function in vertebrate neural development; FoxD, FoxG, FoxP, and FoxN1/4 [14-21]. In *S. kowalevskii*, the orthologs for these Fox genes show single cell expression or high level expression in single cells on top of a ubiquitous expression domain in the ectoderm, raising the possibility that they also play a role in neural patterning in *S. kowalevskii.* However, co-localization studies with neural markers are needed to confirm this.

FoxD is an important gene in notochord development [16, 17], specification of the diencephalon (reviewed in [17]), and neural crest formation in chordates [17, 22-30] (reviewed in [17, 19]). In the cephalochordate *B. floridae*, neural *foxD* expression is detected in the anterior neural plate, the anterior somites, the neural tube, and later in the cerebral vesicle [17, 31-36]. These expression domains look remarkably similar to the anterior *foxD* expression domain of the urochordate *Ciona* [16]. FoxD is also an important marker gene for neural crest development in vertebrates, a cell type whose evolutionary origin is highly debated. It is viewed as a feature distinguishing vertebrates from protochordates [37-40] since no neural crest cells could be found in basal chordates like *B. floridae* [17, 36, 41, 42] and the presence of neural crest-like cells in urochordates is controversial [40, 43]. It is therefore not likely that the single cells expressing *foxD* in *S. kowalevskii*, anterior to the ectodermal circumferential ring expression of *foxD,* are homologs to neural crest cells. In this respect however, it will be interesting to analyze the regulatory environment of *foxD* in *S. kowalevskii* to better understand the regulatory changes that have occurred during neural crest evolution.

The FoxN group is of interest since members of this group have crucial roles in mammalian development. *foxN1* is essential for proper immune response in mice [44] and

a downstream target of the Wnt-pathway [45]. *foxN4* is involved in specifying amacrine and horizontal cells in the retina and is upstream of the bHLH gene Math3, NeuroD1, and Prox1 [46-48] (reviewed in [49]). It is further necessary for the development of V2a and V2b interneurons in the spinal cord using lateral inhibition via the Delta-Notch pathway by activating the transcription of Delta4 and the bHLH gene Mash-1 [20, 50]. In *S. kowalevskii*, *foxN1/4* is expressed ubiquitously with high levels of expression in single cells, which indicates that in *S. kowalevskii*, FoxN1/4 may also be involved in neural development. To what extent *foxN1/4* interacts in *S. kowalevskii* with the Delta -Notch pathway, bHLH factors, or the Wnt-pathway to specify neuronal subtypes will be interesting to test in future experiments.

Mouse *foxP1* and *foxP2* are expressed during brain development - *foxP1* in the basal ganglia, cerebral cortex, cerebellum, and thalamus; *foxP2* in the cerebral cortex, hippocampus, and thalamus [51], as are FoxP genes from *Xenopus* and zebrafish [52-54]. In the fish *Medaka foxP1* expression indicates a role during striatum projection neuron development [55] and basal ganglia development of the developing central nervous system [56]. Mutations in the human *foxP2* gene lead to severe language disorders [21, 57, 58] (reviewed in: [53, 59]). In the urochordate *C. intestinalis*, *foxP* is also expressed in the developing brain [60]. Outside chordates, FoxP expression is only known from sea urchins and flies. In *D. melanogaster* the two splice variants of FoxP (*fd85Ea* and *fd85Eb*) are expressed in the developing CNS [61, 62], while in sea urchins, *foxP* is only expressed in the fore- and mid-gut of the larva. A FoxP ortholog is also found in other ecdysozoans, cnidarians, and sponges [63, 64], but expression patterns are not yet reported. The single

cells with high levels of *foxP* expression in the *S.kowalevskii* proboscis (Figure 4 (7-9)) would support a conserved function in neural development.

Recent literature has proposed a more centralized organization of the hemichordate nervous system focused in the dorsal collar cord, so it is interesting that no obvious expression of Fox genes with conserved neural functions in chordates and protostomes were detected in the dorsal or ventral nerve cord of *S. kowalevskii* during development up to the first gill slit stage when cords are already forming, but are rather expressed in circumferential patterns in the ectoderm. Further expression studies in adult nervous system will be required to test whether these genes play a later role in cord formation, or play a conserved role in the development of the nerve plexus. It will be interesting to investigate the spatial arrangement of neuronal subtypes by analyzing the neural gene regulatory network in *S. kowalevskii* and especially the role of bHLH factors, another large transcription factor family known for their conserved role in neural induction and neural subtype specification.

f*oxD, foxG* (Diencephalon/ telencephalon evolution)

FoxG is known for its role in brain development in vertebrates, and is expressed in the telencephalon [65-67] (reviewed in [14]) whereas FoxD is know for its role in diencephalon development in vertebrates (reviewed in [17]).

In the cephalochordate *B. floridae however*, *foxD* and *foxG* are co-expressed in the cerebral vesicle [14, 17, 68], a structure proposed to be homolog to the vertebrate diencephalon [17]. In *S. kowalevskii*, *foxD* and *foxG* show next to other expression domains high levels of expression at the proboscis base, an area of the body plan that by

synexpression analysis [69] is equivalent to the forebrain region of chordates (Figure 2 (30) indicated with star, Figure 3 (6-10)) (Discussion and partial expression of *foxG/bf1* can be found in [69]. This could suggest that in the deuterostome ancestor, FoxD and FoxG may have been co-expressed and a segregation of the expression domains into the diencephalon and telencephalon is vertebrate specific.

References for Additional Comments:

1. Taguchi S, Tagawa K, Humphreys T, Nishino A, Satoh N, and Harada Y: **Characterization of a hemichordate fork head/HNF-3 gene expression.** *Dev Genes Evol* 2000, **210**:11-7.
2. Boyle MJ, and Seaver EC: **Developmental expression of foxA and gata genes during gut formation in the polychaete annelid, Capitella sp. I.** *Evol Dev* 2008, **10**:89-105.
3. Boyle MJ, and Seaver EC: **Expression of FoxA and GATA transcription factors correlates with regionalized gut development in two lophotrochozoan marine worms: *Chaetopterus* (Annelida) and *Themiste lageniformis* (Sipuncula).** *Evodevo* 2010, **1**:2.
4. Fuchs J, Martindale MQ, and Hejnol A: **Gene expression in bryozoan larvae suggest a fundamental importance of pre-patterned blastemic cells in the bryozoan life-cycle.** *Evodevo* 2011, **2**:13.
5. Fritzenwanker JH, Saina M, and Technau U: **Analysis of forkhead and snail expression reveals epithelial-mesenchymal transitions during embryonic and larval development of *Nematostella vectensis.*** *Dev Biol* 2004, **275**:389-402.
6. Martindale MQ, Pang K, and Finnerty JR: **Investigating the origins of triploblasty: 'mesodermal' gene expression in a diploblastic animal, the sea anemone *Nematostella vectensis* (phylum, Cnidaria; class, Anthozoa).** *Development* 2004, **131**:2463-74.
7. Choi VM, Harland RM, and Khokha MK: **Developmental expression of FoxJ1.2, FoxJ2, and FoxQ1 in *Xenopus tropicalis.*** *Gene Expr Patterns* 2006, **6**:443-7.
8. Ogasawara M, and Satou Y: **Expression of FoxE and FoxQ genes in the endostyle of Ciona intestinalis.** *Dev Genes Evol* 2003, **213**:416-9.
9. Shimeld SM, Boyle MJ, Brunet T, Luke GN, and Seaver EC: **Clustered Fox genes in lophotrochozoans and the evolution of the bilaterian Fox gene cluster.** *Dev Biol* 2010, **340**:234-48.
10. Mazet F, Luke GN, and Shimeld SM: **The amphioxus FoxQ1 gene is expressed in the developing endostyle.** *Gene Expr Patterns* 2005, **5**:313-5.
11. Mazet F: **The Fox and the thyroid: the amphioxus perspective.** *Bioessays* 2002, **24**:696-9.
12. Yu JK, Holland LZ, Jamrich M, Blitz IL, and Holland ND: **AmphiFoxE4, an amphioxus winged helix/forkhead gene encoding a protein closely related to

vertebrate thyroid transcription factor-2: expression during pharyngeal development. *Evol Dev* 2002**, 4**:9-15.

13. Hiruta J, Mazet F, Yasui K, Zhang P, and Ogasawara M: **Comparative expression analysis of transcription factor genes in the endostyle of invertebrate chordates.** *Dev Dyn* 2005**, 233**:1031-7.
14. Toresson H, Martinez-Barbera JP, Bardsley A, Caubit X, and Krauss S: **Conservation of BF-1 expression in amphioxus and zebrafish suggests evolutionary ancestry of anterior cell types that contribute to the vertebrate telencephalon.** *Dev Genes Evol* 1998**, 208**:431-9.
15. Hardcastle Z, and Papalopulu N: **Distinct effects of XBF-1 in regulating the cell cycle inhibitor p27(XIC1) and imparting a neural fate.** *Development* 2000**, 127**:1303-14.
16. Imai KS, Satoh N, and Satou Y: **An essential role of a FoxD gene in notochord induction in Ciona embryos.** *Development* 2002**, 129**:3441-53.
17. Yu JK, Holland ND, and Holland LZ: **An amphioxus winged helix/forkhead gene, AmphiFoxD: insights into vertebrate neural crest evolution.** *Dev Dyn* 2002**, 225**:289-97.
18. Danilova N, Visel A, Willett CE, and Steiner LA: **Expression of the winged helix/forkhead gene, foxn4, during zebrafish development.** *Brain Res Dev Brain Res* 2004**, 153**:115-9.
19. Pohl BS, and Knöchel W: **Of Fox and Frogs: Fox (fork head/winged helix) transcription factors in *Xenopus* development.** *Gene* 2005**, 344**:21-32.
20. Del Barrio MG, Taveira-Marques R, Muroyama Y, Yuk DI, Li S, Wines-Samuelson M, Shen J, Smith HK, Xiang M, Rowitch D, and Richardson WD: **A regulatory network involving Foxn4, Mash1 and delta-like 4/Notch1 generates V2a and V2b spinal interneurons from a common progenitor pool.** *Development* 2007**, 134**:3427-36.
21. Takahashi H, Takahashi K, and Liu FC: **FOXP genes, neural development, speech and language disorders.** *Adv Exp Med Biol* 2009, **665**:117-29.
22. Dirksen ML, and Jamrich M: **Differential expression of fork head genes during early *Xenopus* and zebrafish development.** *Dev Genet* 1995**, 17**:107-16.
23. Sasai N, Mizuseki K, and Sasai Y: **Requirement of FoxD3-class signaling for neural crest determination in *Xenopus.*** *Development* 2001**, 128**:2525-36.
24. Pohl BS, and Knöchel W: **Overexpression of the transcriptional repressor FoxD3 prevents neural crest formation in *Xenopus* embryos.** *Mech Dev* 2001**, 103**:93-106.
25. Pohl BS, and Knöchel W: **Temporal and spatial expression patterns of FoxD2 during the early development of *Xenopus laevis.*** *Mech Dev* 2002**, 111**:181-4.
26. Lister JA, Cooper C, Nguyen K, Modrell M, Grant K, and Raible DW: **Zebrafish Foxd3 is required for development of a subset of neural crest derivatives.** *Dev Biol* 2006**, 290**:92-104.
27. Stewart RA, Arduini BL, Berghmans S, George RE, Kanki JP, Henion PD, and Look AT: **Zebrafish foxd3 is selectively required for neural crest specification, migration and survival.** *Dev Biol* 2006**, 292**:174-88.
28. Sauka-Spengler T, and Bronner-Fraser M: **Insights from a sea lamprey into the evolution of neural crest gene regulatory network.** *Biol Bull* 2008**, 214**:303-14.

29. Curran K, Raible DW, and Lister JA: **Foxd3 controls melanophore specification in the zebrafish neural crest by regulation of Mitf.** *Dev Biol* 2009**, 332**:408-17.
30. Curran K, Lister JA, Kunkel GR, Prendergast A, Parichy DM, and Raible DW: **Interplay between Foxd3 and Mitf regulates cell fate plasticity in the zebrafish neural crest.** *Dev Biol* 2010**, 344**:107-18.
31. Lacalli TC, Holland ND, and West JE: **Landmarks in the anterior central nervous system of amphioxus larvae.** *Philosophical Transactions B* 1994**, 344**:165.
32. Holland ND, Panganiban G, Henyey EL, and Holland LZ: **Sequence and developmental expression of AmphiDll, an amphioxus Distal-less gene transcribed in the ectoderm, epidermis and nervous system: insights into evolution of craniate forebrain and neural crest.** *Development* 1996**, 122**:2911-20.
33. Lacalli TC: **Frontal eye circuitry, rostral sensory pathways and brain organization in amphioxus larvae: evidence from 3D reconstructions.** *Philosophical Transactions B* 1996**, 351**:243.
34. Williams NA, and Holland PWH: **Old head on young shoulders.** *Nature* 1996**, 383**:490.
35. Holland LZ, Kene M, Williams NA, and Holland ND: **Sequence and embryonic expression of the amphioxus engrailed gene (AmphiEn): the metameric pattern of transcription resembles that of its segment-polarity homolog in *Drosophila.*** *Development* 1997**, 124**:1723-32.
36. Holland LZ, and Holland ND: **Evolution of neural crest and placodes: amphioxus as a model for the ancestral vertebrate?** *J Anat* 2001**, 199**:85-98.
37. Gans C, and Northcutt RG: **Neural crest and the origin of vertebrates: a new head.** *Science* 1983**, 220**:268-73.
38. Glenn Northcutt R: **The new head hypothesis revisited.** *J Exp Zool B Mol Dev Evol* 2005**, 304**:274-97.
39. Butler AB: **The serial transformation hypothesis of vertebrate origins: comment on "The new head hypothesis revisited".** *J Exp Zool B Mol Dev Evol* 2006**, 306**:419-24.
40. Jeffery WR: **Chordate ancestry of the neural crest: new insights from ascidians.** *Semin Cell Dev Biol* 2007**, 18**:481-91.
41. Meulemans D, and Bronner-Fraser M: **Amphioxus and lamprey AP-2 genes: implications for neural crest evolution and migration patterns.** *Development* 2002**, 129**:4953-62.
42. Yu JK, Meulemans D, McKeown SJ, and Bronner-Fraser M: **Insights from the amphioxus genome on the origin of vertebrate neural crest.** *Genome Res* 2008**, 18**:1127-32.
43. Jeffery WR, Chiba T, Krajka FR, Deyts C, Satoh N, and Joly JS: **Trunk lateral cells are neural crest-like cells in the ascidian Ciona intestinalis: insights into the ancestry and evolution of the neural crest.** *Dev Biol* 2008**, 324**:152-60.
44. Nehls M, Pfeifer D, Schorpp M, Hedrich H, and Boehm T: **New member of the winged-helix protein family disrupted in mouse and rat nude mutations.** *Nature* 1994**, 372**:103-7.

45. Balciunaite G, Keller MP, Balciunaite E, Piali L, Zuklys S, Mathieu YD, Gill J, Boyd R, Sussman DJ, and Holländer GA: **Wnt glycoproteins regulate the expression of FoxN1, the gene defective in nude mice.** *Nat Immunol* 2002**, 3**:1102-8.
46. Gouge A, Holt J, Hardy AP, Sowden JC, and Smith HK: **Foxn4--a new member of the forkhead gene family is expressed in the retina.** *Mech Dev* 2001**, 107**:203-6.
47. Li S, Mo Z, Yang X, Price SM, Shen MM, and Xiang M: **Foxn4 controls the genesis of amacrine and horizontal cells by retinal progenitors.** *Neuron* 2004**, 43**:795-807.
48. Kunzevitzky NJ, Almeida MV, Duan Y, Li S, Xiang M, and Goldberg JL: **Foxn4 is required for retinal ganglion cell distal axon patterning.** *Mol Cell Neurosci* 2011**, 46**:731-41.
49. Kay JN, and Baier H: **Out-foxing fate; molecular switches create neuronal diversity in the retina.** *Neuron* 2004**, 43**:759-60.
50. Li S, Misra K, Matise MP, and Xiang M: **Foxn4 acts synergistically with Mash1 to specify subtype identity of V2 interneurons in the spinal cord.** *Proc Natl Acad Sci U S A* 2005**, 102**:10688-93.
51. Ferland RJ, Cherry TJ, Preware PO, Morrisey EE, and Walsh CA: **Characterization of Foxp2 and Foxp1 mRNA and protein in the developing and mature brain.** *J Comp Neurol* 2003**, 460**:266-79.
52. Bonkowsky JL, and Chien CB: **Molecular cloning and developmental expression of foxP2 in zebrafish.** *Dev Dyn* 2005**, 234**:740-6.
53. Pohl BS, Rössner A, and Knöchel W: **The Fox gene family in *Xenopus laevis*: FoxI2, FoxM1 and FoxP1 in early development.** *Int J Dev Biol* 2005**, 49**:53-8.
54. Schon C, Wochnik A, Rossner A, Donow C, and Knochel W: **The FoxP subclass in *Xenopus laevis* development.** *Dev Genes Evol* 2006**, 216**:641-6.
55. Tamura S, Morikawa Y, Miyajima A, and Senba E: **Expression of oncostatin M in hematopoietic organs.** *Dev Dyn* 2002**, 225**:327-31.
56. Shen X, Cui J, and Nagahama Y: **The forkhead gene family in medaka: expression patterns and gene evolution.** *Cytogenet Genome Res* 2012**, 136**:123-30.
57. Lai CS, Fisher SE, Hurst JA, Vargha-Khadem F, and Monaco AP: **A forkhead-domain gene is mutated in a severe speech and language disorder.** *Nature* 2001**, 413**:519-23.
58. Fisher SE, and Scharff C: **FOXP2 as a molecular window into speech and language.** *Trends Genet* 2009**, 25**:166-77.
59. Hannenhalli S, and Kaestner KH: **The evolution of Fox genes and their role in development and disease.** *Nat Rev Genet* 2009**, 10**:233-40.
60. Imai KS, Hino K, Yagi K, Satoh N, and Satou Y: **Gene expression profiles of transcription factors and signaling molecules in the ascidian embryo: towards a comprehensive understanding of gene networks.** *Development* 2004**, 131**:4047-58.
61. Lee HH, and Frasch M: **Survey of forkhead domain encoding genes in the *Drosophila* genome: Classification and embryonic expression patterns.** *Dev Dyn* 2004**, 229**:357-66.

62. Santos ME, Athanasiadis A, Leitão AB, DuPasquier L, and Sucena E: **Alternative splicing and gene duplication in the evolution of the FoxP gene subfamily.** *Mol Biol Evol* 2011**, 28**:237-47.
63. Larroux C, Fahey B, Liubicich D, Hinman VF, Gauthier M, Gongora M, Green K, Wörheide G, Leys SP, and Degnan BM: **Developmental expression of transcription factor genes in a demosponge: insights into the origin of metazoan multicellularity.** *Evol Dev* 2006**, 8**:150-73.
64. Shimeld SM, Degnan B, and Luke GN: **Evolutionary genomics of the Fox genes: Origin of gene families and the ancestry of gene clusters.** *Genomics* 2010**, 95**:256-60.
65. Papalopulu N, and Kintner C: **A posteriorising factor, retinoic acid, reveals that anteroposterior patterning controls the timing of neuronal differentiation in *Xenopus* neuroectoderm.** *Development* 1996**, 122**:3409-18.
66. Hébert JM, and Fishell G: **The genetics of early telencephalon patterning: some assembly required.** *Nat Rev Neurosci* 2008**, 9**:678-85.
67. Roth M, Bonev B, Lindsay J, Lea R, Panagiotaki N, Houart C, and Papalopulu N: **FoxG1 and TLE2 act cooperatively to regulate ventral telencephalon formation.** *Development* 2010**, 137**:1553-62.
68. Yu JK, Holland ND, and Holland LZ: **Tissue-specific expression of FoxD reporter constructs in amphioxus embryos.** *Dev Biol* 2004**, 274**:452-61.
69. Lowe CJ, Wu M, Salic A, Evans L, Lander E, Stange-Thomann N, Gruber CE, Gerhart J, and Kirschner M: **Anteroposterior patterning in hemichordates and the origins of the chordate nervous system.** *Cell* 2003**, 113**:853-65.
